# NADPH oxidase 1/4 dual inhibition impairs transforming growth factor-beta protumorigenic effects in cholangiocarcinoma cancer-associated fibroblasts

**DOI:** 10.1038/s41392-025-02347-z

**Published:** 2025-08-18

**Authors:** Josep Amengual, Ester Gonzalez-Sanchez, Mariana Yáñez-Bartolome, Laura Sererols-Viñas, Aashreya Ravichandra, Celia Guiton, Noel P. Fuste, Ania Alay, Sara Hijazo-Pechero, Beatriz Martín-Mur, Marta Gut, Anna Esteve-Codina, Ana Cantos-Cortes, Rut Espinosa-Sotelo, Emilio Ramos, Teresa Serrano, Mariona Calvo, Berta Laquente, Joana Ferrer, Gabriel Pons, Andrés Mendez-Lucas, Steven Dooley, Sumera I. Ilyas, Marie Vallette, Lynda Aoudjehane, Marie Lequoy, Laura Fouassier, Cédric Coulouarn, Silvia Affò, Alexander Scheiter, Diego F. Calvisi, Tian V. Tian, Isabel Fabregat, Javier Vaquero

**Affiliations:** 1 TGF-β and Cancer Group, Oncobell Program, Bellvitge Biomedical Research Institute (IDIBELL), Barcelona, Spain; 2 CIBEREHD, National Biomedical Research Institute on Liver and Gastrointestinal Diseases, Instituto de Salud Carlos III, Madrid, Spain; 3 Department of Physiology and Pharmacology, University of Salamanca, 37007 Salamanca, Spain; 4 HepatoBiliary Tumours Lab, Centro de Investigación del Cáncer and Instituto de Biología Molecular y Celular del Cáncer, CSIC-Universidad de Salamanca, Salamanca, 37007 Spain; 5 Upper Gastrointestinal and Endocrine Tumour Unit, Vall d’Hebron Institute of Oncology (VHIO), Barcelona, Spain; 6 Tumor Microenvironment Plasticity and Heterogeneity Group, Instituto de Investigaciones Biomédicas August Pi i Sunyer (IDIBAPS), Barcelona, Spain; 7 TUM School of Medicine and Health, Department of Clinical Medicine – Clinical Department for Internal Medicine II, University Medical Center, Technical University of Munich, Munich, Germany; 8 Unit of Bioinformatics for Precision Oncology, Catalan Institute of Oncology (ICO), L’Hospitalet de Llobregat, Barcelona, Spain; 9 Preclinical and Experimental Research in Thoracic Tumors (PReTT), Oncobell Program, IDIBELL, L’Hospitalet de Llobregat, Barcelona, Spain; 10 Centro Nacional de Análisis Genómico (CNAG), C/Baldiri Reixac 4, 08028 Barcelona, Spain; 11 University of Barcelona (UB), 08034 Barcelona, Spain; 12 Faculty of Medicine and Health Sciences, University of Barcelona, L’Hospitalet de Llobregat, Barcelona, Spain; 13 Department of Surgery, Liver Transplant Unit, University Hospital of Bellvitge, Barcelona, Spain; 14 Pathological Anatomy Service, University Hospital of Bellvitge, Barcelona, Spain; 15 Oncología Médica, Institut Català d’Oncologia (ICO-IDIBELL), L’Hospitalet del Llobregat, Barcelona, Spain; 16 Barcelona Clinic Liver Cancer (BCLC) group. Institut d’Investigacions Biomèdiques August Pi I Sunyer (IDIBAPS). Surgery Department, Liver Oncology Unit, Hospital Clinic de Barcelona, Barcelona, Spain Barcelona University, Barcelona, Spain; 17 Physiological Sciences Department, University of Barcelona, Barcelona, Spain; 18 Nutrition, metabolism and gene therapy group. Diabetes and Metabolism Program. IDIBELL, L’Hospitalet de Llobregat, Barcelona, Spain; 19 Department of Medicine II, Section Molecular Hepatology, Medical Faculty Mannheim, Heidelberg University, Mannheim, Germany; 20 Division of Gastroenterology and Hepatology, Mayo Clinic, Rochester, Minnesota USA; 21 UMRS_938, Sorbonne Université, Inserm, Centre de Recherche Saint-Antoine (CRSA), Paris, France; 22 ICAN BioCell-Human Liver Biology core, IHU- Foundation for innovation in Cardiometabolism and Nutrition (IHU-ICAN), F-75013 Paris, France; 23 Sorbonne Université, INSERM, UMRS 1166/IHU-ICAN, F-75013 Paris, France; 24 AP-HP Sorbonne Université, Hôpital Universitaire Saint Antoine, Service d´Hépatologie, F-75012 Paris, France; 25 Inserm, Univ Rennes, OSS (Oncogenesis, Stress, Signaling) UMR_S 1242, Centre de Lutte contre le Cancer Eugène Marquis, F-35042 Rennes, France; 26 Institute of Pathology, University of Regensburg, Regensburg, Germany

**Keywords:** Cancer therapy, Cancer microenvironment, Target identification

## Abstract

Transforming growth factor beta (TGF-β) signalling has become an attractive therapeutic target due to its pro-tumorigenic actions on epithelial cells and its immunosuppressive effects in the tumour microenvironment. In intrahepatic cholangiocarcinoma (iCCA), a highly aggressive malignancy of the biliary tract with poor prognosis, the latest clinical trials using TGF-β inhibitors have failed indicating that the specific actions carried out by TGF-β in iCCA are yet not well delineated. Here, we show that TGF-β signalling is highly active in iCCA and exerts a prominent suppressor effect on tumour cell lines and organoids established from iCCA metastases biopsies, that relies on a functional canonical SMAD2/3/4 signalling. Thus, TGF-β inhibitors promote, instead of inhibiting, tumour cell growth. In this context, a promising strategy is to target intracellular proteins downstream the TGF-β receptors accounting only for TGF-β pro-tumorigenic actions. NADPH oxidase 4 (NOX4), a downstream mediator of the TGF-β signalling pathway, is strictly expressed in cancer-associated fibroblasts (CAF) of iCCA and acts in concert with NOX1 to regulate CAF functions. Use of a dual NOX4/NOX1 inhibitor impaired CAF actions and reduced tumour growth in vitro and in two different in vivo iCCA experimental models. Collectively, our findings reveal an actionable way to specifically target TGF-β pro-tumorigenic actions in CAF from iCCA without undesirable side effects on tumour cells, suggesting a potentially bright future for dual NOX4/NOX1 inhibitors in the clinics, alone or in combination with other therapies.

## Introduction

Intrahepatic cholangiocarcinoma (iCCA) is a very aggressive malignancy of the biliary tree with a poor prognosis, mainly due to late diagnosis and resistance to therapies.^1,2^ iCCA is characterised by a prominent tumour microenvironment (TME) enriched with cancer-associated fibroblasts (CAF), which produce abundant collagen and elastic fibres that are deposited in the extracellular matrix (ECM). These components contribute to tumour progression and chemoresistance.^1,2^ For the majority of iCCA patients who are not eligible for surgery, the combination of chemotherapy (gemcitabine plus cisplatin) with immunotherapy (durvalumab or pembrolizumab) was recently approved as the new standard of care.^3–5^ Although the use of immunotherapy represents the first improvement in patient care in many years, this combination still shows limited efficacy in a significant number of patients. Thus, finding new ways to boost the immune response in these unresponsive patients has become essential. In this context, Transforming Growth Factor-beta (TGF-β), which displays potent immunosuppressive activity, has emerged as an attractive target, and the use of TGF-β inhibitors has gained interest. Nevertheless, the latest phase II/III clinical trial (NCT04066491) using a TGF-β inhibitor in combination with chemotherapy failed to meet expectations and has been discontinued,^6,7^ indicating that there are unknown mechanisms compromising the use of TGF-β as a therapeutical target in CCA. Importantly, despite the use of TGF-β inhibitors in CCA clinical trials, there is no evidence supporting their efficacy in preclinical models of CCA.

TGF-β activates a complex signalling pathway that regulates different cellular processes, including proliferation, migration, and cell death.^8^ Thus, TGF-β dysregulation plays an important role in cancer development and progression, but its use as a therapeutic target remains challenging due to its dual ability to both inhibit and promote carcinogenesis. In the liver, as in many other organs, TGF-β acts as a tumour suppressor in the early stages of tumour development, while in advanced stages it exerts tumour-promoting actions.^8^ In addition, TGF-β helps to generate an advantageous TME by activating CAF, inducing angiogenesis and promoting immune suppression and evasion.^8^ Despite being thoroughly investigated in some cancers such as hepatocellular carcinoma (HCC)^9^ and pancreatic cancer,^10^ these processes are still understudied in CCA.^11^

Therefore, the most efficient strategies to successfully target the TGF-β signalling pathway would be those that allow for the preservation of the suppressor effects of TGF-β while impairing its pro-tumorigenic actions. In this context, a promising strategy is to target intracellular proteins downstream of the TGF-β receptors that account solely for TGF-β pro-tumorigenic functions. Among these candidates, NOX4, a member of the NADPH oxidase family, is a downstream mediator of TGF-β signalling in both tumour cells and the TME,^12^ and is a key source of reactive oxygen species (ROS). We have previously shown that NOX4 acts as a negative regulator of hepatocarcinogenesis in tumour cells,^13^ while other studies have demonstrated that NOX4 may be involved in tumour progression and chemoresistance in other tumour types.^14^ Despite its controversial role in tumour cells, most reports support the central and specific role of NOX4 in regulating CAF activation in multiple cancers.^15^ However, recent reports also suggest that NOX4 is dispensable for myofibroblast differentiation.^16^ Interestingly, the functions and therapeutic potential of NOX4 in tumour cells and the TME from iCCA still remain unknown.

Here, we aimed to better understand the role and therapeutic potential of the TGF-β-NOX4 axis in iCCA. Using comprehensive in vitro and in vivo models, we found that TGF-β signalling activation occurs widely across species in iCCA. Our analysis reveals that, regardless of the tumour stage, TGF-β exerts a potent suppressor effect on iCCA tumour cells that is dependent on a functional SMAD2/3/4 complex and that inhibition of TGF-β receptors boosts iCCA progression by promoting tumour cell growth. We also demonstrate that NOX4 is upregulated in iCCA and specifically expressed in CAF. However, NOX4 depletion is insufficient to affect iCCA progression due to a compensatory mechanism driven by NOX1. Accordingly, NOX4/NOX1 dual inhibition impairs CAF functions in vitro and reduces tumour burden in preclinical in vivo models of iCCA.

## Results

### TGF-β signalling is activated in both human and murine CCA samples

**Fig. 1:**
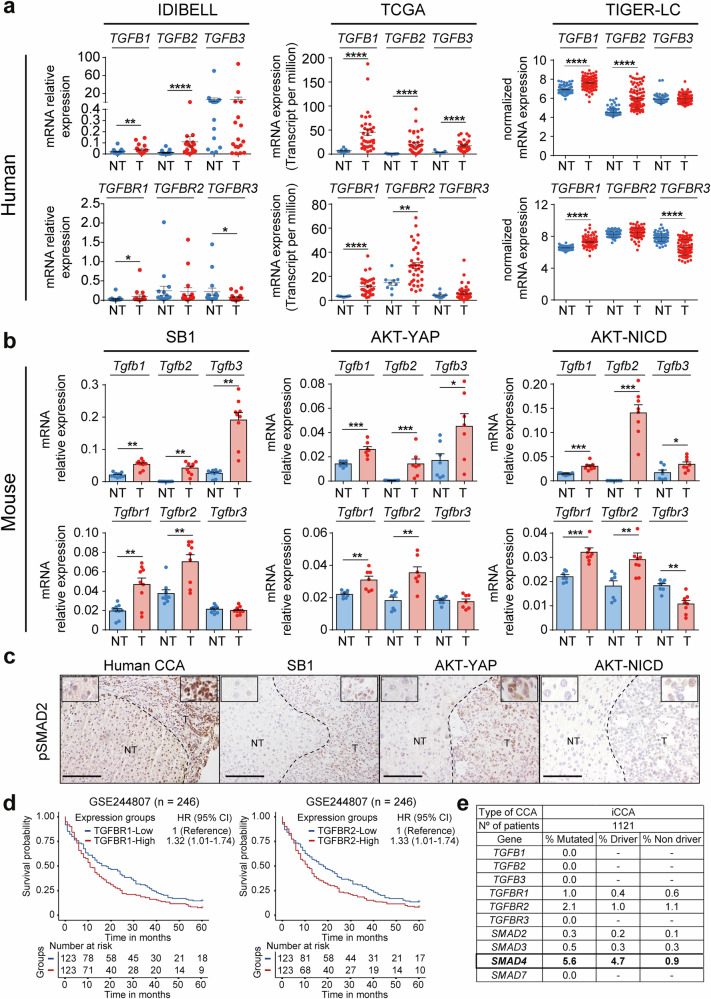
TGF-β signalling is activated and functional in human and mouse intrahepatic cholangiocarcinoma (iCCA) tumours. **a**, **b** TGF-β ligands (*TGFB*) and receptors (*TGFBR*) mRNA expression in CCA tumours compared to surrounding liver tissue from the IDIBELL (*n* = 17), the TCGA (*n* = 36) and TIGER-LC (*n* = 91) cohorts of patients (**a**) and from the SB1 syngeneic orthotopic (*n* = 9) and the AKT-YAP (*n* = 7) and AKT-NICD hydrodynamic tail vein injection iCCA tumour models (**b**). **c** Representative immunohistochemical images showing phophoSMAD2 expression, used as a readout of the activation of the TGF-β signalling pathway, in samples from iCCA patients, and SB1, AKT-YAP and AKT-NICD iCCA animal models. Scale: 100 µm. **d** Kaplan-Meier curve of 5-year overall survival of patients from GSE244807 cohort (*n* = 246) stratified based on *TGFBR1 and TGFBR2* expression (Cox Proportional Hazards model). **e** Mutational status of different members of the TGF-β canonical signalling pathway in iCCA patients. mRNA expression levels were determined by RT-qPCR in samples from the IDIBELL cohort of patients and the animal models. Gene expression data from public data bases was obtained using RNAseq (TCGA cohort) or microarrays (TIGER cohort). Values are expressed as means ± SEM. *p < 0.05; **p < 0.01, ***p < 0.001, ****p < 0.0001 as compared to NT. NT non-tumoral, T tumour

We first analysed the status of the TGF-β signalling pathway in CCA patients in comparison with different murine models of iCCA, because the information about TGF-β in the later species is scarce in the literature. We used state-of-the-art mouse iCCA models,^17^ including the syngeneic orthotopic SB1 model^18^ and the hydrodynamic tail vein injection (HTVI) AKT-YAP, and the AKT-NICD models.^19^ Notably, these mouse models develop iCCA that, like human iCCA, show increased expression of *Krt19*, indicative of malignant cholangiocyte proliferation, and *Acta2* and *Col1a1*, indicative of a well-formed desmoplastic stroma with a high content of CAF (Supplementary Fig. 1a, b). We therefore compared them with data from iCCA patients from our own cohort of patients (IDIBELL cohort) and two public databases (TCGA and TIGER-LC cohorts). Our analyses revealed a significant increase in mRNA expression of *TGFB1-2* ligands and *TGFBR1* in iCCA compared to non-tumoral tissue, both in human cohorts (IDIBELL, TCGA and TIGER-LC) and in the iCCA animal models (Fig. 1a, b). The expression of *TGFB3* and *TGFBR2* was also increased in the animal models and in the TCGA cohort, while the *TGFBR3* expression remained stable or decreased, depending on the model (Fig. 1a, b). The increase in TGF-β ligands and receptors suggested activation of the TGF-β signalling in iCCA, that was confirmed by increased phosphorylation of SMAD2 in both human and murine iCCA tissues, compared to non-tumoral liver (Fig. 1c and Supplementary Fig. 2a). The activation of TGF-β signalling led to increased downstream gene expression, as indicated by the correlation of *SMAD7* expression—a typical SMAD-positively regulated gene—with *TGFB1* in human iCCA (Supplementary Fig. 2b) and the increased *Smad7* mRNA expression in tumours from the mouse iCCA models (Supplementary Fig. 2c). Importantly, high expression of TGF-β1 has been correlated with poor survival in iCCA patients.^20^ Accordingly, we found that high expression of *TGFBR1* and *TGFBR2* receptors also correlates with poor survival in a cohort of 246 iCCA patients (GSE244807) (Fig. 1d). Moreover, since genetic mutations in core components of the TGF-β signalling pathway, such as *SMAD4*,^21^ are very frequent in some tumours, we decided to rule out this mechanism of escape from TGF-β suppressor effects in iCCA. Analysis of the mutational status of the ligands (*TGFB1-3*), receptors (*TGFBR1-3*) and SMADs (*SMAD2-4*) in 1121 iCCA patients revealed a low mutation frequency in these genes, with driver mutations present in only 6.6% of patients (Fig. 1e). Altogether, these data demonstrate that TGF-β signalling is active and functional across species in iCCA and impacts iCCA patient survival.

### TGF-β inhibition promotes CCA tumour cell growth in vitro

**Fig. 2:**
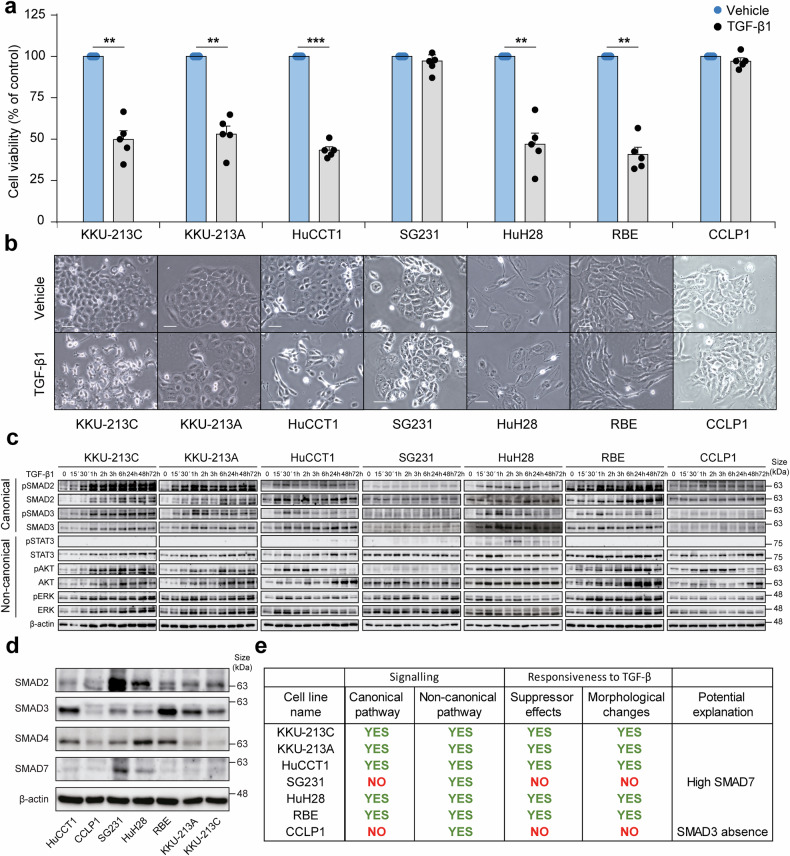
TGF-β1 effects on intrahepatic cholangiocarcinoma (iCCA) cell lines. **a** Cell viability of iCCA cell lines after exposure to TGF-β1 (2 ng/ml) for 72 h, as determined by cell counting. **b** Representative images showing the morphology of the iCCA cell lines at the time of cell viability determination. Scale: 250 µm. **c** Representative images of Western blot analysis of phosphorylation and total expression of SMAD2, SMAD3, STAT3, AKT and ERK1/2 in iCCA cell lines exposed to TGF-β1 (2 ng/ml) for the indicated times. **d** Representative images of Western blot analysis showing the basal protein expression of SMAD2, SMAD3, SMAD4 and SMAD7 in iCCA cell lines. **e** Summary of responses in a panel of 7 iCCA cell lines to TGF-β1 exposure based on the results displayed in panels **a**-**d**. Values are expressed as means ± SEM from at least 3 cultures. **p < 0.01, ***p < 0.001 as compared to the vehicle

The dual effect of TGF-β in tumour cells has been extensively studied in different cancers, including HCC. TGF-β is able to repress proliferation and induce apoptosis in HCC cells that resemble earlier stages of tumour development, while cells resembling advanced stages are able to escape from TGF-β suppressor functions by different mechanisms and exploit its pro-tumorigenic properties, becoming more migratory and invasive.^22–24^ Since these processes remain largely understudied in iCCA, we performed in vitro studies to evaluate the effects of TGF-β1 on the viability and morphology of seven human iCCA cell lines (Fig. 2a, b). Five cell lines (KKU-213A, KKU-213C, HuCCT1, HuH28 and RBE) showed a significant reduction in cell viability below 50% and changes in morphology that could account for an epithelial-mesenchymal transition process, while two cell lines (CCLP1 and SG231) did not show changes in these parameters (Fig. 2a, b). We then analysed the activation of canonical and non-canonical intracellular signalling pathways by determining the phosphorylation levels of SMAD2 and SMAD3, and STAT3, AKT and ERK, respectively. This analysis revealed activation of non-canonical signalling (STAT3, AKT or ERK) pathways in all cell lines (Fig. 2c). Interestingly, only the five cell lines with reduced viability upon TGF-β1 treatment showed increased phosphorylation of canonical SMAD2/SMAD3, whereas the two cell lines that did not exhibit reduced viability did not display increased phosphorylation of either SMAD2 or SMAD3 (Fig. 2c). To explain the lack of TGF-β1 effects we analysed the expression of the main actors involved in the TGF-β canonical signalling pathway. All seven cell lines showed normal and detectable mRNA levels of the three TGF-β ligands and three TGF-β receptors (Supplementary Fig. 3). However, further analysis of SMAD protein expression showed that CCLP1 cells lacked SMAD3 expression, and SG231 cells expressed very high levels of SMAD7 (Fig. 2d), which acts as a negative regulator of TGF-β signalling,^25^ explaining why these two cell lines do not respond to TGF-β. In summary (Fig. 2e), TGF-β1 exerts a strong suppressive effect on iCCA cell lines that is dependent on a functional canonical SMAD signalling, which represents an important difference with HCC, where cells can maintain an active canonical TGF-β signalling and still not respond to TGF-β suppressor effects. Further analysis of HuCCT1 and RBE cells, which respond to TGF-β1 in terms of viability suppression (Fig. 2a) and SMAD-dependent gene regulation (Supplementary Fig. 4), indicated that the suppressive effect of TGF-β1 occurs via reduced cell proliferation, as indicated by reduced Ki67 staining (Supplementary Fig. 5a). We also identified a reduction in cyclin D1 expression, suggesting a possible mechanism (Supplementary Fig. 5b). Finally, we observed an induction of apoptosis, indicated by increased cleavage of PARP (cPARP) (Supplementary Fig. 5c).

**Fig. 3:**
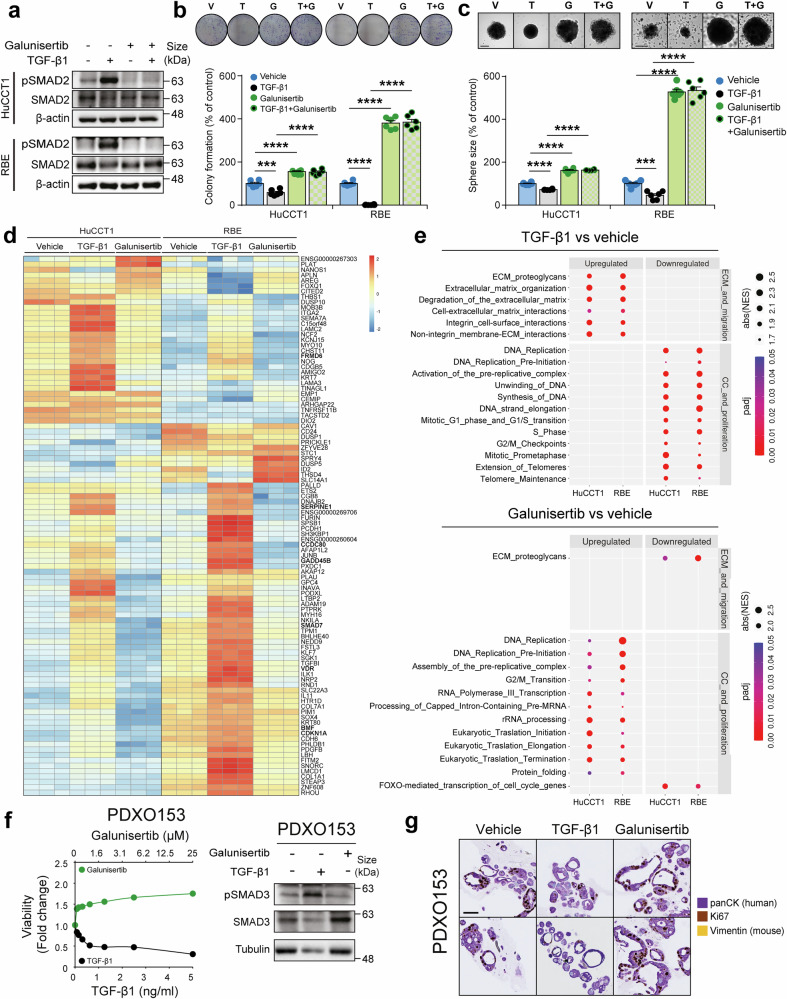
TGF-β receptor inhibition promotes intrahepatic cholangiocarcinoma (iCCA) tumour cell growth by inhibiting SMAD canonical signalling. **a** Western blot images showing SMAD2 phosphorylation in HuCCT1 and RBE cells treated with TGF-β1 (2 ng/ml), TGF-β1 receptor I inhibitor galunisertib (10 µM) or both, for 1 h. **b** Number of colonies of HuCCT1 and RBE cells after 7-10 days of treatment with TGF-β1 (2 ng/ml), galunisertib (10 µM) or both. Representative images of colonies are shown **c** Size of spheres from HuCCT1 and RBE cells after 4 days of treatment with TGF-β1, galunisertib (10 µM) or both. Representative images of spheres at the time of size analysis are shown. Scale: 100 µm. **d, e** RNA-seq analyses conducted in HuCCT1 and RBE cells treated with TGF-β1 (2 ng/ml) or TGF-β1 receptor I inhibitor galunisertib (10 µM). Heatmap (**d**) showing changes in the expression of different genes in the iCCA cell lines in response to TGF-β1 or galunisertib. Dot plot (**e**) showing differences in enrichment for key events related with “ECM (extracellular matrix) and migration” and “Cell cycle and proliferation” in iCCA cells. **f** Dose-response curves of TGF-β1 and galunisertib for PDXO153 organoids derived from CCA_PDXs (PDXO). Cell viability was determined using a Cell Titer-Glo assay 4 days after the treatment initiation. Representative images of Western blot analysis of phosphoSMAD3 and total SMAD3 in PDX153 organoids after treatment with TGF-β1 (2 ng/ml) or galunisertib (10 µM) for 4 days are shown. **g** Representative immunohistochemical images showing human panCK (labelling human iCCA tumour cells), murine vimentin (labelling stromal murine cells) and Ki67 (as a marker of cell proliferation) in PDXO153 organoids after the same treatment than in **f**. Scale: 200 µm. Values are expressed as means ± SEM from at least 3 cultures. **p < 0.01, ***p < 0.001, as compared to the vehicle. V vehicle, T TGF-β1, G galunisertib

The strong suppressor effect induced by TGF-β1 on iCCA cell lines led us to hypothesize that the use of TGF-β inhibitors, such as galunisertib (a selective TGF-β receptor type I kinase inhibitor), would produce the opposite effect. Indeed, galunisertib inhibited the phosphorylation of SMAD2 induced by TGF-β1 treatment in HuCCT1 and RBE, and also reduced the basal levels of SMAD2 phosphorylation (Fig. 3a), indicating that it is able to impair the autocrine signalling of the TGF-β produced by iCCA cells. We therefore conducted 2D and 3D in vitro functional studies to validate this point. Colony and spheroid formation assays demonstrated that TGF-β1 strongly reduced and galunisertib significantly increased the number of colonies and the size of the spheres formed by HuCCT1 and RBE cells (Fig. 3b, c). The combination of TGF-β1 and galunisertib showed similar effects than galunisertib alone indicating that this dose of galunisertib is enough to inhibit the autocrine TGF-β signalling in iCCA cell lines, as well as the exocrine TGF-β1 added to the cultures. Galunisertib is able to inhibit numerous other kinases at this dose.^26^ However, all these off targets are involved in increasing proliferation/survival of the cells and, thus, its inhibition would not explain the increase in the number of colonies and in the size of spheres (Fig. 3b, c), indicating that the effects observed are mediated by TGF-β signalling inhibition.

To better understand the actions of TGF-β signalling in iCCA, we compared the transcriptomic changes induced in iCCA cells after exposing them to TGF-β1 or galunisertib alone by RNA-seq analysis, showing that the top genes regulated were modulated in an opposite way by TGF-β1 and galunisertib, as expected (Fig. 3d). Gene set enrichment analysis (GSEA) further validated the effect of these compounds, showing upregulation and inhibition of signalling by TGF-β-receptor complex and SMADs in cells treated with TGF-β1 and galunisertib, respectively (Supplementary Fig. 6-7). Notably, *SMAD7* and *SERPINE1* were upregulated by TGF-β1 and downregulated by galunisertib (Fig. 3d). It was previously described, that the pro-tumorigenic effects of TGF-β are related to the increase of the migration capabilities of the cell and the modification of the ECM to render them more invasive,^8,27^ while its suppressor effects are related to the regulation of cell cycle and proliferation.^8^ In line with this, our GSEA analysis revealed that TGF-β1 upregulated pathways related to ECM modulation and migration (i.e. interactions with surface integrins), while it downregulated pathways involved in DNA synthesis and cell cycle progression (Fig. 3e and Supplementary Figs. 8–10), consistent with TGF-β suppressor effects. Intriguingly, the effects of galunisertib on ECM and migration were much less prominent, but its effects were concentrated in upregulating pathways related to cell cycle progression and DNA synthesis (Fig. 3e and Supplementary Figs. 8–10). When analysing specific genes, we observed upregulation by TGF-β and downregulation by galunisertib of typical TGF-β target genes related to its suppressor activity (*BMF*, *CDKN1A* and *GADD45B*). Additionally, we also identified other genes with the same profile (Fig. 3d and Supplementary Fig. 11a) that are not TGF-β known targets but would be of interest to be explored in the future as there are reports showing a potential suppressor role in other tumours (*CCDC80*^28^, *FRMD6*^29^ and *VDR*^30^). Interestingly, the expression of some of these genes (*BMF*, *CDKN1A*, *GADD45B* and *CCDC80*) changed accordingly in the spheroid model (Supplementary Fig. 11b).

We wanted to further validate the effect of TGF-β1 and galunisertib in organoids derived from PDXs (PDXO) established from iCCA metastases biopsies, which better recapitulate tumour complexity. Three of the four organoid models tested showed a strong decrease in viability by TGF-β1 and a significant increase by galunisertib (Fig. 3f and Supplementary Fig. 12). In line with our previous observations, the only organoid showing no changes in viability lacked a functional canonical TGF-β signalling with no SMAD3 phosphorylation, corroborating that TGF-β signalling inhibition promotes tumour cell growth only when SMAD canonical signalling is properly functional. To further validate the suppressor role of TGF-β signalling in the organoids, we conducted co-immunohistochemical studies of panCK and Ki67 to specifically label tumour proliferating cells. As shown in Fig. 3g, TGF-β reduced both organoid size and tumour cell proliferation, while galunisertib increased both of them. Lack of murine vimentin staining confirmed the absence of murine cells and the purity of our human organoids.

Altogether, our results show that TGF-β impairs cell growth in all iCCA cell lines with intact TGF-β signalling, and not in those with impaired signalling. Therefore, TGF-β role over the tumour cells is prominently suppressor in iCCA and, thus, the use of inhibitors targeting the TGF-β receptor may present counterproductive effects by inducing tumour cell proliferation.

### NOX4 is overexpressed in iCCA and it is restricted to CAF

Facing the above-described evidence, we decided to follow a different strategy and investigate downstream mediators of the TGF-β signalling pathway that would allow us to target exclusively TGF-β pro-tumorigenic actions. Among the potential candidates, we focused our attention on NOX4 because it has been implicated in mediating TGF-β pro-tumorigenic effects on the TME, more specifically in CAF activation in several tumours.^15^ Furthermore, NOX4 inhibitors exist, are commercially available and are being tested at the clinical level in different settings. This potential, together with the lack of information about the role and therapeutic potential of NOX4 in iCCA tumour cells and TME, makes NOX4 the ideal candidate.

**Fig. 4:**
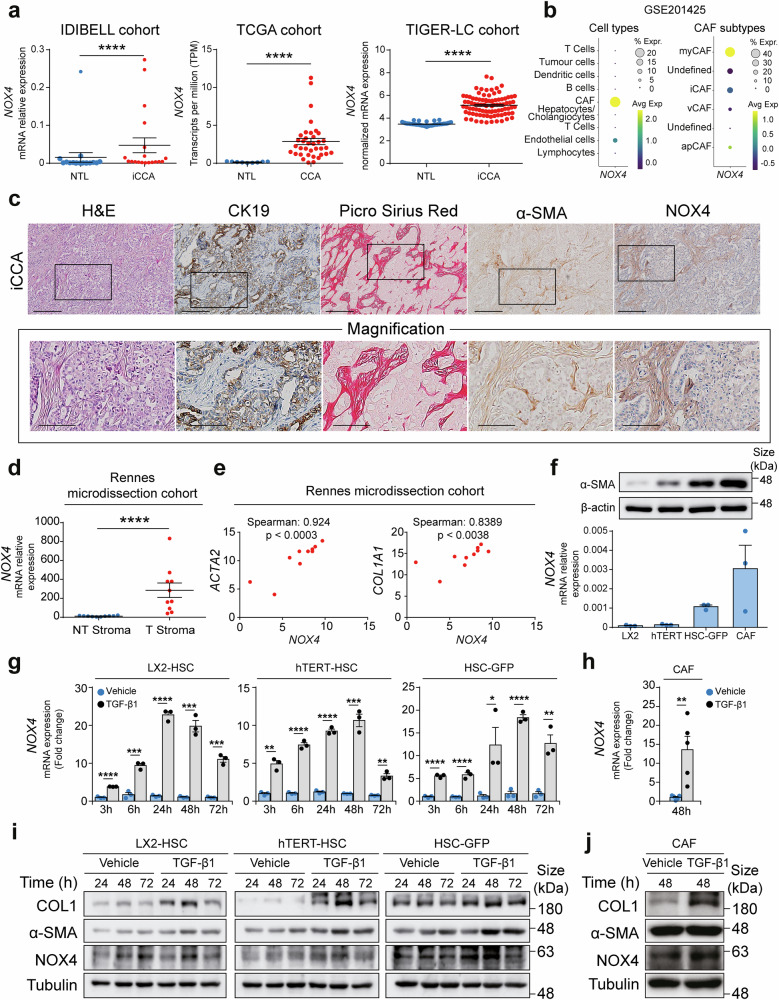
NOX4 is overexpressed in intrahepatic cholangiocarcinoma (iCCA) and specifically expressed in cancer-associated fibroblasts (CAF). **a** *NOX4* mRNA expression in CCA tumours compared to surrounding liver tissue (NTL) from the IDIBELL (*n* = 17), the TCGA (*n* = 36) and TIGER-LC (*n* = 91) cohorts of patients. **b** Dot plot showing *NOX4* expression in cell types and cancer-associated fibroblasts (CAF) subtypes (apCAF antigen-presenting CAF, iCAF inflammatory CAF, myCAF myofibroblast CAF vCAF vascular CAF) from scRNAseq data set GSE201425 from CCA biopsies. **c** Representative immunohistochemistry images from human iCCA showing tumour architecture (hematoxylin-eosin, H&E), tumour cells (CK19), extracellular matrix (Picro Sirius Red) produced by CAF (α-SMA), and NOX4 localization. Scale: 200 µm (100 µm in magnification pictures). **d** *NOX4* mRNA expression in non-tumoral (NT) or tumoral (T) stromal samples from the Rennes microdissection cohort. **e** Correlation between *ACTA2* and *COL1A1* with *NOX4* expression in microdissected stroma from 10 iCCA samples. **f** *NOX4* mRNA and α-SMA protein basal expression in LX2-HSC, hTERT-HSC, HSC-GFP cells and CAF, as determined by RT-QPCR and Western blot, respectively. **g**, **h** *NOX4* mRNA expression in LX2-HSC, hTERT-HSC and HSC-GFP cells (**g**) and CAF (**h**) after exposure to TGF-β1 (2 ng/ml) for the indicated times. Values are expressed as fold change *versus* vehicle (3 h time point in **g**) and represented as means ± SEM from at least 3 cultures. **i, j** Representative images of Western blot analysis of Collagen 1 (COL1), α-SMA and NOX4 protein expression in LX2-HSC, hTERT-HSC and HSC-GFP cells (**i**) and CAF (**j**) after exposure to TGF-β1 (2 ng/ml) for the indicated times. mRNA expression levels were determined by RT-qPCR in samples from the IDIBELL cohort of patients. Gene expression data from public data bases was obtained using RNAseq (TCGA cohort) or microarrays (TIGER cohort). *p < 0.05, **p < 0.01, ***p < 0.001, ****p < 0.0001; as compared to NTL or the vehicle. Correlations were determined using Spearman’s correlation analysis

Analysis of the human datasets showed increased *NOX4* expression in human iCCA *versus* surrounding non-tumorous liver (Fig. 4a). Interestingly, analysis of scRNAseq data demonstrated that *NOX4* expression was restricted primarily to CAF (Fig. 4b and Supplementary Fig. 13). Accordingly, the expression of *NOX4* correlated with the expression of CAF markers in iCCA patients (Supplementary Fig. 14). Further evaluation of scRNAseq data allowed us to define the four major CAF subpopulations (myCAF, iCAF, vCAF and apCAF) and to determine that *NOX4* was mainly expressed in myCAF and, to a lesser extent, in apCAF (Fig. 4b and Supplementary Fig. 15). To confirm the expression of NOX4 in CAF from iCCA we carried out immunohistochemical analysis and staining for different markers of tumour cells and CAF. For the staining of NOX4 we used an antibody previously manufactured and validated for IHC.^31^ Indeed, our analysis revealed that NOX4 shows an intense and specific localization in stromal areas of iCCA where α-SMA and Picro Sirius red signals are also strong, indicating NOX4 expression in CAF, while no expression was found in CCA tumour cells (Fig. 4c). Furthermore, analysis of data from laser-microdissected iCCA stromal samples revealed an increase in NOX4 expression in tumour stroma *versus* non-tumoral stroma (Fig. 4d) and a correlation of *NOX4* expression with that of typical markers of CAF (*ACTA2* and *COL1A1*) (Fig. 4e), suggesting that *NOX4* expression may play a role in the activation of CAF during carcinogenesis. Indeed, *NOX4* expression was absent in iCCA cell lines, compared to non-tumoral liver or HCC cell lines (Supplementary Fig. 16), while it was present in LX2-HSC and hTERT-HSC cell lines, primary HSC-GFP and CAF from iCCA, correlating with the degree of activation as indicated by the levels of α-SMA (Fig. 4f). Exposure of these fibroblastic cells to TGF-β1 to stimulate their activation showed an increased expression of *NOX4* compared to the vehicle, concomitant with an increased expression of α-SMA (*ACTA2*) and Collagen 1 (*COL1A1*) (Fig. 4g–j and Supplementary Fig. 17a, b). Interestingly, in HSC cells the mRNA expression of *NOX4* increased at earlier times (from 3 h to 72 h) (Fig. 4g), compared to that of *ACTA2* and *COL1A1* (starting from 24 h) (Supplementary Fig. 17 a-b), suggesting that NOX4 may be indeed a downstream mediator of TGF-β induced fibroblast transdifferentiation, as it has been described in other tumours.^15^

### Dual NOX4 and NOX1 depletion is necessary to impair human liver fibroblast transdifferentiation

**Fig. 5:**
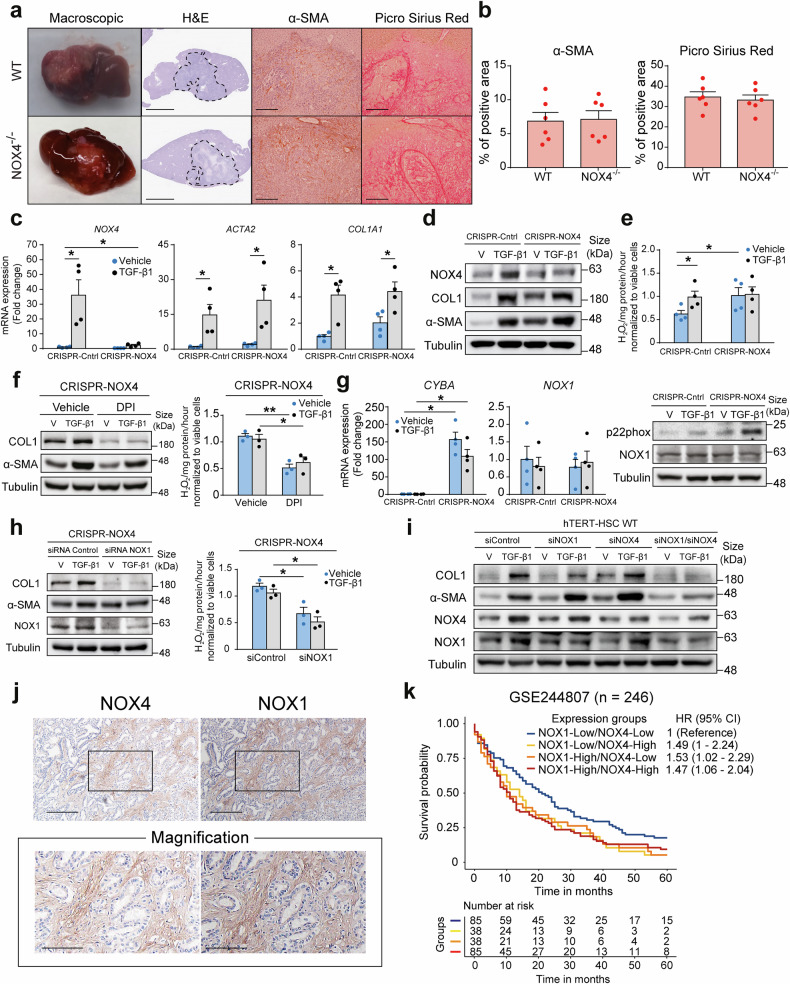
NOX4 and NOX1 act in concert to allow TGF-β-induced fibroblast transdifferentiation. Analyses were conducted on the syngeneic SB1 orthotopic iCCA tumours in WT and NOX4^-/-^ mice (*n* = 6), established as illustrated in Supplementary Fig. 18. **a** Representative macroscopic images of the tumours at term and tissue sections stained with hematoxylin-eosin (H&E), to illustrate the size of the tumours, α-SMA to visualize cancer-associated fibroblasts (CAF) and Picro Sirius Red to visualize collagen deposition. Scale (H&E): 2.5 mm. Scale (α-SMA and Picro Sirius Red): 250 µm. **b** α-SMA and Picro Sirius Red positive area was quantified using ImageJ. **c**, **d** mRNA (**c**) and protein (**d**) expression of *NOX4*, *ACTA2* (α-SMA) and *COL1A1* (Collagen 1, COL1) after TGF-β1 treatment for 48 h in the hTERT-HSC CRISPR-Control and CRISPR-NOX4 cells, determined by RT-qPCR and Western blot. **e** H_2_O_2_ production was measured by Amplex red in CRISPR-Control and CRISPR-NOX4 cells after TGF-β1 treatment for 24 h and expressed as the amount of H_2_O_2_ normalized by mg of protein per hour. **f** Protein expression of α-SMA and COL1A1 and H_2_O_2_ production (as determined in **d**, **e**) in CRISPR-NOX4 cells treated with TGF-β1 in absence or presence of the NADPH oxidase inhibitor DPI (2.5 µM). **g** mRNA and protein expression of *CYBA* (p22phox) and NOX1 after TGF-β1 treatment for 48 h in the CRISPR-NOX4 and CRISPR-control cells, determined by RT-QPCR and Western blot respectively. **h** Protein expression of NOX1, α-SMA and COL1A1 and H_2_O_2_ production (as determined in **d**, **e**) in CRISPR-NOX4 cells treated with TGF-β1 and transfected with siRNA against *NOX1* or the scrambled version (siControl). **i** Protein expression of α-SMA, COL1, NOX4 and NOX1 in hTERT-HSC WT cells transfected with siRNA against *NOX4*, *NOX1* or both, and treated with TGF-β1. **j** Immunohistochemical analysis of NOX4 and NOX1 localization in serial tissue sections from human iCCA samples (IDIBELL cohort) (representative images). Scale: 200 µm. **K** Kaplan-Meier curve of 5-year overall survival of patients from GSE244807 cohort (*n* = 246) stratified based on *NOX4* and *NOX1* expression (Cox Proportional Hazards model). mRNA expression levels are expressed as fold change versus vehicle. Values are expressed as means ± SEM from at least 3 cultures. *p < 0.05; **p < 0.01

To evaluate the potential impact of targeting NOX4 in iCCA we performed a syngeneic orthotopic model by implanting the SB1 cells in WT and NOX4^-/-^ mice (Supplementary Fig. 18). Surprisingly, despite the absence of NOX4 in stromal cells, tumours from WT and NOX4^-/-^ mice were identical in size and in the expression levels of *Krt19*, α-Sma (*Acta2*) and collagen 1 (*Col1a1*) (Fig. 5a, b and Supplementary Fig. 19a). To determine the role of NOX4 in the transdifferentiation of liver fibroblasts and to explore the potential reasons for the absence of differences observed in NOX4^-/-^ mice in vivo, we opted to model the depletion of NOX4 in vitro using the CRISPR-Cas9 strategy. Reduced *NOX4* mRNA and protein expression in hTERT-HSC cells was observed (Fig. 5c, d), but once again, both CRISPR-Control and CRISPR-NOX4 cells showed similar responses to TGF-β1 with an increase in the expression of both α-SMA (*ACTA2*) and Collagen 1 (*COL1A1*) (Fig. 5c, d). Interestingly, TGF-β1 was able to increase ROS levels in CRISPR-Control cells, while basal ROS levels were already elevated in CRISPR-NOX4 cells and did not change after exposure to TGF-β1 (Fig. 5e). This result led us to think that there could be a compensatory mechanism increasing ROS levels enough to allow TGF-β1-induced fibroblast transdifferentiation. To ascertain if another NADPH oxidase was involved, we treated CRISPR-NOX4 cells with diphenyleneiodonium chloride (DPI), a specific NADPH oxidase inhibitor widely used as a reference compound in NOX research. In these cells, DPI abolished TGF-β1 induced increase of α-SMA and Collagen 1 (Fig. 5f) and reduced ROS levels, indicating that another NADPH oxidase was involved. Further analysis of the expression of other NADPH oxidases and the factors forming their regulatory complexes showed a strong increase in the expression of p22phox (*CYBA*) and a stable and detectable expression of *NOX1* (Fig. 5g), while no other NOX was detectable and the rest of regulatory components did not suffer a change in expression that could account for the increased ROS levels (Supplementary Fig. 19b). Notably, NOX1 expression was also detectable at the mRNA and protein level in human CAF from CCA (Supplementary Fig. 19c). Since NOX4 is constitutively active and it is regulated by modulating its expression, while NOX1 expression is mostly stable but its activity is regulated by other partners from its complex, we hypothesized that the increase in p22phox resulted from NOX4 knock-down could be able to further increase the activity of NOX1. To investigate this possibility, we attempted to generate double NOX4/NOX1 CRISPR cells, but all our attempts were unsuccessful, probably because total depletion of both NOX4 and NOX1 is too deleterious for the fibroblasts. Thus, we proceeded to perform transient downregulation of *NOX1* by siRNA transfection. Indeed, siRNA downregulation of *NOX1* in CRISPR-NOX4 cells clearly downregulated NOX1 protein levels, abolished TGF-β1-induced increase of α-SMA and Collagen 1, and reduced ROS levels (Fig. 5h), validating this compensatory mechanism. To further elucidate the specific contribution of NOX4 and/or NOX1 to TGF-β-induced fibroblast transdifferentiation, we performed additional siRNA studies in WT cells. Notably, only the combined downregulation of *NOX4* and *NOX1* was able to impair TGF-β1-induced increase in α-SMA and Collagen 1 in both hTERT-HSC and LX2-HSC cells (Fig. 5i and Supplementary Fig. 20a–c). Immunohistochemical studies with a validated NOX1 antibody^32^ in serial sections from human iCCA confirmed NOX4 and NOX1 protein expression in the same stromal cells (Fig. 5j). Interestingly, our findings correlate with patient survival, since only patients with low expression of both *NOX4* and *NOX1* show better survival than those with high expression of one or both *NOXs* (cohort GSE244807) (Fig. 5k), indicating the important role played by the duet NOX4/NOX1 in iCCA pathobiology.

### Dual NOX4/NOX1 inhibition with setanaxib impairs HSC and CAF functions and reduces iCCA growth in vitro

**Fig. 6:**
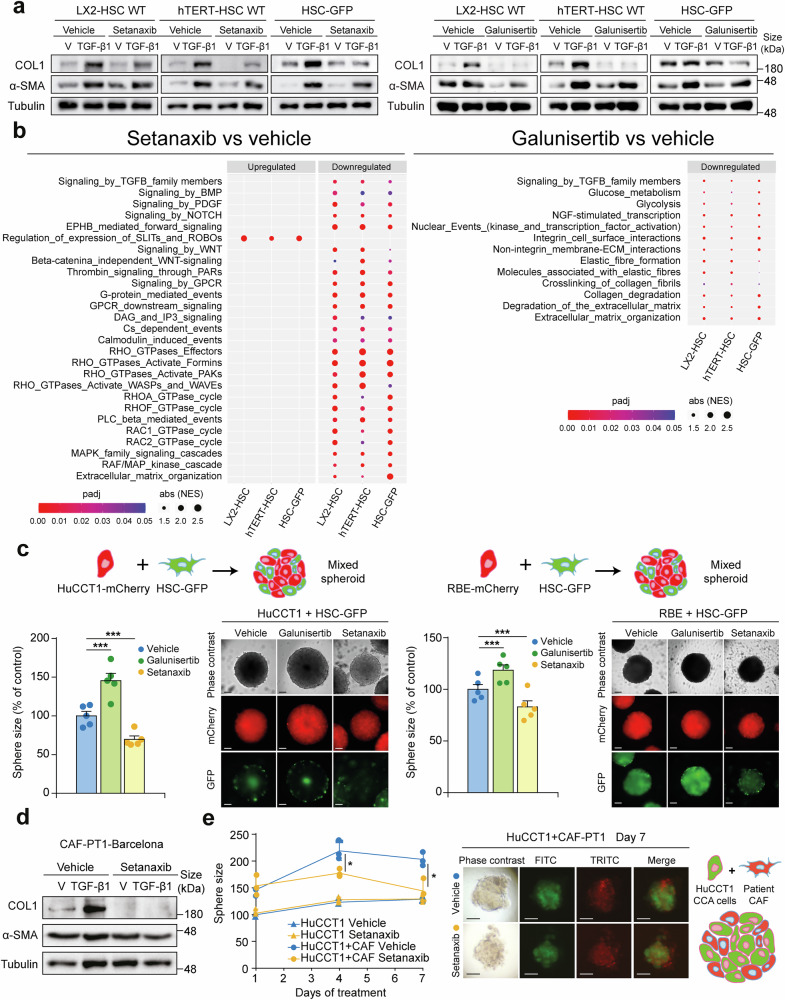
Dual NOX4/NOX1 inhibition impairs TGF-β-induced fibroblast transdifferentiation and cancer-associated fibroblasts (CAF) functions. **a** Representative images of Western blot analysis of Collagen 1 (COL1) and α-SMA in hepatic stellate cell (HSC) lines treated with TGF-β1 or the vehicle in absence or presence of setanaxib (left) or galunisertib (right) for 48 hours. **b** Dot plot showing differences in enrichment for key events related with signalling pathways involved in myofibroblast transdifferentiation in HSC lines treated with setanaxib or galunisertib. **c** Size of mixed spheres from HuCCT1 or RBE cells tagged with mCherry in combination with HSC-GFP after 4 days of treatment with setanaxib or galunisertib. Representative phase contrast images of spheres at the time of size analysis are shown. Also, fluorescence microscopy images of mCherry and GFP intensity are shown. Scale: 100 µm. **d** Representative images of Western blot analysis of COL1 and α-SMA in CAF treated with TGF-β1 (2 ng/ml) or the vehicle in absence or presence of setanaxib (40 µM) for 48 hours. **e** Size of spheres from HuCCT1 (marked in green with FITC) alone or in combination with CAF (marked in red with TRITC) after 1, 4 or 7 days of treatment with setanaxib or the vehicle. Representative phase contrast and images of spheres at the time of size analysis are shown. Also, fluorescence microscopy images of FITC and TRITC are shown. Scale: 50 µm. Values are expressed as means ± SEM from at least 3 cultures. *p < 0.05, **p < 0.01; ***p < 0.001; as compared with the vehicle

The necessity of impairing the activity of both NOXs to blunt fibroblast transdifferentiation led us to evaluate the activity of a new inhibitor. Despite DPI specificity in molecular experimental settings, its therapeutic development has not advanced. Thus, as a potential therapeutic tool, we decided to use setanaxib, a dual NOX4/NOX1 inhibitor that has been successfully tested in clinical trials of cholangiopathies (NCT03226067).^33^ For this purpose, we used LX2-HSC, hTERT-HSC and HSC-GFP that show different degrees of basal activation and we further stimulated them with TGF-β1 in the absence or presence of setanaxib and galunisertib, the latter as positive control for TGF-β1 signalling inhibition. Both setanaxib and galunisertib were able to impair TGF-β1-induced increment in the expression of both α-SMA and Collagen 1 (Fig. 6a). Interestingly, both drugs showed the ability to inhibit the expression of these proteins in the absence of TGF-β1, especially that of Collagen 1 (Fig. 6a). Next, we decided to evaluate the transcriptomic effects of both setanaxib and galunisertib in HSC by RNAseq (Supplementary Fig. 21a). Interestingly, RNAseq analysis showed that setanaxib was able to regulate a higher number of genes than galunisertib (1390 vs 325) (Supplementary Fig. 21b, c and Supplementary Fig. 22-23). GSEA analysis showed that setanaxib was able to inhibit many signalling pathways involved in myofibroblasts activation, as well as ECM organization, while galunisertib mostly regulated pathways related to ECM metabolism and interaction with integrins (Fig. 6b and Supplementary Figs. 24-26). Interestingly, when we analysed the relative enrichment in the myCAF signature in the HSC cell lines treated with the inhibitors, we observed that both setanaxib and galunisertib were able to reduce it, compared with untreated cells, especially in the case of hTERT-HSC and HSC-GFP cells, indicating the potential of these inhibitors to inhibit the myCAF phenotype (Supplementary Fig. 27). Since both galunisertib and setanaxib could inhibit fibroblast functions, we decided to test their impact on CCA growth using mixed spheroids formed by malignant cells and HSC-GFP. To follow the dynamics of the experiment we labelled the tumour cells with mCherry and compared it with the GFP of HSC cells. Setanaxib had no impact on the size of spheroids formed by tumour cells alone or in combination with TMNK1 endothelial cells or THP-1 macrophages (Supplementary Fig. 28) but significantly reduced the growth of mixed spheroids (Fig. 6c), demonstrating the specificity of setanaxib on liver myofibroblasts, the only NOX4/NOX1 expressing cells. However, galunisertib increased the size of mixed spheroids (Fig. 6c), indicating that, despite being able to inhibit fibroblast functions, galunisertib effects on promoting tumour cell growth are predominant. Interestingly, green fluorescence from HSC-GFP decreased after treatment with setanaxib, which may be due to the inhibition of NOX1 previously described function on fibroblast proliferation.^34^ The inactivation of fibroblasts together with reduced proliferation may impact the crosstalk of tumour cells and fibroblast since both spheroid size and red fluorescence from tumour cells are reduced after setanaxib treatment (Fig. 6c). Next, we validated the efficacy of setanaxib on CAF from iCCA patients. Indeed, setanaxib was able to reduce the protein levels of CAF markers both in absence and presence of TGF-β1, especially that of Collagen 1 in three different sets of CAF (Fig. 6d and Supplementary Fig. 29). Finally, setanaxib was also able to reduce tumour growth in mixed spheroids formed by iCCA cells and CAF, while it did not have any effect on spheroids formed solely by tumour cells (Fig. 6e). In summary, these data suggest that setanaxib may be a promising strategy to target tumour growth by targeting CAF functions.

### Dual NOX4/NOX1 inhibition with setanaxib but not TGF-β receptor inhibition with galunisertib reduces iCCA tumour burden in vivo

Based on our in vitro studies, we hypothesised that using TGF-β inhibitors as therapy would be counterproductive due to the opposite effects of galunisertib on tumour cells (increase in tumour cell growth) versus TME cells (inhibition of CAF functions), while the use of NOX4/NOX1 inhibitors, such as setanaxib, would reduce tumour growth by specifically targeting TGF-β pro-tumorigenic actions in both newly recruited HSC and CAF. Thus, as a final step, we decided to compare the effects of galunisertib and setanaxib in vivo. For this purpose we followed two strategies: 1/we conducted an experiment in a PDX model to confirm the deleterious effect of galunisertib in a more humanized model and to evaluate the potential of setanaxib in inhibiting fibroblasts from origins other than the liver; 2/ we carried out an experiment in the AKT-YAP model to evaluate the effects of both drugs in a state-of-the-art iCCA murine model, that shows a prominent desmoplastic stroma with strong collagen deposition and is performed in immunocompetent mice.

**Fig. 7:**
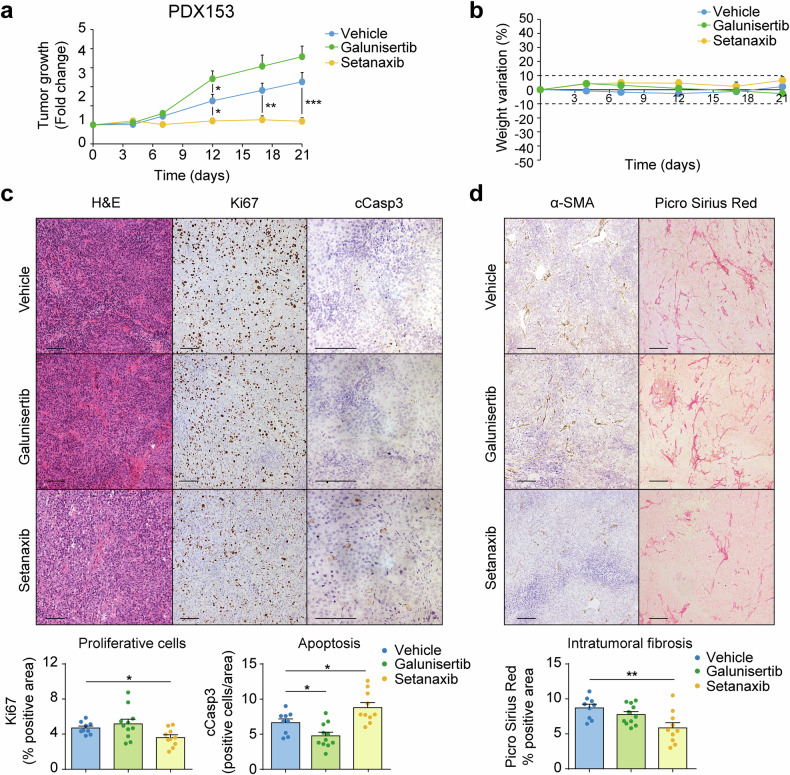
Therapeutic effect of dual NOX4/NOX1 inhibition with setanaxib on tumour progression in an intrahepatic cholangiocarcinoma (iCCA) PDX model. Tumour fragments from a previously characterized PDX (PDX153) were subcutaneously reimplanted into 6-week-old female NOD.CB-17-Prkdc scid/Rj. Once tumours reached an approximate volume of 150 mm^3^, mice started to be treated with galunisertib (150 mg/kg) or setanaxib (60 mg/kg). **a** Tumour growth was evaluated over 21 days in mice receiving vehicle, setanaxib or galunisertib (*n* = 6-7 animals per group). **b** Animals body weight was monitored through the experimental procedure time span to rule out potential toxic effects of any of the drugs. **c, d** Representative images of hematoxylin-eosin (H&E) and immunohistochemistry for Ki67 and cleaved-Caspase 3 (cCap3) (**c**). Immunohistochemistry for α-SMA and Picro Sirius Red (**d**). Scale: 100 µm. Quantification of Ki67, cCasp3 and Picro Red Sirius positive areas are shown below. Values are expressed as means ± SEM. *p < 0.05; **p < 0.01; ***p < 0.001; as compared with untreated mice

For the PDX model, we chose PDX153, from which PDXO153, used in our in vitro assays (Fig. 3f), was derived. After implantation, we waited until the tumours reached a mean size of 150 mm^3^ and then we started the treatments that continued for three weeks (Supplementary Fig. 30a). As shown in Fig. 7a and Supplementary Fig. 30b, galunisertib increased the growth of the tumours compared with the vehicle, while setanaxib strongly suppressed tumour growth. Importantly, both drugs were safe and did not produce any significant changes in the weight of the animals (Fig. 7b). To further evaluate the effects of galunisertib and setanaxib on the tumours we performed immunohistochemical stainings of different markers. First, we evaluated the growth and survival of cells inside the tumour by staining of Ki67 and cleaved caspase 3. In agreement with the tumour growth, setanaxib was able to reduce cell proliferation and increase apoptosis, while galunisertib only decreased apoptosis, but had no effect on cell proliferation (Fig. 7c). Then, we evaluated the changes in the fibrotic stroma of the tumours by analysing α-SMA and Picro Sirius Red staining. As expected, setanaxib strongly reduced the amount of CAF and the collagen deposition compared with the vehicle, while galunisertib did not show significant changes (Fig. 7d).

**Fig. 8:**
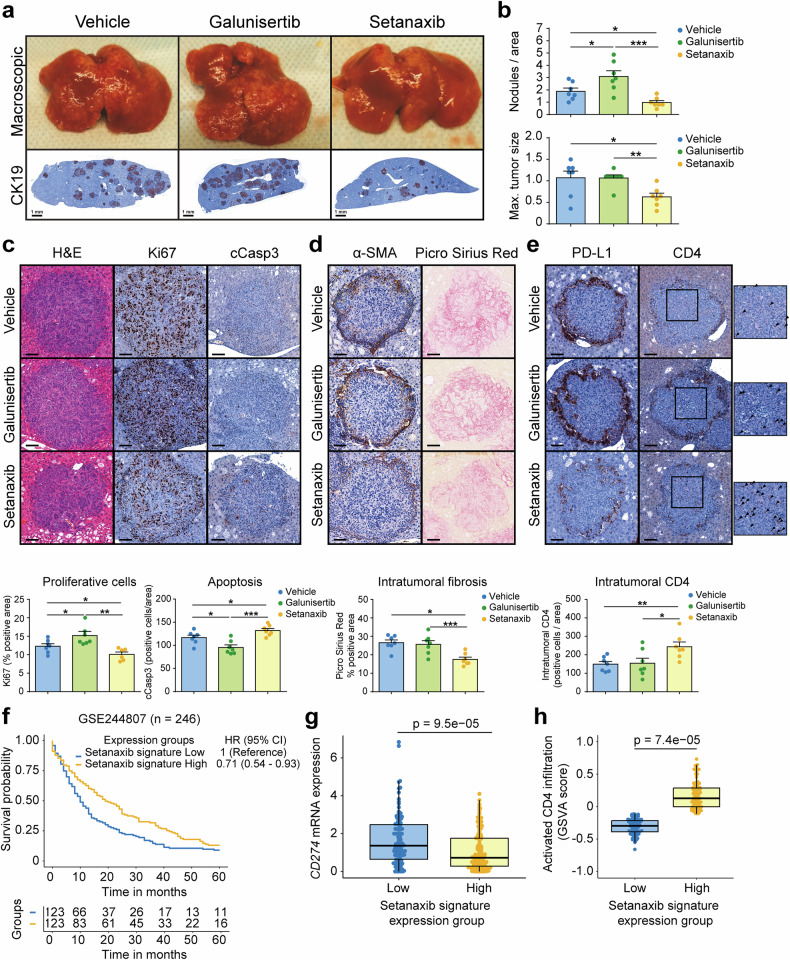
Dual NOX4/NOX1 inhibition with setanaxib reduces tumour burden in vivo in the AKT/YAP mouse intrahepatic cholangiocarcinoma (iCCA) model. iCCA tumours were induced using the HTVI AKT-YAP model and treated with galunisertib (150 mg/kg) or setanaxib (60 mg/kg) as illustrated in Supplementary Fig. 31a. **a** Representative macroscopic images of livers and Cytokeratin-19 (CK19) immunohistochemistry (to mark CCA cells) from animals treated with galunisertib or the vehicle. Scale: 1 mm. **b** Quantification of number of nodules/area and maximal tumour size in livers from **a**. **c**–**e** Representative images (left) of hematoxylin-eosin (H&E) and IHCs for Ki67 and cleaved-Caspase 3 (cCap3) (**c**), IHC for α-SMA and Picro Sirius Red (**d**) and IHCs for PD-L1 and CD4 (**e**) in liver sections from mice treated with vehicle, galunisertib and setanaxib. Scale: 100 µm. Quantification of Red Sirius and Ki67 positive areas and cCasp3, PD-L1 and CD4 positive cells are shown below. Magnifications of CD4 intratumoural staining are shown. Arrows indicate CD4 positive cells. Values are expressed as means ± SEM from 7 animals. *p < 0.05; **p < 0.01; ***p < 0.001; ****p < 0.0001; as compared with untreated mice. **f** Kaplan-Meier curve of 5-year overall survival of patients from GSE244807 cohort (*n* = 246) stratified based on enrichment of the setanaxib gene signature showed in Supplementary Fig. 22 (Cox Proportional Hazards model). **g** *CD274*/PDL1 expression in patients from GSE244807 cohort stratified as in **f**. **h** Activated CD4 infiltration in patients from GSE244807 cohort stratified as in **f**

After the promising results from the PDX model we proceeded to perform a similar experiment in the AKT-YAP iCCA model. In this case, we induced tumorigenesis and four weeks later, once the tumours were established, we treated the animals for two additional weeks before sacrifice (Supplementary Fig. 31a). Despite not being sacrificed at endpoint, when livers reached their peak in weight in this animal model,^35^ liver to body weight ratio in the setanaxib group showed a reduction compared to vehicle and galunisertib (Supplementary Fig. 31b). Indeed, analysis of the livers showed a reduced number of tumours in the setanaxib group. In contrast, galunisertib had the opposite effect and increased the number of tumour nodules, corroborating our in vitro results (Fig. 8a, b). Histopathological analysis of the tumours showed that vehicle and galunisertib-treated livers contained mostly undifferentiated iCCA with mainly trabecular growth within a desmoplastic stroma. Tumours showed expansive rather than infiltrative growth. On the contrary, setanaxib-treated livers showed smaller tumour nodules with pycnotic nuclei and the appearance of lower-grade lesions (Fig. 8c). Accordingly, quantification of IHC showed a reduced percentage of Ki67 positive cells and increased cleaved caspase 3 staining in setanaxib group, while the opposite effects were observed in galunisertib-treated tumours (Fig. 8c). Further staining of the TME showed similar disposition of CAF within the tumours in the three groups, although the staining was less intense in the setanaxib group. Accordingly, the setanaxib group showed reduced Picro Sirius Red staining compared to vehicle and galunisertib groups, indicating less collagen deposition by CAF (Fig. 8d). Therefore, altogether, these results corroborated our previous in vitro observations. Moreover, it is known that ECM deposition by CAF, including different collagens, increases tumour stiffness and forms a barrier that impedes T cell access into the interior of the tumours, negatively impacting the efficacy of current immunotherapies.^36^ In this sense, analysis of CD4-positive T cells showed that these cells are localised in the surroundings of iCCA tumours in this animal model. Interestingly, galunisertib increased the recruitment of CD4 T cells, but they remained excluded in the outer ring of the tumours, while setanaxib treatment increased the intratumoural presence of CD4 T cells (Fig. 8e). Furthermore, treatment with setanaxib also reduced the high expression of the immune checkpoint (ICP) PD-L1 in the tumour margins (Fig. 8e). No changes in other immune subpopulations or ICP analysed in this study were observed (Supplementary Fig. 31c). In an attempt to simulate the potential use of setanaxib in iCCA patients we stratified the patients from the GSE244807 cohort based on the enrichment in the gene signature obtained in the RNAseq analysis from Supplementary Fig. 22, corresponding with the genes commonly altered in the three HSC cell lines after exposure to setanaxib. Interestingly, patients enriched in setanaxib signature show a prolonged survival (Fig. 8f), reduced *CD274*/PD-L1 expression levels (Fig. 8g) and an increase in activated CD4 infiltration (Fig. 8h), accordingly with our results in mice treated with setanaxib (Fig. 8e).

Altogether, our results show that setanaxib allows targeting TGF-β pro-tumorigenic actions mediated by CAF, reducing tumour growth and modulating the TME, and represents a new therapeutic approach against iCCA.

## Discussion

TGF-β signalling plays a central role in carcinogenesis by modulating key events both in tumour cells and the TME.^8^ Therefore, to exploit the potential of TGF-β as a therapeutic target in iCCA, it is essential to understand the specific actions that TGF-β promotes in the different cells conforming the tumour. It has been widely reported that TGF-β can suppress or promote tumour progression depending on the stage of the disease. In general, TGF-β suppresses tumour growth at early stages, while acts as a tumour-promoting agent at later stages by inducing invasion and metastasis.^8^ To benefit from the tumour-promoting functions of TGF-β, tumour cells must decouple the TGF-β signalling from apoptosis while maintaining a functional TGF-β pathway.^8^ Our findings reveal important differences in the effects of TGF-β signalling activation in iCCA tumour cells compared to other cancers. In HCC, the most frequent liver cancer, tumour cells resembling later stages of tumour development can bypass TGF-β-induced apoptosis and cell cycle arrest and exploit TGF-β pro-tumorigenic functions while maintaining an active canonical TGF-β signalling.^22–24^ Our in vitro data shows that only iCCA cells with impaired SMAD2/3/4 signalling are able to escape TGF-β suppressor effects. On the contrary, cells with active SMAD2/3/4 undergo apoptosis and cell cycle arrest regardless of the stage of the tumour cell. We further validated this observation in organoids derived from metastatic iCCA samples, corresponding to the latest stage of tumour development. In other cancers, such as pancreatic and skin carcinomas, premalignant epithelial progenitors carrying mutations in *KRAS* or *HRAS* undergo apoptosis in response to TGF-β, and to escape this apoptosis, RAS-mutant cells acquire alterations that inactivate the TGF-β pathway or decouple TGF-β signalling from apoptosis.^37,38^ This characteristic also differs in iCCA. Indeed, our in vitro studies analysing proliferation arrest and apoptosis were performed in two cell lines (HuCCT1 and RBE) that carry mutations in *KRAS*.^39^

The recent approval of immunotherapy for the treatment of iCCA patients marked the first significant change in the standard of care for the majority of iCCA patients in decades. However, suboptimal responses in a substantial percentage of patients have directed strategies towards combining immunotherapy with inhibitors of other major signalling pathways exerting immunosuppressive actions. In this context, TGF-β appeared to be an ideal candidate, but the latest clinical trial using TGF-β inhibitors was discontinued because it did not meet its primary end-point of improving overall survival.^6^ The findings from our study may have important translational implications in this sense. Indeed, we demonstrated that TGF-β signalling inhibition using TGF-β receptor inhibitors promotes iCCA tumour cell growth. Even in mixed spheroids, the pro-proliferative effect of TGF-β inhibitors on iCCA cells prevails over the inhibitory effect on CAF, promoting spheroid growth. This, together with the low mutation rate in the members of the TGF-β canonical signalling pathway observed in iCCA patients, may counteract the positive effects of TGF-β inhibitors in boosting immunotherapies. Other tumours carrying a high percentage of mutations in key components of the canonical signalling, such as pancreatic cancer^40^ or extrahepatic CCA,^41^ in which *SMAD4* is one of the most mutated genes, could benefit from the use of these inhibitors. Nevertheless, further preclinical studies combining TGF-β inhibitors with immunotherapies in immunocompetent iCCA models must be conducted to clarify this issue.

To bypass the undesired effects of TGF-β inhibitors on tumour cells, we decided to switch strategies and search for downstream mediators specific to TGF-β pro-tumorigenic actions, focusing our attention on NOX4, which is highly increased in iCCA compared to normal tissue. The role of NOX4 in the tumour cell has been controversial, being defined as pro-tumorigenic in some tumours,^14^ and suppressor in others, including HCC.^13^ Interestingly, our data revealed that NOX4 expression is restricted to CAF from the TME in iCCA, which allows its therapeutical inhibition without undesired side effects on tumour cells. However, while the role of NOX4 in favouring TGF-β-induced fibroblast transdifferentiation has been undisputed over the years in different pathologies, including several cancers,^15,42–44^ a very recent study indicated that NOX4 is dispensable for skin myofibroblast differentiation and wound healing.^16^ In that report, NOX4^-/-^ mice did not display differences compared to WT mice in terms of wound healing, similar to the lack of impact of NOX4 invalidation on iCCA tumours in the SB1 syngeneic model found in our study. In agreement with this, primary fibroblasts isolated from mice or patients with NOX4 mutations were able to be activated after exposure to TGF-β, again in a similar way to our CRISPR-NOX4 hTERT cells. However, that report did not provide a mechanism by which fibroblasts can respond to TGF-β in the absence of NOX4, whereas we show here that NOX4 and NOX1 act in concert to allow TGF-β-induced transdifferentiation, at least in iCCA, and that dual inhibition is necessary to impair the aforementioned transdifferentiation. Thus, setanaxib could specifically target CAF functions and reduce iCCA growth both in vitro and in vivo. In this regard, our study presents some vulnerabilities. For instance, our inability to successfully produce a double NOX4/NOX1 CRISPR derived from hTERT-HSC prevented us from further validating this mechanism in a stable KO model. Whether the impossibility to generate this double CRISPR is due to technical reasons or to the lethality of depleting the expression of NOX4 and NOX1 simultaneously, is a question that remains unanswered. Another open question is why some studies found that depletion of NOX4 expression alone, either by shRNA techniques in vitro or in NOX4^-/-^ mice, was able to impact fibroblast activation in other tumour types, such as prostate,^45^ oesophageal^15^ or breast cancers.^46^ Perhaps the levels and/or function of NOX1 or the components of its regulatory complex (i.e. p22phox) are different in these organs and insufficient to trigger a compensatory mechanism after NOX4 depletion. Therefore, further investigation of these compensatory mechanisms is needed to untangle their true complexity.

Importantly, setanaxib has recently reached the clinical stage. In a clinical trial conducted in patients with primary biliary cholangitis (PBC) (NCT03226067), setanaxib showed evidence of potential anti-cholestatic and anti-fibrotic effects,^33^ which led to further investigation of setanaxib activity in a new clinical trial in PBC patients with elevated liver stiffness (NCT05014672). Furthermore, following the promising data demonstrating that setanaxib could overcome CD8 T exclusion in tumours from preclinical murine models,^42^ setanaxib is also being tested in combination with immunotherapy in a Phase II clinical trial in patients with recurrent or metastatic squamous cell carcinoma of the head and neck (NCT05323656). Interestingly, preliminary transcriptomic data from this trial showed that the two top pathways altered by setanaxib were the “Idiopathic Pulmonary Fibrosis Signalling Pathway” and the “Hepatic Fibrosis/Hepatic Stellate Cell Activation Pathway”, supporting the potential of setanaxib as an anti-fibrotic agent also in cancer. Indeed, we described here how setanaxib was able to reduce iCCA tumour growth as well as intratumoural fibrosis, correlating with the fact that NOX4 is mainly concentrated in myCAF, which produce ECM. Intriguingly, a proportion of NOX4 expression is found in apCAF, which have recently been involved in immunosuppression in pancreatic cancer.^47^ In this sense, we also observed a reduction of PD-L1 expression concomitant with lower α-SMA staining at the border of iCCA tumours in the setanaxib group compared to the vehicle and galunisertib groups. A recent report described that PD-L1 is expressed in HSC and is inducible by TGF-β.^48^ Furthermore, PD-L1 was able to control HSC activation by regulating TGF-β signalling, which impacted the ability of HSC to communicate with iCCA tumour cells via paracrine signalling, reducing tumour growth.^48^ This suggests that the reduction of PD-L1 expression by setanaxib may impact tumour growth independently of its role in the immune system regulation. Nevertheless, we also observed increased CD4 T cell infiltration in the setanaxib group, which is probably facilitated by the reduction of intratumoural fibrosis, as previously shown.^36^ Importantly, another recent study correlated intratumoural CD4 T cell infiltration with better survival in iCCA patients.^49^ In this regard, our simulation of iCCA stratification based on the setanaxib signature obtained in vitro in HSC rendered similar results: reduced PD-L1 expression, increased CD4 infiltration and an improved survival in iCCA patients. Nevertheless, further studies are needed to better characterise the importance of this infiltration and the potential impact of the different subpopulations of CD4^+^ T cells, which have been shown to play diverse roles and exert different effects on patient survival.^50^ Indeed, in light of our results and the fact that immunotherapy is now the current standard of care for iCCA patients,^3–5^ the next logical step in our investigations is to continue investigating the effects of setanaxib in combination with ICP inhibitors. However, given the reduction we observed in PD-L1 levels after treatment with setanaxib in the AKT-YAP model, anti-PD-L1 or anti-PD1 therapies may not be the best options, and a complementary analysis of the expression of other ICP in our model may be necessary to select the optimal therapy. In addition, it is important to consider that in combination with ICP, all iCCA patients still receive the chemotherapeutics gemcitabine and cisplatin, which may need to be added to the mixture in further experiments. Such an experiment, coupled with next-generation techniques of spatial transcriptomics could provide a more profound understanding of the dynamics of the TME under the effects of setanaxib and current iCCA therapies. If results are positive, this may lead to new clinical trials of setanaxib in iCCA patients. Altogether, the present evidence suggests a potentially bright future for dual NOX4/NOX1 inhibitors in clinics settings.

**Fig. 9:**
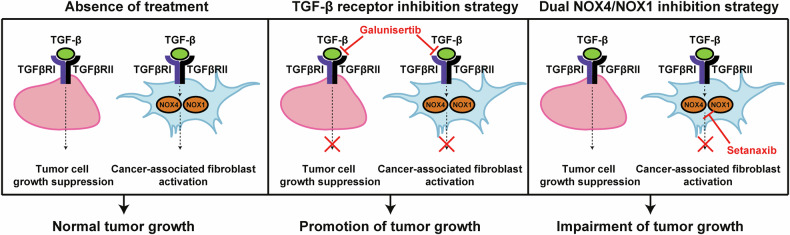
Impact of different strategies for TGF-β signalling inhibition on intrahepatic cholangiocarcinoma (iCCA) growth. In iCCA, TGF-β exerts suppressor effects on the tumoral cells but promotes cancer-associated fibroblasts (CAF) activation, contributing to the normal tumour growth (left panel). Inhibition of TGF-β signalling at the receptors level blunts TGF-β actions in both cell types, overall resulting in a promotion of tumour growth (middle panel). Inhibiting TGF-β signalling downstream using NOX4/NOX1 dual inhibitors blocks TGF-β mediated CAF activation while maintaining its suppressor effects on the tumour cells, therefore impairing tumour growth (right panel)

In summary, our study provides compelling evidence of the suppressive function of TGF-β signalling on iCCA tumour cells, challenging the use of TGF-β receptor inhibitors. However, we have also demonstrated that inhibition of NOX4/NOX1, downstream effectors of TGF-β signalling, specifically impairs CAF functions and reduces CCA growth by modulating the TME (Fig. 9). Further evaluation of NOX4/NOX1 inhibitors in combination with current therapies is needed to exploit the full potential of these molecules. Our study also suggests that a deeper understanding of TGF-β actions in both tumour cells and the TME across different cancer types is imperative to identify new therapeutic targets downstream of TGF-β receptors that allow the specific inhibition of TGF-β pro-tumorigenic effects.

## Materials and methods

Further details on the experimental methods are available in the Supplementary Materials section online.

### Ethic approval statements

All experiments complied with the EU Directive 2010/63/UE for animal experiments and the corresponding institution’s guidelines. Procedures for the generation and treatment of the SB1 syngeneic and AKT-YAP and AKT-NICD hydrodynamic tail vein injection animal models were approved by the Ethical Committee for animal experimentation of IDIBELL and by the General Direction of Environment and Biodiversity, Government of Catalonia (#6292 and #4589). Procedures for the generation and treatment of PDX animal models were approved by the Ethical Committee for the Use of Experimental Animals at the Vall d’Hebron Institute of Research (VHIR) (number 40/21) and by the Catalan Government (number 11650).

The human tissues used in this study were collected with the required informed consent in written from each patient and the approval of the Institutional Review Board (Comite Etico de Investigacion Clínica-CEIC, University Hospital of Bellvitge) (Ref. PR350/21). Patients’ written consent form and the study protocol conformed to the ethical guidelines of the 1975 Declaration of Helsinki. CAF isolated in Barcelona were from resections from Hospital Clinic de Barcelona, under the HCB/2021/0732 ethical approval from the Hospital Clinic ethics committee. In the case of CAF isolated in Paris, the Committee for Personal Protection of Nord Ouest I (CPP) gave a favourable opinion on 11 April 2024 (National number: 2019-A01679-48 / SI number: 19.01424.061615-MS03.1/Internal ref: APHP190473). Participants signed an informed consent during the inclusion visit.

### Reagents

TGF-β1 (#T70039, Sigma-Aldrich, Saint Louis, MI, USA) was used at 2 ng/ml. Galunisertib (LY2157299) (S2230, Selleckchem, Houston, TX, USA) was used at 10 μM. Setanaxib (GKT137831) (A104916, AmBeed, Arlington Heights, IL, USA) was used at 40 μM. DPI (D2926 Sigma-Aldrich) was used at 2.5 μM.

### Cell culture

HuCCT1, SG231, CCLP1, and HuH28 cells, derived from human iCCA and TMNK1 derived from liver endothelial cells, were kindly provided by Dr. Laura Fouassier (CRSA, Paris, France). KKU-213A, KKU-213C and RBE cells, derived from human iCCA, were kindly provided by Dr. Gianluigi Giannelli (IRCCS “Saverio de Bellis”, Castellana Grotte, Italy). SB1, derived from murine iCCA, were kindly provided by Dr. Gregory J Gores and Dr. Sumera Ilyas (Mayo Clinic, Rochester, USA). LX2-HSC and hTERT-HSC cells derived from human hepatic stellate cells (HSC) were kindly provided by Dr. Lynda Aoudjehane (ICAN, Paris, France). HSC-GFP cells, human primary HSC transfected with GFP, were kindly provided by Dr. David G Mollevi (IDIBELL, Barcelona, Spain). All culture media and supplements were obtained from Gibco, Thermo Fisher Scientific (Waltham, MA, USA). THP-1 cells were purchased from the American Type Culture Collection (ATCC). HuCCT1, CCLP1, SG231, KKU-213A and KKU-213C cells were cultured in DMEM 1 g/L glucose (21885-025), 10 mmol/L HEPES (15630-056). HuH28 cells were cultured in MEM (41090-028). RBE and THP-1 cells were cultured in RPMI (61870-036) supplemented with HCO_3_^-^ (25080-060) 0.75%. SB1, LX2-HSC, hTERT-HSC and TMNK-1 cells were cultured in DMEM 4.5 g/l glucose (31966-021). HSC-GFP were cultured in DMEM/F-12 (10565018). All media were supplemented with antibiotics (100 UI/mL penicillin and 100 μg/mL streptomycin (15140-122)), antimycotic (0.25 mg/mL amphotericin B (15290018), and 10% FBS (10270-106). Cell lines were routinely screened for the presence of mycoplasma and authenticated for polymorphic markers to prevent cross-contamination. The medium was changed every three days and cells were maintained in a humidified atmosphere at 37 °C containing 5% CO_2_.

### NOX4 loss-of-function CRISPR-CAS9 cell model

To generate a pool of hTERT-HSC cells lacking NOX4 protein, we used the CRISPR-Cas9 system. Short-guide RNAs (gRNA) were designed to target the gene and then cloned into the pSpCas9(BB)-2A-puro vector (supplied by Addgene, Watertown, MA, USA), which encodes an RNA polymerase III promotor for the transcription of the guide, the Cas9 endonuclease, and a gene providing resistance to puromycin. hTERT-HSC cell lines were transfected for two hours with Lipofectamine® 2000 Reagent (Thermo Fisher Scientific). An empty vector without gRNA was used as the negative control. Puromycin was added for 48 h at 2 μg/mL for selection. The selected cells were then checked for mRNA and protein knockdown by RT-QPCR and immunoblot, respectively.

### RNA silencing

*NOX4* and *NOX1* expression was silenced by using the siRNAs listed in Supplementary Table 1. Transient transfections were performed with 100 nM siRNA using DharmaFECT 4 (Thermo Fisher Scientific). Cells were seeded in 12-well or 6-well plates and 24 h later they were transfected with the corresponding siRNA. 24 h after transfection cell culture media was changed to remove transfection complexes. Then, the appropriate protocol for each experiment was followed.

### Colony formation assay

Cells were plated in 6-well plates at a density of 500 cells/well and then they were cultured for 7-10 days. Colonies were fixed with 4% paraformaldehyde, stained with 1% crystal violet and counted. Only colonies with more than 50 cells were considered.

### Sphere formation assay

Spheroids were created using the hanging drop method. For all the experiments, cells were suspended, at a concentration of 300.000 cells/ml for tumour cells alone or 300.000 + 300.000 for mixed spheroids containing tumour cells and HSC-GFP in a proportion 1:1. Cells were suspended in medium with 0.24% of methylcellulose (M7027, Sigma-Aldrich) in the absence or presence of TGF-β1, galunisertib or setanaxib. Twenty-five µl drops were pipetted onto the lid of 100 mm dishes, that were inverted over dishes containing 5 ml of cell culture medium to prevent drying. After 4 days of incubation at 37 °C and 10% CO_2_, spheroids were transferred by pipetting onto a low-attachment 6-well culture plate (3471, Corning). Images of 10–20 spheroids per experiment were acquired with a NIKON Eclipse Ti2 microscope and spheroids size was measured with ImageJ software (National Institute of Health, USA).

### Experiments with CAF

#### CAF isolation

Human primary CAF were isolated by outgrowth from tumoral tissue obtained from iCCA resections. CAFs were maintained in DMEM Glutamax 10%FBS 1%PS and were used between passages 3 and 10.

#### 2D CAF experiment

Primary CAF derived from three different patients were used for this experiment. 50.000 cells were plated in a 12-well plate and were maintained in DMEM Glutamax 10%FBS 1% Penicillin/Streptomycin. When cells reached 70% confluence, they were starved using DMEM Glutamax 1%FBS 1% Penicillin/Streptomycin overnight and were treated with vehicle (DMSO), TGF-β1, setanaxib or the combination of both. 48 h later, cells were collected in lysis buffer (RIPA Buffer 1X (Sigma-Aldrich), Protease Inhibitor (Roche) and Phosphatase inhibitor (Roche)), for protein extraction.

#### 3D spheroids experiment

Spheroids were generated using human primary CAF (*n* = 1) and HuCCT1 cells using a 1:3 ratio. Briefly, CAF were stained using the Wheat Germ Agglutinin (WGA) Alexa Fluor 594 conjugate (Invitrogen), and HuCCT1 were stained with the Vybrant CFDA Cell Tracer (Invitrogen). A total of 20.000 cells were seeded in each well of a Corning Elplasia 96-well Round Bottom Ultra-Low Attachment plate, in DMEM Glutamax, 10%FBS and 1% Penicillin/Streptomycin, together with the correspondent treatment: vehicle (DMSO) or setanaxib. Plates were centrifuged at 1000 rpm for 1 minute and were placed in a cell culture incubator at 37 °C and 5% CO_2_. Pictures of the spheroids were acquired using a Nikon Eclipse Ti-S Inverted fluorescent microscopy on days 2, 4 and 7, and the area of the spheroids was measured using Image J software.

### CCA organoid ex vivo three-dimensional cultures

CCA patient-derived tumour cells were isolated from CCA_PDX through a combination of mechanic disruption and enzymatic disaggregation as previously described.^51^ Briefly, PDX tumours with a volume of less than 500 mm^3^ were freshly collected in DMEM/F12/HEPES (L0093-500, Biowest, Nuaillé, France) after surgical resection, minced using sterile scalpels, and dissociated for a maximum of 90 minutes in DMEM/F12/HEPES supplemented with 0.3 mg/mL collagenase (C9891, Sigma-Aldrich), 0.1 mg/mL hyaluronidase (H3506, Sigma-Aldrich), 2% BSA (VWRC0332, VWR, Radnor, PA, USA), 5 mg/mL insulin (I1882, Sigma-Aldrich), and 50 mg/mL gentamycin (15750-037, Gibco, Thermo Fisher Scientific). After centrifugation, pellets were further dissociated using 0.05% trypsin (HYCLSH30236.02, VWR), 5 mg/mL Dispase (7923, STEMCELL Technologies, Vancouver, Canada) and 1 mg/mL DNase (D4263, Sigma-Aldrich). Red blood cells were eliminated by washing the cell pellet with 1x Red Blood Cell Lysis Buffer solution (00-4333-57, eBioscience, San Diego, CA, USA).

For organoid generation, mouse stromal cell depletion was conducted using a commercial kit (Miltenyi Biotec, 130-104-694). The eluent containing human epithelial tumor cells fraction was resuspended in Matrigel Basement Membrane Matrix (Matrigel; #354277, Corning Corning, NY, USA) and seeded into pre-heated 6-well culture plates (140675, Thermo Fisher Scientific). As a standard, drops of 25 μL of the mixture were seeded into culture plates and incubated for 30 min at 37 °C without medium to allow the matrix solidification. Organoids were cultured in advanced DMEM/F12 medium supplemented with 1% penicillin/streptomycin, 10 mM HEPES, 1% Glutamax, 30% Wnt conditioned medium (from L1Wnt3A cell line), 15% RSPO1 conditioned medium (from 293tRSPO1mCh cell line) and 7.5% Noggin conditioned medium (from 293tnogginmCh cell line). The basal organoid medium was further supplemented with 1 x B27 supplement (17504044, Gibco, Thermo Fisher Scientific), 1x N2 supplement (17502048, Gibco, Thermo Fisher Scientific), 50 ng/ml human EGF (E9644, Sigma-Aldrich), 10 nM Gastrin I (Sigma G9145), 125 µg/ml Normocin (ant-nr-05, Invivogen, Toulouse, France), 2.5 µg/ml Plasmocin (ant-mpt-1, Invivogen). Four organoids were used in this study: PDXO85 (derived from liver metastasis), PDXO133 (derived from liver metastasis), PDXO153 (derived from bone metastasis) and PDXO162 (derived from peritoneum metastasis).

For conducting efficacy drug tests, organoids were dissociated to generate a single-cell suspension and embedded in Matrigel (CLS356234) in order to seed at a density of 2 × 10^4^ cells per well in pre-heated Corning 96-Well White Polystyrene Microplates (CLS3610, Corning). Following organoid generation (24-48 h later) cells were treated with either vehicle (DMSO) or the corresponding drug and cultured at 37 °C in 5% of CO_2_ for 4 days. Medium and treatments were refreshed every 2–3 days. Cell viability was measured by CellTiter-Glo assay. Matrigel was melted by direct on-plate incubation with 50 μL PBS with1 mmol/L EDTA for 1 hour at 4 °C protected from light. Cell viability was quantified using CellTiter-Glo Luminescent Cell Viability Assay (Promega; G7570), according to the manufacturer’s instructions. Luminescence was measured with infinite M2000 Pro (Tecan) and i-control 1.11 software.

### Gene expression profiling

Gene expression datasets established from laser microdissected^52^ CCA tumours were previously described. Freshly frozen tumour samples were obtained through the French liver cancer biobanks network – INCa (BB-0033-00085).

### Comparison of mRNA expression from public databases

#### TCGA cohort

The mRNA expression levels of *KRT19*, *ACTA2*, *COL1A1*, *TGFB1*, *TGFB2*, *TGFB3*, *TGFBR1*, *TGFBR2, TGFBR3 and NOX4* in non-tumoral tissue and CCA tumours from the repository of The Cancer Genome Atlas (TCGA) were analysed through the GEPIA database (http://gepia.cancer-pku.cn/)^53^ and were represented as means ± SEM. The mRNA expression levels of *TGFB1 versus SMAD7* and *NOX4* *versus ACTA2, COL1A1*, *LOXL1* and *LOXL2* in CCA tumours from the repository of TCGA were analysed through the cBioPortal database (https://www.cbioportal.org/) and represented as Spearman correlation coefficients (r) and *p*-values to assess the significance.^54,55^

#### TIGER-LC cohort

Raw microarray data was downloaded from Gene Expression Omnibus (accession ID: GSE76297) (TIGER-LC cohort) and processed using robust multiarray method implemented in the affy package version 1.56 available through the Bioconductor software project (https://bioconductor.org) using R v4.0.4.^56^ Probe-set to gene mapping was done using the array annotation file available at Gemma database (https://gemma.msl.ubc.ca/arrays/showArrayDesign.html?id=785), selecting the most expressed probe as representative of gene expression when multiple mapping probes occurred to avoid duplicated genes. Comparison of median gene expression of *TGFB1*, *TGFB2*, *TGFB3*, *TGFBR1*, *TGFBR2*, *TGFBR3* and *NOX4* between tumour and non-tumour samples was done with Mann–Whitney U test and Spearman correlation analysis was performed to assess the correlation of gene expression between *TGFB1* and *SMAD7*.

#### Single-cell RNA-seq

The single-cell RNA-seq dataset was processed, analysed and visualized using the Trailmaker® (https://scp.biomage.net/) hosted by Biomage (https://biomage.net/). First, pre-filtered count matrices from Shi et al. (GSE201425) were uploaded to Trailmaker®. Briefly, to pre-process the data, barcodes with low unique molecular identifiers (UMIs) and or high mitochondrial reads were filtered out. Next, to exclude outliers, a robust linear model was fitted to all samples. Lastly, barcodes with high doublet scores were excluded from the analysis, resulting in high quality barcodes that were used in subsequent integration steps. Briefly, to integrate the barcodes, data were log-normalised and the top 2000 highly variable genes were selected. Next, principal component analysis (PCA) was performed, and the top 30 principal components, explaining 89.67% of the total variance, were used to perform sample batch correction with the integrated Harmony R package. Louvain method of clustering was employed. To reduce dimensionality, data was visualised using t-distributed stochastic neighbour embedding (t-SNE). To identify cluster-specific marker genes, the marker genes of each cluster were compared to all other clusters and appropriately annotated. The CAF subset identified from this analysis was further examined via the sample subset feature. Filtering steps were disabled (due to pre-filtered data) and the subset was analysed via the same integration steps outlined above. Signatures of upregulated genes in CAF subtypes were identified (adj. *p*-value < 0.05, expression of the gene in at least 1/3 of the cells, and average log2FC > 1) in the scRNA-seq data using Seurat. These gene-expression signatures were then deconvoluted in the bulk RNA-seq experiments using Gene Set Variation Analysis (GSVA),^57^ which provides the relative enrichment of a gene-expression signature for each sample, ranging from -1 to 1, where higher values are associated with more relative enrichment in a specific sample compared to the others.

#### Survival analyses

Survival analyses were conducted in the GSE244807 dataset, which had available gene expression and overall survival data for 246 patients with intrahepatic cholangiocarcinoma (iCCA).^58^ Kaplan-Meier curves were plotted, and hazard ratios and 95% confidence intervals were obtained from a Cox Proportional Hazards model adjusted for sex and age clinical covariates. Gene set variation analysis (GSVA) was used to assess the relative activation of the setanaxib treatment signature in the samples.^57^ Median GSVA scores, in the case of signatures, or median gene expression value, in the case of individual genes (i.e. NOX1, NOX4), were used to stratify patients in low or high group, respectively. All analyses were performed using R 4.0.4.

#### Active CD4 T cells infiltration evaluation

Active CD4 T cell signatures were obtained from a previous study.^59^ GSVA was used to assess the relative infiltration of CD4 T cells in the samples.^57^ Differences in median CD4 T cell infiltration scores between setanaxib signature low and setanaxib signature high groups were tested using a Mann- Whitney U test. *P*-values were corrected for multiple testing with False Discovery Rate (FDR).

### RNA-seq analysis

#### Short-Read RNA Sequencing

Total RNA quantification was performed using the Qubit® RNA BR Assay kit (Thermo Fisher Scientific), and the RNA integrity was assessed using the RNA 6000 Nano Bioanalyzer 2100 Assay (Agilent Technologies, Santa Clara, CA, USA). To prepare the RNASeq libraries, the KAPA Stranded mRNA-Seq Illumina Platforms Kit (Roche, Basel, Switzeland) was used, following the manufacturer’s recommendations with 500 ng of total RNA as the input material. Library quality was assessed on an Agilent 2100 Bioanalyzer using the DNA 7500 assay. The libraries were sequenced on the NovaSeq 6000 (Illumina, San Diego, CA, USA) with a read length of 2 × 151 bp, in accordance with the manufacturer’s protocol for dual indexing. Image analysis, base calling, and quality scoring of the run were executed using the manufacturer’s Real Time Analysis (RTA 3.4.4) software.

#### RNA-seq data processing and analysis

RNA-seq reads were trimmed using Trim Galore (https://github.com/FelixKrueger/TrimGalore), setting the length parameter at 35 and the stringency at 10. Trimmed reads were mapped against the human reference genome (GRCh38) using STAR aligner version 2.7.8a^60^ with ENCODE parameters, and annotated genes were quantified with RSEM version 1.3.0^61^ using GENCODE v42 as reference. Differential expression analysis was performed with limma v3.4.2 R package, using TMM normalisation. The ‘voom’ function^62^ was used to estimate the mean-variance relationship and to compute observation-level weights. The linear model was fitted for each cell line with the corresponding voom-transformed counts, and contrasts were extracted. Genes were considered differentially expressed (DEG) with a *p*-value adjusted below 0.05 and subsets of DEG were represented in heatmaps with the pheatmap R package, using voom-transformed counts scaled by row. A gene set enrichment analysis (GSEA) was performed on each list of genes pre-ranked by the limma moderated t-statistic, with the fgsea v1.12 R package,^63^ against the Reactome database.

### RNA and reverse transcription-PCR

E.Z.N.A.® Total RNA Kit II (Omega bio-tek, Norcross, GA, USA) was used for total RNA isolation following the manufacturer’s instructions. Culture plates were washed with PBS and cells were detached with Trypsin and pelleted by centrifugation. Pellets were dissolved in RLT lysis buffer containing 10 μL/mL of β-mercaptoethanol. 1 μg of total RNA isolated from each sample was reverse-transcribed with random primers for complementary DNA synthesis, using a High Capacity RNA to cDNA Master Mix Kit (Applied Biosystems, Foster City, CA, USA), according to the manufacturer’s protocol. For the real-time qPCR, expression levels were determined in duplicate in a LightCycler® 480 Real-time PCR system, using the LightCycler® 480 SYBR Green I Master (Roche Diagnostics GmbH, Mannheim, Germany). Primer sequences are provided in Supplementary Tables 2 and 3. Gene expression was normalised to *GAPDH* mRNA content for human genes and to *Rpl32* mRNA content for mouse genes and was expressed relatively to the control condition of each experiment. The relative expression of each target gene was determined from replicate samples using the formula 2^-ΔΔCt^.

### Immunoblot analysis

For obtaining whole-cell lysates for immunoblotting, cell cultures were lysed in RIPA buffer (Sigma-Aldrich) supplemented with 1 mmol/L orthovanadate and a cocktail of protease inhibitors. Proteins were quantified using a Pierce BCA kit (Thermo Fisher Scientific). Western blot analysis was performed as previously described.^64^ Primary antibodies are provided in Supplementary Table 4.

#### Amplex red

Extracellular hydrogen peroxide levels were detected using an Amplex® Red assay kit (A36006, Invitrogen, Thermo Fisher Scientific). Cells were seeded on 12-well plates in DMEM with 10% FBS. The next day, cells were deprived of serum for 24 h in DMEM without phenol red (21063-029) at 0.5% FBS and cultured with the corresponding treatment for 24 h. Then, the media was refreshed containing Amplex® Red reagent (10 μM) and horseradish peroxidase (0.1 U/mL). After 2 h incubation at 37 °C, fluorescence was measured in duplicate in a BMG FLUOstar OPTIMA fluorescence plate reader (BMG Labtech, Ortenberg, Germany) with excitation and emission at 530 nm and 590 nm, respectively. H_2_O_2_ concentration was extrapolated using a standard curve and values were normalised to cell viability by crystal violet staining (61135, Sigma-Aldrich).

### Immunohistochemical analysis

#### Tumour samples

Sections of 5 μm in thickness were used for subsequent assays. For CK19 IHC staining, antigen retrieval was achieved in TE buffer (pH 8.0), while sodium citrate buffer (pH 6.0) was used for all other targets. Slides were submerged in retrieval buffer and heated in a microwave on a high level for 10 minutes. After cooling down, slices were blocked with goat serum and the Avidin-Biotin blocking kit (Vector Laboratories, Burlingame, CA). Specimens were incubated with the designated primary antibody at 4 °C overnight. Detailed information on the antibodies used is listed in Supplementary Table 4. Subsequently, 3% hydrogen peroxide was applied for 10 min to quench endogenous peroxidase activity. Slices were incubated in the biotin-conjugated secondary antibody (Life Technology, Waltham, MA) at a 1:500 dilution for 30 min at room temperature. The Vectastain Elite ABC Kit (Vector Laboratories) and DAB substrates (Dako North America, Carpinteria, CA) were applied to visualise the immunostainings. Naphthol-AS-D-Chloracetatesterase (CLAE) histochemistry was performed according to standard diagnostic practice.

For analysis of collagen deposition, 5 μm-cut paraffin-embedded tissue sections were dewaxed and rehydrated in an Autostainer XL (Leica Biosystems, Deer Park, IL, USA) and stained with Picro Sirius Red (Picric acid 197378; Direct-Red 80 365548, Sigma-Aldrich).

Stained sections were scanned on a virtual slide scanner Pannoramic 1000 Flash RX® (Sysmex Europe SE, Norderstedt, Germany) and images were analysed blindly by a pathologist, AS, and two investigators EG-S and JV. Quantification of positive cells or positive-stained areas was performed using Slideviewer and ImageJ analysis software (National Institutes of Health, Bethesda, MD, USA) respectively. Quantitative analyses were conducted in at least 10 tumours per mouse. In the case of intratumoural CD4-positive cells, only the inner area of the tumour was considered.

#### PDXO samples

Organoid samples were analysed by Next Generation IHC (NGI) using the tumor-Panel to assess. Briefly, Organoids were harvested from culture and processed for paraffin embedding. The organoid suspension was centrifuged at 300 x g for 5 minutes at 4 °C. The pellet was washed twice with ice-cold PBS and fixed in 4% paraformaldehyde for 60 minutes at 4 °C. Post-fixation, the sample was centrifuged (300 x g, 5 minutes, 4 °C) and washed once with PBS. For agarose embedding, the organoid pellet was resuspended in 100 μL of the 2% agarose solution. The agarose was allowed to solidify completely at 4 °C for approximately 30 minutes. The agarose block was dehydrated and embedded in FFPE. Pan-cytokeratin (pan-CK) was included as a marker for iCCA tumor cells, Vimentin (Vim) for the detection of murine stromal cells and Ki67 staining was included as a marker of cell proliferation. The NGI protocol consists of iterative cycles of staining/destaining on the same tissue section and uses the combination of Ventana Discovery Ultra (Roche Diagnostics), Nanozoomer slide scanner (Hamamatsu) and Visiopharm image analysis software. Briefly, an alcohol soluble chromogen (DISCOVERY AEC KIT (#760-258, Roche-Ventana)) was used to allow the destaining of the samples. After each automated IHC, samples were mounted in aqueous medium and digitalized (cycle 1). Subsequently, the section was destained in alcohol and submitted to the staining cycle including all the markers. Positive controls were included in each slide. Antibodies and protocols used in this study are listed in Supplementary Table 4.

### CCA murine models

#### Syngeneic, orthotopic mouse model of CCA

NOX4^−/−^ (B6.129-Nox4tm1Kkr/J) mice, generated in Dr. Krause’s Laboratory,^65^ were obtained from Jackson Laboratories (together with C57BL/6 J mice, the appropriated controls, as suggested by the provider) and housed at IDIBELL (Barcelona, Spain). Murine SB1 iCCA cells derived from an oncogene-driven murine model of iCCA were maintained in the culture medium, as previously described.^18^ Mice were anesthetised using 1.5% to 3% isoflurane (IsoFlo 250 ml, Zoetis). Under deep anaesthesia, the abdominal cavity was opened by a 1 cm incision below the xiphoid process. A sterile cotton-tipped applicator was used to expose the superolateral aspect of the medial lobe of the liver. Using a 27-gauge needle, 40 μL of standard media containing 0.5 × 10^6^ SB1 cells were injected into the lateral aspect of the medial lobe. A cotton-tipped applicator was held over the injection site to prevent cell leakage and blood loss. Subsequently, the abdominal wall and skin were closed in separate layers with absorbable chromic 3-0 gut suture material. Four weeks following SB1 cell implantation, mice were sacrificed, and tumour and adjacent liver were collected for the different analyses.

#### Hydrodynamic Tail Vein Injection model of CCA

WT male FVB/N mice were purchased from Charles River (Charles River Laboratories Les Oncins, France). The hydrodynamic injection was performed as described previously.^19^ In brief, 20 μg pT3-EF1α-HA-myr-AKT and 20 μg pT3-EF1α-YapS127A (AKT-YAP model) or 20 μg pT3-EF1α-HA-myr-AKT and 20 μg pT3-EF1α-NICD1 (AKT-NICD model), with 2 μg pCMV/sleeping beauty transposase (SB) were diluted in 2 mL of saline (0.9% NaCl), filtered through 0.22-μm filter, and injected into the lateral tail vein of 6- to 8-week-old mice in 5 to 7 seconds. Four weeks after the injection, when the tumours were developed, 150 mg/kg of galunisertib, 60 mg/kg of setanaxib or the vehicle (1% (w/v) carboxymethylcellulose (#C9481 Sigma-Aldrich, 0.25% (v/v) Tween80 (#59924 Sigma-Aldrich), and 0.05% (v/v) Antifoam (#59920 C Sigma-Aldrich,) in purified water) were given to the mice daily during two weeks prior to sacrifice for the obtention of tissue samples.

#### PDX generation and treatment

Human PDX153 was generated by subcutaneous implantation of a metastatic liver biopsy from a patient diagnosed with intrahepatic CCA. PDX153 tumour pieces (~1-2mm^3^) were subcutaneously implanted into the right and left flanks of 6- to 8-week-old female NOD.CB-17-Prkdc scid/Rj mice (Janvier Labs, RRID:MGI:3760616). Animals were housed in air-filtered flow cages with a 12:12 light/dark cycle, and food and water were provided ad libitum.

Upon xenograft growth (50-150 mm^3^), PDX153-bearing mice were randomized into 3 groups for treatment with vehicle, setanaxib 60 mg/kg, or galunisertib 150 mg/kg daily by oral gavage for 22 days. All drugs were dissolved in vehicle [1% (w/v) carboxymethylcellulose (#C9481 Sigma-Aldrich, 0.25% (v/v) Tween80 (#59924 Sigma-Aldrich), and 0.05% (v/v) Antifoam (#59920 C Sigma-Aldrich]. Tumour growth was measured twice per week with a calliper; investigators were blinded to treatment effect. Mice weights were recorded two times per week. Tumour volumes were calculated using the formula: V = (length x width^2^)/2. Mice were euthanized by CO_2_ inhalation when tumours reached 1-1.5 cm^3^ or severe weight loss occurred, according to institutional guidelines. PDX tumours were harvested at the end of the experiment.

Animals were hosted under 12 h light/dark cycles with free access to food and water.

### CCA specimens

Samples from tumour tissues and surrounding non-tumoral liver were obtained from patients during surgical procedures (transplantation or resection) at the Bellvitge University Hospital (HUB) (Supplementary Table 5).

### Statistical analysis

Results were analysed using the GraphPad Prism 5.0 statistical software (GraphPad Software, Boston, MA, USA). Data are shown as means ± standard error of the mean (SEM). For comparisons between two groups, parametric Student t tests or nonparametric Mann–Whitney tests were used.

## Supplementary information

Supplementary Material

## Data Availability

RNA-seq datasets generated in this study are available at Gene Expression Omnibus (GEO) under the accession number GSE274024. The rest of the data produced in this study can be found in the article and its supplementary data files. The publicly available data used in this study, which were generated by other researchers, were sourced from TCGA-CHOL and GEO databases under the accession numbers GSE76297, GSE244807 and GSE201425.

## References

[1] Banales, J. M. et al. Expert consensus document: Cholangiocarcinoma: current knowledge and future perspectives consensus statement from the European Network for the Study of Cholangiocarcinoma (ENS-CCA). *Nat. Rev. Gastroenterol. Hepatol.* **13**, 261–280 (2016). 27095655 10.1038/nrgastro.2016.51

[2] Banales, J. M. et al. Cholangiocarcinoma 2020: the next horizon in mechanisms and management. *Nat. Rev. Gastroenterol. Hepatol.* **17**, 557–588 (2020). 32606456 10.1038/s41575-020-0310-z PMC7447603

[3] Oh, D. Y. et al. Plain language summary of the TOPAZ-1 study: durvalumab and chemotherapy for advanced biliary tract cancer. *Future Oncol.* **19**, 2277–2289 (2023). 37746835 10.2217/fon-2023-0468

[4] Rimini, M. et al. Durvalumab plus gemcitabine and cisplatin in advanced biliary tract cancer: An early exploratory analysis of real-world data. *Liver Int* **43**, 1803–1812 (2023). 37452505 10.1111/liv.15641

[5] Kelley, R. K. et al. Pembrolizumab in combination with gemcitabine and cisplatin compared with gemcitabine and cisplatin alone for patients with advanced biliary tract cancer (KEYNOTE-966): a randomised, double-blind, placebo-controlled, phase 3 trial. *Lancet* **401**, 1853–1865 (2023). 37075781 10.1016/S0140-6736(23)00727-4

[6] Skouteris, N. et al. Immune checkpoint inhibitors and combinations with other agents in cholangiocarcinoma. *Immunotherapy* **15**, 487–502 (2023). 36876442 10.2217/imt-2022-0225

[7] Oh, D. Y. et al. Bintrafusp alfa and chemotherapy as first-line treatment in biliary tract cancer: A randomized phase 2/3 trial. *Hepatology* **8**, 823–836 (2025). 10.1097/HEP.0000000000000965 PMC11825481 38875119

[8] Massague, J. & Sheppard, D. TGF-beta signaling in health and disease. *Cell* **186**, 4007–4037 (2023). 37714133 10.1016/j.cell.2023.07.036 PMC10772989

[9] Gonzalez-Sanchez, E. et al. The TGF-beta Pathway: A Pharmacological Target in Hepatocellular Carcinoma?. *Cancers (Basel)* **13**, 3248 (2021). 34209646 10.3390/cancers13133248 PMC8268320

[10] Tindall, R. R., Bailey-Lundberg, J. M., Cao, Y. & Ko, T. C. The TGF-beta superfamily as potential therapeutic targets in pancreatic cancer. *Front Oncol.* **14**, 1362247 (2024). 38500662 10.3389/fonc.2024.1362247 PMC10944957

[11] Papoutsoglou, P., Louis, C. & Coulouarn, C. Transforming Growth Factor-Beta (TGFbeta) Signaling Pathway in Cholangiocarcinoma. *Cells* **8**, 960 (2019). 31450767 10.3390/cells8090960 PMC6770250

[12] Gong, S., Wang, S. & Shao, M. NADPH Oxidase 4: A Potential Therapeutic Target of Malignancy. *Front Cell Dev. Biol.* **10**, 884412 (2022). 35646942 10.3389/fcell.2022.884412 PMC9130727

[13] Crosas-Molist, E. et al. The NADPH oxidase NOX4 inhibits hepatocyte proliferation and liver cancer progression. *Free Radic. Biol. Med*. **69**, 338–347 (2014). 24509161 10.1016/j.freeradbiomed.2014.01.040

[14] Shanmugasundaram, K. et al. NOX4 functions as a mitochondrial energetic sensor coupling cancer metabolic reprogramming to drug resistance. *Nat. Commun.* **8**, 997 (2017). 29051480 10.1038/s41467-017-01106-1 PMC5648812

[15] Hanley, C. J. et al. Targeting the Myofibroblastic Cancer-Associated Fibroblast Phenotype Through Inhibition of NOX4. *J. Natl Cancer Inst.* **110**, 109–120 (2018). 28922779 10.1093/jnci/djx121 PMC5903651

[16] Siedlar, A. M. et al. NADPH oxidase 4 is dispensable for skin myofibroblast differentiation and wound healing. *Redox Biol.* **60**, 102609 (2023). 36708644 10.1016/j.redox.2023.102609 PMC9950659

[17] Calvisi, D. F. et al. Criteria for preclinical models of cholangiocarcinoma: scientific and medical relevance. *Nat. Rev. Gastroenterol. Hepatol.* **20**, 462–480 (2023). 36755084 10.1038/s41575-022-00739-y

[18] Ilyas, S. I. et al. YAP-associated chromosomal instability and cholangiocarcinoma in mice. *Oncotarget* **9**, 5892–5905 (2018). 29464042 10.18632/oncotarget.23638 PMC5814182

[19] Chen, X. & Calvisi, D. F. Hydrodynamic transfection for generation of novel mouse models for liver cancer research. *Am. J. Pathol.* **184**, 912–923 (2014). 24480331 10.1016/j.ajpath.2013.12.002 PMC3969989

[20] Chen, Y. et al. TGF-beta1 expression is associated with invasion and metastasis of intrahepatic cholangiocarcinoma. *Biol. Res*. **48**, 26 (2015). 25993985 10.1186/s40659-015-0016-9 PMC4513632

[21] Miyaki, M. & Kuroki, T. Role of Smad4 (DPC4) inactivation in human cancer. *Biochem Biophys. Res Commun.* **306**, 799–804 (2003). 12821112 10.1016/s0006-291x(03)01066-0

[22] Coulouarn, C., Factor, V. M. & Thorgeirsson, S. S. Transforming growth factor-beta gene expression signature in mouse hepatocytes predicts clinical outcome in human cancer. *Hepatology* **47**, 2059–2067 (2008). 18506891 10.1002/hep.22283 PMC2762280

[23] Malfettone, A. et al. Transforming growth factor-beta-induced plasticity causes a migratory stemness phenotype in hepatocellular carcinoma. *Cancer Lett.* **392**, 39–50 (2017). 28161507 10.1016/j.canlet.2017.01.037

[24] Caballero-Diaz, D. et al. Clathrin switches transforming growth factor-beta role to pro-tumorigenic in liver cancer. *J. Hepatol.* **72**, 125–134 (2020). 31562907 10.1016/j.jhep.2019.09.012

[25] Yan, X. & Chen, Y. G. Smad7: not only a regulator, but also a cross-talk mediator of TGF-beta signalling. *Biochem J.* **434**, 1–10 (2011). 21269274 10.1042/BJ20101827

[26] Yingling, J. M. et al. Preclinical assessment of galunisertib (LY2157299 monohydrate), a first-in-class transforming growth factor-beta receptor type I inhibitor. *Oncotarget* **9**, 6659–6677 (2018). 29467918 10.18632/oncotarget.23795 PMC5805504

[27] Bertran, E. et al. Overactivation of the TGF-beta pathway confers a mesenchymal-like phenotype and CXCR4-dependent migratory properties to liver tumor cells. *Hepatology* **58**, 2032–2044 (2013). 23813475 10.1002/hep.26597

[28] Ferraro, A. et al. Tumor suppressor role of the CL2/DRO1/CCDC80 gene in thyroid carcinogenesis. *J. Clin. Endocrinol. Metab.* **98**, 2834–2843 (2013). 23666966 10.1210/jc.2012-2926

[29] Haldrup, J. et al. FRMD6 has tumor suppressor functions in prostate cancer. *Oncogene* **40**, 763–776 (2021). 33249427 10.1038/s41388-020-01548-w

[30] Seubwai, W., Wongkham, C., Puapairoj, A., Khuntikeo, N. & Wongkham, S. Overexpression of vitamin D receptor indicates a good prognosis for cholangiocarcinoma: implications for therapeutics. *Cancer* **109**, 2497–2505 (2007). 17487855 10.1002/cncr.22716

[31] Meitzler, J. L. et al. Decoding NADPH oxidase 4 expression in human tumors. *Redox Biol.* **13**, 182–195 (2017). 28578276 10.1016/j.redox.2017.05.016 PMC5458090

[32] Lu, J. et al. NADPH oxidase 1 is highly expressed in human large and small bowel cancers. *PLoS One* **15**, e0233208 (2020). 32428030 10.1371/journal.pone.0233208 PMC7237001

[33] Invernizzi, P. et al. Setanaxib, a first-in-class selective NADPH oxidase 1/4 inhibitor for primary biliary cholangitis: A randomized, placebo-controlled, phase 2 trial. *Liver Int* **43**, 1507–1522 (2023). 37183520 10.1111/liv.15596

[34] Cui, W. et al. NOX1/nicotinamide adenine dinucleotide phosphate, reduced form (NADPH) oxidase promotes proliferation of stellate cells and aggravates liver fibrosis induced by bile duct ligation. *Hepatology* **54**, 949–958 (2011). 21618578 10.1002/hep.24465

[35] Song, X. et al. Focal adhesion kinase (FAK) promotes cholangiocarcinoma development and progression via YAP activation. *J. Hepatol.* **75**, 888–899 (2021). 34052254 10.1016/j.jhep.2021.05.018 PMC8453055

[36] Nicolas-Boluda, A. et al. Tumor stiffening reversion through collagen crosslinking inhibition improves T cell migration and anti-PD-1 treatment. *Elife* **10**, e58688 (2021). 34106045 10.7554/eLife.58688 PMC8203293

[37] David, C. J. et al. TGF-beta Tumor Suppression through a Lethal EMT. *Cell* **164**, 1015–1030 (2016). 26898331 10.1016/j.cell.2016.01.009 PMC4801341

[38] Guasch, G. et al. Loss of TGFbeta signaling destabilizes homeostasis and promotes squamous cell carcinomas in stratified epithelia. *Cancer Cell* **12**, 313–327 (2007). 17936557 10.1016/j.ccr.2007.08.020 PMC2424201

[39] Dong, M. et al. Efficacy of MEK inhibition in a K-Ras-driven cholangiocarcinoma preclinical model. *Cell Death Dis.* **9**, 31 (2018). 29348467 10.1038/s41419-017-0183-4 PMC5833851

[40] Bailey, P. et al. Genomic analyses identify molecular subtypes of pancreatic cancer. *Nature* **531**, 47–52 (2016). 26909576 10.1038/nature16965

[41] Valle, J. W., Lamarca, A., Goyal, L., Barriuso, J. & Zhu, A. X. New Horizons for Precision Medicine in Biliary Tract Cancers. *Cancer Discov.* **7**, 943–962 (2017). 28818953 10.1158/2159-8290.CD-17-0245 PMC5586506

[42] Ford, K. et al. NOX4 Inhibition Potentiates Immunotherapy by Overcoming Cancer-Associated Fibroblast-Mediated CD8 T-cell Exclusion from Tumors. *Cancer Res*. **80**, 1846–1860 (2020). 32122909 10.1158/0008-5472.CAN-19-3158 PMC7611230

[43] Crestani, B., Besnard, V. & Boczkowski, J. Signalling pathways from NADPH oxidase-4 to idiopathic pulmonary fibrosis. *Int J. Biochem Cell Biol.* **43**, 1086–1089 (2011). 21513813 10.1016/j.biocel.2011.04.003

[44] Barnes, J. L. & Gorin, Y. Myofibroblast differentiation during fibrosis: role of NAD(P)H oxidases. *Kidney Int* **79**, 944–956 (2011). 21307839 10.1038/ki.2010.516 PMC3675765

[45] Sampson, N. et al. Inhibition of Nox4-dependent ROS signaling attenuates prostate fibroblast activation and abrogates stromal-mediated protumorigenic interactions. *Int J. Cancer* **143**, 383–395 (2018). 29441570 10.1002/ijc.31316 PMC6067067

[46] Mir, S. et al. Upregulation of Nox4 induces a pro-survival Nrf2 response in cancer-associated fibroblasts that promotes tumorigenesis and metastasis, in part via Birc5 induction. *Breast Cancer Res*. **24**, 48 (2022). 35836253 10.1186/s13058-022-01548-6 PMC9281082

[47] Huang, H. et al. Mesothelial cell-derived antigen-presenting cancer-associated fibroblasts induce expansion of regulatory T cells in pancreatic cancer. *Cancer Cell* **40**, 656–673 (2022). 35523176 10.1016/j.ccell.2022.04.011 PMC9197998

[48] Sun, L. et al. PD-L1 promotes myofibroblastic activation of hepatic stellate cells by distinct mechanisms selective for TGF-beta receptor I versus II. *Cell Rep.* **38**, 110349 (2022). 35139382 10.1016/j.celrep.2022.110349 PMC8903892

[49] Carapeto, F. et al. The immunogenomic landscape of resected intrahepatic cholangiocarcinoma. *Hepatology* **75**, 297–308 (2022). 34510503 10.1002/hep.32150 PMC8766948

[50] Xia, T. et al. Immune cell atlas of cholangiocarcinomas reveals distinct tumor microenvironments and associated prognoses. *J. Hematol. Oncol.* **15**, 37 (2022). 35346322 10.1186/s13045-022-01253-z PMC8962046

[51] Serra-Camprubi, Q. et al. Human Metastatic Cholangiocarcinoma Patient-Derived Xenografts and Tumoroids for Preclinical Drug Evaluation. *Clin. Cancer Res*. **29**, 432–445 (2023). 36374558 10.1158/1078-0432.CCR-22-2551 PMC9873249

[52] Sulpice, L. et al. Molecular profiling of stroma identifies osteopontin as an independent predictor of poor prognosis in intrahepatic cholangiocarcinoma. *Hepatology* **58**, 1992–2000 (2013). 23775819 10.1002/hep.26577

[53] Tang, Z. et al. GEPIA: a web server for cancer and normal gene expression profiling and interactive analyses. *Nucleic Acids Res* **45**, W98–W102 (2017). 28407145 10.1093/nar/gkx247 PMC5570223

[54] Cerami, E. et al. The cBio cancer genomics portal: an open platform for exploring multidimensional cancer genomics data. *Cancer Discov.* **2**, 401–404 (2012). 22588877 10.1158/2159-8290.CD-12-0095 PMC3956037

[55] Gao, J. et al. Integrative analysis of complex cancer genomics and clinical profiles using the cBioPortal. *Sci. Signal* **6**, pl1 (2013). 23550210 10.1126/scisignal.2004088 PMC4160307

[56] Irizarry, R. A. et al. Exploration, normalization, and summaries of high density oligonucleotide array probe level data. *Biostatistics* **4**, 249–264 (2003). 12925520 10.1093/biostatistics/4.2.249

[57] Hanzelmann, S., Castelo, R. & Guinney, J. GSVA: gene set variation analysis for microarray and RNA-seq data. *BMC Bioinforma.* **14**, 7 (2013). 10.1186/1471-2105-14-7 PMC3618321 23323831

[58] Beaufrère A, L. T. et al. Self-supervised learning to predict intrahepatic cholangiocarcinoma transcriptomic classes on routine histology. bioRxiv; 2024. p. 2024.01.15.575652. 10.1016/j.jhepr.2025.101675 PMC12800354 41541502

[59] Charoentong, P. et al. Pan-cancer Immunogenomic Analyses Reveal Genotype-Immunophenotype Relationships and Predictors of Response to Checkpoint Blockade. *Cell Rep.* **18**, 248–262 (2017). 28052254 10.1016/j.celrep.2016.12.019

[60] Dobin, A. et al. STAR: ultrafast universal RNA-seq aligner. *Bioinformatics* **29**, 15–21 (2013). 23104886 10.1093/bioinformatics/bts635 PMC3530905

[61] Li, B. & Dewey, C. N. RSEM: accurate transcript quantification from RNA-Seq data with or without a reference genome. *BMC Bioinforma.* **12**, 323 (2011). 10.1186/1471-2105-12-323 PMC3163565 21816040

[62] Law, C. W., Chen, Y., Shi, W. & Smyth, G. K. voom: Precision weights unlock linear model analysis tools for RNA-seq read counts. *Genome Biol.* **15**, R29 (2014). 24485249 10.1186/gb-2014-15-2-r29 PMC4053721

[63] Korotkevich, G. e. a. F. g. s. e. a. b., 060012, 10.1101/060012 (2021).

[64] Vaquero, J. et al. The IGF2/IR/IGF1R Pathway in Tumor Cells and Myofibroblasts Mediates Resistance to EGFR Inhibition in Cholangiocarcinoma. *Clin. Cancer Res* **24**, 4282–4296 (2018). 29716918 10.1158/1078-0432.CCR-17-3725

[65] Carnesecchi, S. et al. A key role for NOX4 in epithelial cell death during development of lung fibrosis. *Antioxid. Redox Signal* **15**, 607–619 (2011). 21391892 10.1089/ars.2010.3829 PMC3163392

