# Supplementary material for: NADPH oxidase 1/4 dual inhibition impairs transforming growth factor-beta protumorigenic effects in cholangiocarcinoma cancer-associated fibroblasts
Source: Signal Transduct Target Ther. 2025 Aug 18;10:257. doi: 10.1038/s41392-025-02347-z (PMC12358586; doi:10.1038/s41392-025-02347-z)
Supplement: Supplementary file 1 — Supplementary Material [file 41392_2025_2347_MOESM1_ESM.docx]

Supplementary Materials for

NOX4/NOX1 dual inhibition in cholangiocarcinoma as an effective approach to target TGF-beta protumorigenic actions in cancer associated fibroblasts

Josep Amengual^1,2&^, Ester Gonzalez-Sanchez^1,2,3,4&^, Mariana Yañez^5^, Laura Sererols-Viñas^6^, Aashreya Ravichandra^7^, Celia Guiton^1^, Noel P Fuste^1^, Ania Alay^8,9^, Sara Hijazo-Pechero^9^, Beatriz Martín-Mur^10,11^, Marta Gut^10,11^, Anna Esteve-Codina^10,11^, Ana Cantos-Cortes^1,2^, Rut Espinosa-Sotelo^1,2^, Emilio Ramos^2,12,13^, Teresa Serrano^2,12,14^, Mariona Calvo^1,15^, Berta Laquente^15^, Joana Ferrer^2,16^, Gabriel Pons^17^, Andrés Mendez-Lucas^17,18^, Steven Dooley^19^, Sumera I Ilyas^20^, Marie Vallette^21^, Lynda Aoudjehane^21,22,23^, Marie Lequoy^21,24^, Laura Fouassier^21^, Cédric Coulouarn^25^, Silvia Affò^6^, Alexander Scheiter^26^, Diego F Calvisi^26^, Tian V Tian^5^, Isabel Fabregat^1,2#^, Javier Vaquero^1,2,4#*^

Correspondence to: javier.vaquero@usal.es

**This PDF file includes:**

Materials and Methods

Tables S1 to S5

Figures. S1 to S31

**Tables**

| **Supplementary Table 1.** siRNA sequences used in the study for gene expression downregulation | |
| --- | --- |
| Target | siRNA sequence 5´-3´ |
| Scramble (Luc) | GUAAGACACGACUUAUCGC |
| *NOX4* | GCCUCUACAUAUGCAAUAA |
| *NOX1* | ACAAGCUGGUGGCCUAUAU |

| **Supplementary Table 2.** Mouse primers used for quantitative real-time PCR. | | | | |
| --- | --- | --- | --- | --- |
| Gene | Protein | Forward | Reverse |  |
| *Acta2* | α-Sma | TCACCATTGGAAACGAACGC | CCCCTGACAGGACGTTGTTA |  |
| *Col1a1* | Collagen 1 | GAAACCCGAGGTATGCTTGA | GACCAGGAGGACCAGGAAGT |  |
| *Krt19* | Keratin 19 | CCCTCCCGAGATTACAACCA | GGCGAGCATTGTCAATCTGT |  |
| *Rpl32* | L32 | ACAATGTCAAGGAGCTGGAG | TTGGGATTGGTGACTCTGATG |  |
| *Smad7* | Smad7 | GTGTTGCTGTGAATCTTACGG | ATTGGGTATCTGGAGTAAGGAG |  |
| *Tgfb1* | Tgfb1 | GTCAGACATTCGGGAAGCAG | GCGTATCAGTGGGGGTCA |  |
| *Tgfb2* | Tgfb2 | TCCCCTCCGAAAATGCCATC | GGAAGACCCTGAACTCTGCC |  |
| *Tgfb3* | Tgfb3 | TTTGCGGAGGACGGAGTAAC | ACAGTCACCAGCATCTCAGC |  |
| *Tgfbr1* | Tgfbr1 | CCAAACCACAGAGTAGGCAC | ACCAATAGAACAGCGTCGAG |  |
| *Tgfbr2* | Tgfbr2 | GGAGAAGTGAAGGATTACGAGC | CACACGATCTGGATGCCC |  |
| *Tgfbr3* | Tgfbr3 | GGTGTGAACTGTCACCGATCA | GTTTAGGATGTGAACCTCCCTTG |  |

| **Supplementary Table 3.** Human primers used for quantitative real-time PCR. | | | |
| --- | --- | --- | --- |
| Gene | Protein | Forward | Reverse |
| *ACTA2* | Α-SMA | AGAGTTACGAGTTGCCTGATG | GCTGTTGTAGGTGGTTTCATG |
| *BMF* | BMF | CAAATCTGAACAAGCCCAAGTCTTCCAG | CACACAGCTTAGTGAGCAGAACACAA |
| *CCDC80* | CCDC80 | CCAGAGAAGGAGGAGTGTGC | GGGCGAGCTAGTCTCAACAC |
| *CDKN1A* | p21 | CCAGCATGACAGATTTCTACCAC | GATGTAGAGCGGGCCTTTGA |
| *COL1A1* | Collagen 1 | CCCCTGGAAAGAATGGAGATG | TCCAAACCACTGAAACCTCTG |
| *CYBA* | p22phox | TCCTGCATCTCCTGCTCTC | CACAGCCGCCAGTAGGTAG |
| *CYBB* | NOX2 | AGGAGTTTCAAGATGCGTGG | TTGAGAATGGATGCGAAGGG |
| *DUOX1* | DUOX1 | CCTGCCTAAGGAACACACTC | GGGGCTCAGGGATTAGAATGTC |
| *DUOX2* | DUOX2 | CAACCCTAATGTGGACCCCC | GTCGAGGACAATGGCACTGA |
| *FRMD6* | FRMD6 | TGAAAACCTGCAGCTCAATG | CTGGGCTGACTTCCAGAGAC |
| *GADD45B* | GADD45B | TACGAGTCGGCCAAGTTGATG | GGATGAGCGTGAAGTGGATTT |
| *GAPDH* | GAPDH | AGCCACATCGCTCAGACAC | GCCCAATACGACCAAATCC |
| *KRT19* | CK-19 | GCGAGCTAGAGGTGAAGATC | AATCCTGGAGTTCTCAATGGTG |
| *NOX1* | NOX1 | GCCTTGATTCTCATGGTAACTTCAGCTAC | ATTCATGCTCTCCTCTGTTTGACCC |
| *NOX3* | NOX3 | ATGGGACGGGTCGGATTGTT | ATCCATTTCCAAGCCGAGGGTTC |
| *NOX4* | NOX4 | GCAGGAGAACCAGGAGATTG | CACTGAGAAGTTGAGGGCATT |
| *NOX5* | NOX5 | CTGTATAACCAAGGGCCAGAG | AGGTAAGCCAAGAGTGTTCG |
| *NOXA1* | NOXA1 | GGCTTCTTCCAGCGAGGAGT | CATTGTGTAGCACCTCCCAGGC |
| *NOXO1* | NOXO1 | GTGCAGATCAAGAGGCTCCAAACG | CTTTGGGAGAACGCGGTCAGAT |
| *RAC1* | RAC1 | GCTTTTCCCTTGTGAGTCCTG | CCTTCAGTTTCTCGATCGTGTC |
| *SMAD7* | SMAD7 | CGGACAACAAGAGTCAGCTGGT | GTCCTGGAGTCCGGGTTGTC |
| *TGFB1* | TGFB1 | AAGTGGACATCAACGGGTTC | GTCCTTGCGGAAGTCAATGT |
| *TGFB2* | TGFB2 | TACTACGCCAAGGAGGTTTACAAA | TTGTTCAGGCACTCTGGCTTT |
| *TGFB3* | TGFB3 | CTGGATTGTGGTTCCATGCA | TCCCCGAATGCCTCACAT |
| *TGFBR1* | TGFBR1 | ACATGATTCAGCCACAGATACC | GCATAGATGTCAGCACGTTTG |
| *TGFBR2* | TGFBR2 | CTGTGGATGACCTGGCTAAC | CATTTCCCAGAGCACCAGAG |
| *TGFBR3* | TGFBR3 | TGGGGTCTCCAGACTGTTTTT | CTGCTCCATACTCTTTTCGGG |
| *VDR* | VDR | ATCGGCATGATGAAGGAGTT | TGCTCCTCAGACAGCTTGG |

| **Supplementary Table 4**. Primary antibodies used for immunodetection. | | | | | | | |
| --- | --- | --- | --- | --- | --- | --- | --- |
| **Protein** | **Antibody** | **Company** | **Type** | | **Source** | **Use** | **Dilution** |
| α-SMA | ab5694 | Abcam | | Polyclonal | Rabbit | IHC | 1/2000 |
|  |  |  |  |  |  | WB | 1/2000 |
| β-actin | A5441 | Sigma-Aldrich | | Monoclonal | Mouse | WB | 1/5000 |
| CD4 | CST 25229 | CST | | Monoclonal | Rabbit | IHC | 1/100 |
| CD20 | ab64088 | Abcam | | Monoclonal | Rabbit | IHC | 1/100 |
| CK19 | ab133496 | Abcam | | Monoclonal | Rabbit | IHC | 1/4000 |
| PanCK | PDM072 | PALEX | | Monoclonal | Mouse | IHC | 1/300 |
| Cleaved Caspase 3 | CST 9661 | CST | | Polyclonal | Rabbit | IHC | 1/100 |
| Cleaved Parp | CST 5625 | CST | | Monoclonal | Rabbit | WB | 1/1000 |
| COL1 | PA2140-2 | Boster Biological Technology | | Polyclonal | Rabbit | WB | 1/2000 |
| Cyclin D1 | sc-718 | Santa Cruz Biotechnology | | Polyclonal | Rabbit | WB | 1/1000 |
| F4/80 | CST 70076 | CST | | Monoclonal | Rabbit | IHC | 1:200 |
| Ki67 | IHC-00375 | Thermo Fisher Scientific | | Polyclonal | Rabbit | IHC | 1:100 |
| Ki67 | ab16667 | Abcam | | Monoclonal | Rabbit | IF | 1/500 |
| Ki67 | 790-4286 | ROCHE | | Monoclonal | Rabbit | IHC | 1/300 |
| NOX4 | A00403 | BOSTER | | Polyclonal | Rabbit | WB | 1/1000 |
| NOX4 | ----- | Doroshow lab | | Monoclonal | Rabbit | IHC | 1/100 |
| NOX1 | ab55831 | Abcam | | Polyclonal | Rabbit | WB | 1/500 |
| NOX1 | ----- | Doroshow lab | | Monoclonal | Mouse | IHC | 1/50 |
| PD-L1 | CST 64988 | CST | | Monoclonal | Rabbit | IHC | 1/100 |
| Perforin | CST 31647 | CST | | Monoclonal | Rabbit | IHC | 1/100 |
| p22phox | TA334647 | ORIGENE | | Polyclonal | Rabbit | WB | 1/500 |
| pSMAD2 | CST 3108 | CST | | Monoclonal | Rabbit | WB | 1/1000 |
| SMAD2 | CST 3103 | CST | | Monoclonal | Mouse | WB | 1/1000 |
| pSMAD3 | 07-1389 | Millipore | | Polyclonal | Rabbit | WB | 1/1000 |
| SMAD3 | 04-1035 | Millipore | | Monoclonal | Rabbit | WB | 1/1000 |
| SMAD4 | CST 9515 | CST | | Polyclonal | Rabbit | WB | 1/1000 |
| SMAD7 | 25840-1-AP | Proteintech | | Polyclonal | Rabbit | WB | 1/1000 |
| pSTAT3 | CST 9145 | CST | | Monoclonal | Rabbit | WB | 1/1000 |
| STAT3 | CST 4904 | CST | | Monoclonal | Rabbit | WB | 1/500 |
| pAkt | CST 4060 | CST | | Monoclonal | Rabbit | WB | 1/1000 |
| Akt | CST 9272 | CST | | Polyclonal | Rabbit | WB | 1/1000 |
| pERK | CST 9101 | CST | | Polyclonal | Rabbit | WB | 1/1000 |
| ERK | CST 4695 | CST | | Monoclonal | Rabbit | WB | 1/1000 |
| Tubulin | T9026 | Sigma | | Monoclonal | Mouse | WB | 1/1000 |
| Vimentin | MAB2105 | R&D Systems | | Monoclonal | Rat | IHC | 1/250 |
| VISTA | CST 54979 | CST | | Monoclonal | Rabbit | IHC | 1/100 |
| WB, Western Blot; IHC, immunohistochemistry; IF, immunofluorescence | | | | | | | |

| **Supplementary Table 5. Clinical and pathological characteristics of patients with iCCA (n=17)** | |
| --- | --- |
| Age (years)  Mean (± SD) | 66.6 ± 2.5 |
| Sex ratio (M/F) | 0.41 |
| Tumor size (mm)  Mean (± SD) | 70 ± 10 |
| Histological grade  1/4  2/4  3/4  4/4 | 4/17 (23.5%)  12/17 (70.5%)  0/17 (0%)  1/17 (5.8%) |
| pT stage  T1a  T1b  T2  T3  T4 | 3/17 (17.6%)  5/17 (29.4%)  8/17 (47.1%)  1/17 (5.88%)  0/17 (0%) |

**
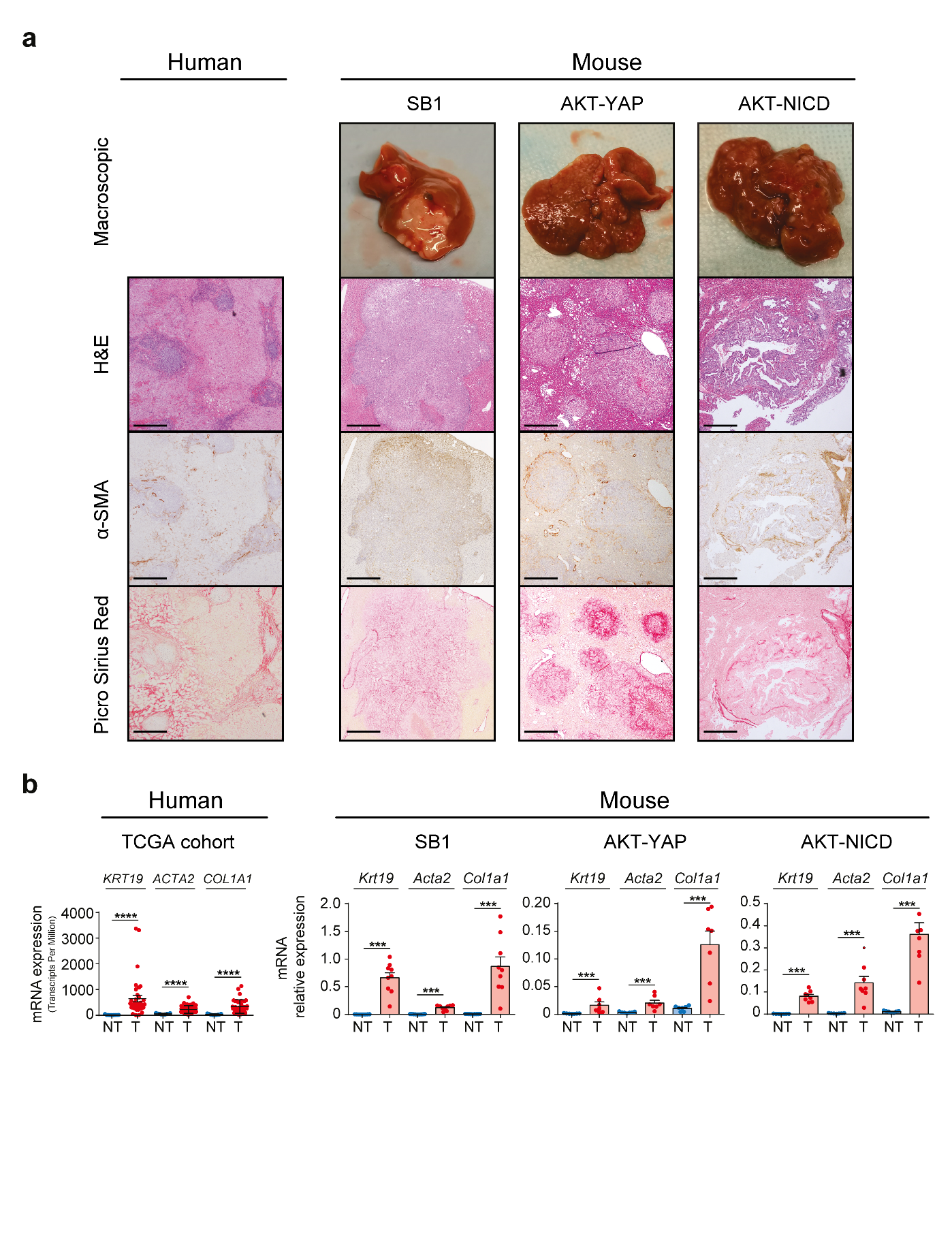
Supplementary Figures**

**Supplementary Figure 1. Intrahepatic cholangiocarcinoma (iCCA) mouse models are representative of human iCCA tumours. a.** Representative pictures of macroscopic tumours, Hematoxilin and Eosin (H&E), α-SMA and Picro Sirius Red stainings in iCCA tumours from patients and from the animal models used in this study. Scale: 500 µm. **b.** *KRT19*, *ACTA2* and *COL1A1* mRNA expression in the same settings than a (from RNAseq in the case of the TCGA cohort (n=36) and RT-qPCR in the case of mouse models (SB1 syngeneic orthotopic (n=9) and the AKT-YAP (n=7) and AKT-NICD hydrodynamic tail vein injection iCCA tumour models)). Values are expressed as means ± SEM. ***p <0.001, ****p <0.0001 as compared to NT. NT, non-tumoral; T, tumour.


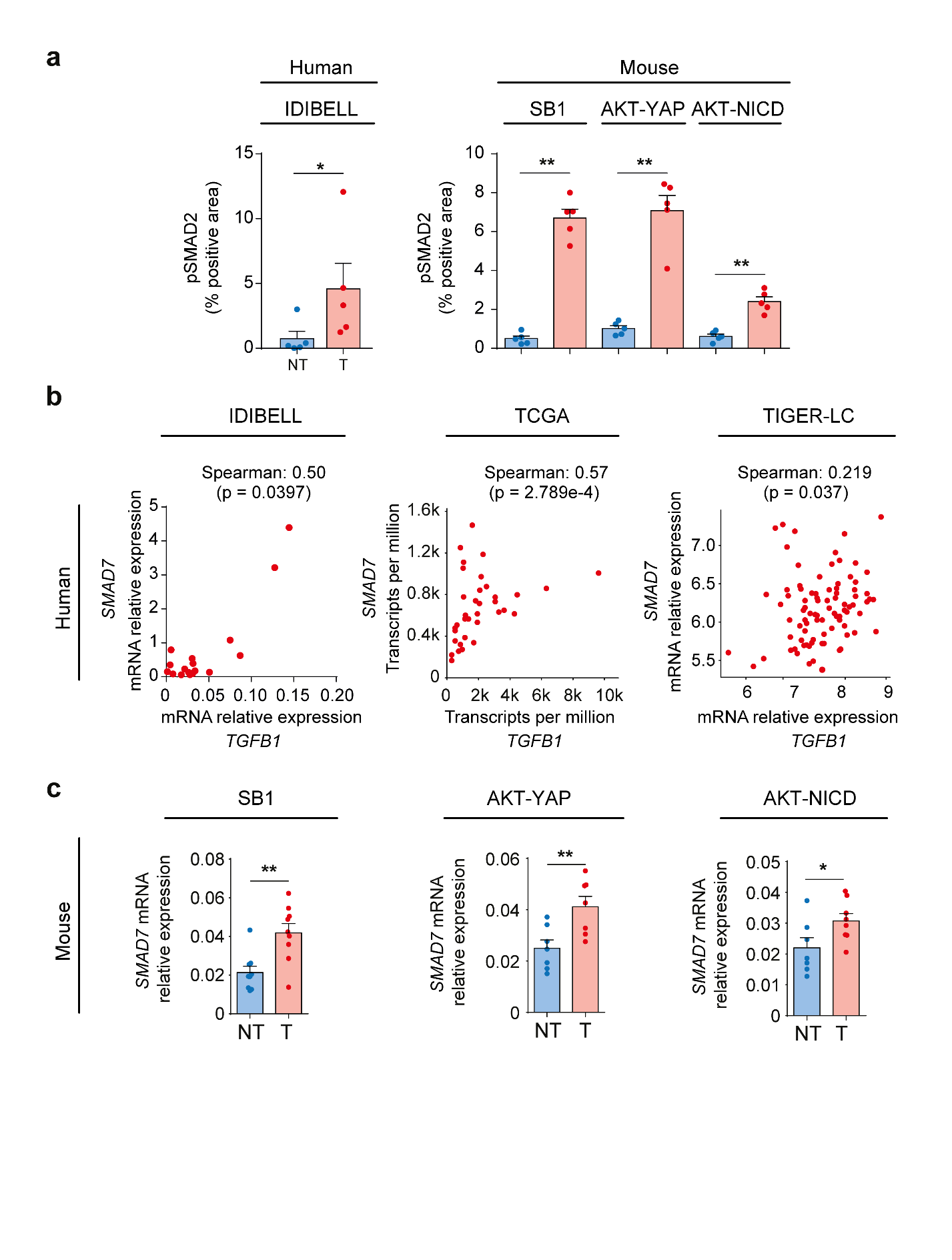


**Supplementary Figure 2. TGF-β signalling is activated and functional in human and mouse intrahepatic cholangiocarcinoma (iCCA) tumours. a**. Quantification of pSMAD2 staining iCCA tumours (T) and non-tumoral liver (NT) from 5 iCCA patients from the IDIBELL cohort and 5 animals from the SB1 syngeneic orthotopic and the AKT-YAP and AKT-NICD hydrodynamic tail vein injection iCCA tumour models. **b.** Correlation between SMAD7 and TGFB1 expression in the IDIBELL, TCGA and TIGER-LC CCA cohorts, determined using Spearman’s correlation analysis. **b**. Smad7 mRNA expression in the SB1 syngeneic orthotopic (n=9) and the AKT-YAP (n=7) and AKT-NICD (n=8) hydrodynamic tail vein injection iCCA tumour models, determined by RT-QPCR. Values are expressed as means ± SEM. *p <0.05; **p <0.01, as compared to NT. NT, non-tumoral; T, tumour.


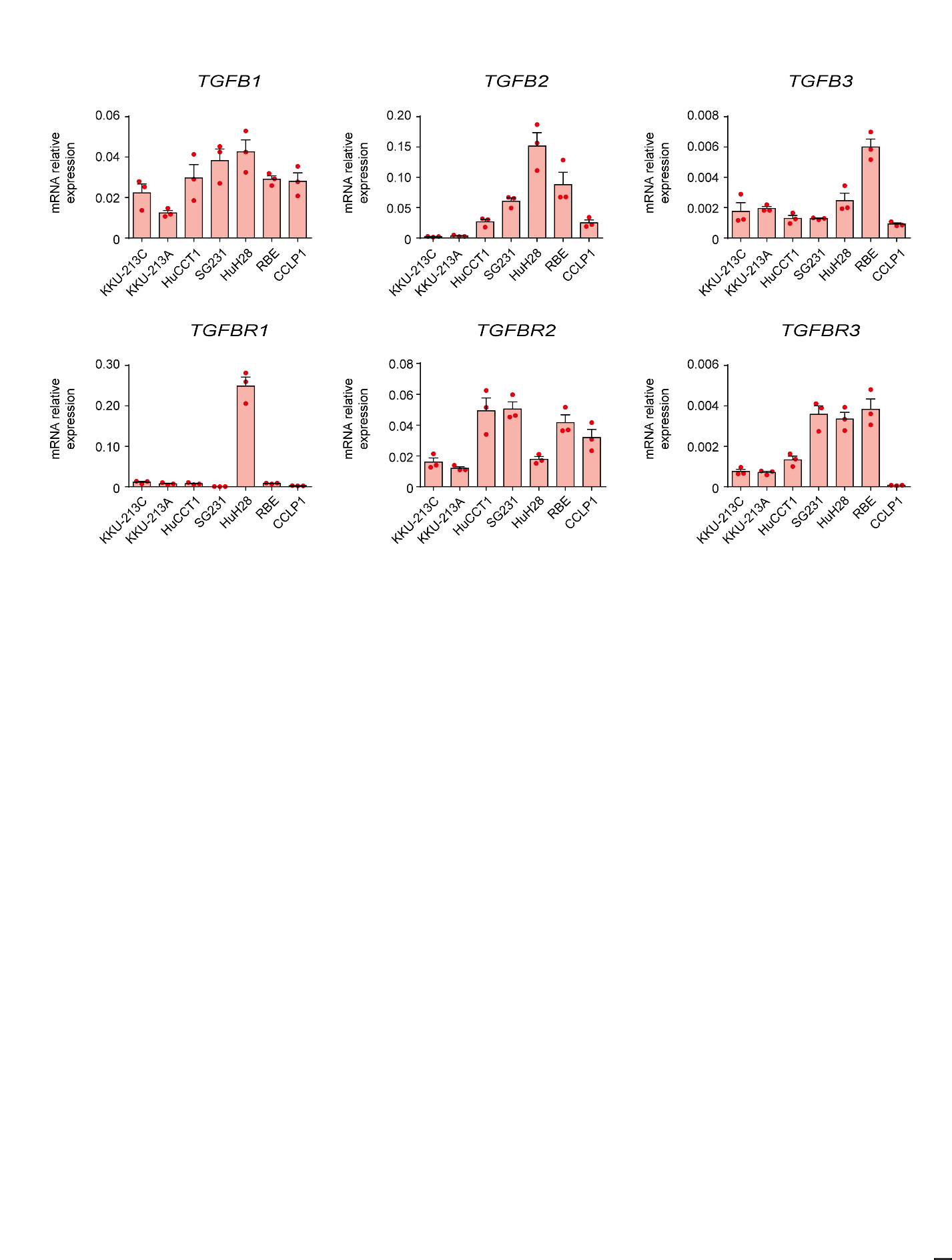


**Supplementary Figure 3. Characterization of TGF-β signalling pathway members in intrahepatic cholangiocarcinoma (iCCA) cell lines. a.** mRNA relative expression of TGF-β ligands and receptors in iCCA cell lines determined by RT-QPCR. Values are expressed as means ± SEM of at least 3 cultures.


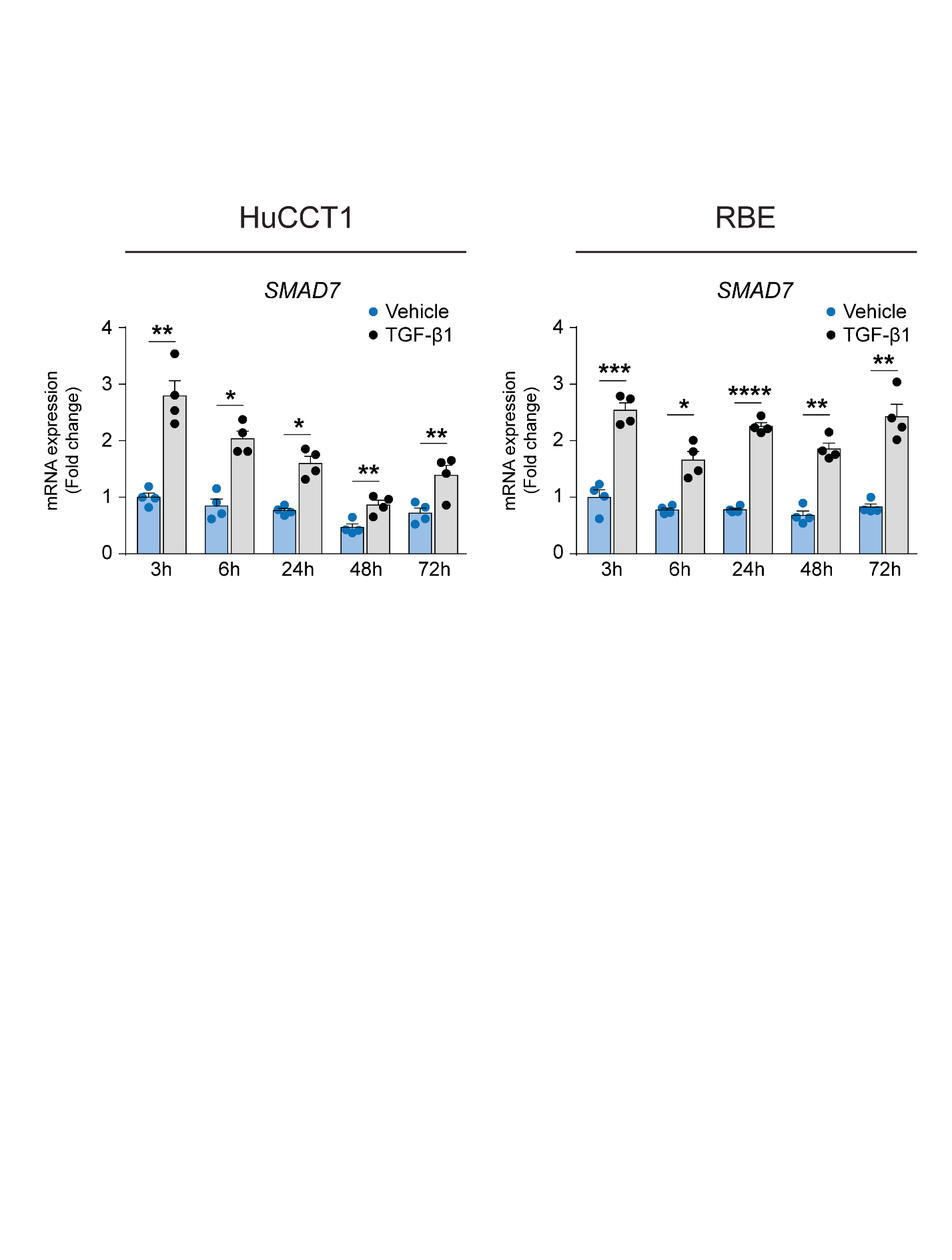


**Supplementary Figure 4. TGF-β1 regulation of SMAD7 expression in intrahepatic cholangiocarcinoma (iCCA) cell lines.** *SMAD7* mRNA expression was determined by RT-qPCR in HuCCT1 and RBE iCCA cell lines exposed to TGF-β1 (2ng/ml) for the indicated times. Values are expressed as means ± SEM from at least 3 cultures and represented as fold change versus HuCCT1 or RBE cells treated with vehicle for 3 h. *p <0.05, **p <0.01, ***p <0.001, ****p <0.0001; as compared to the vehicle.


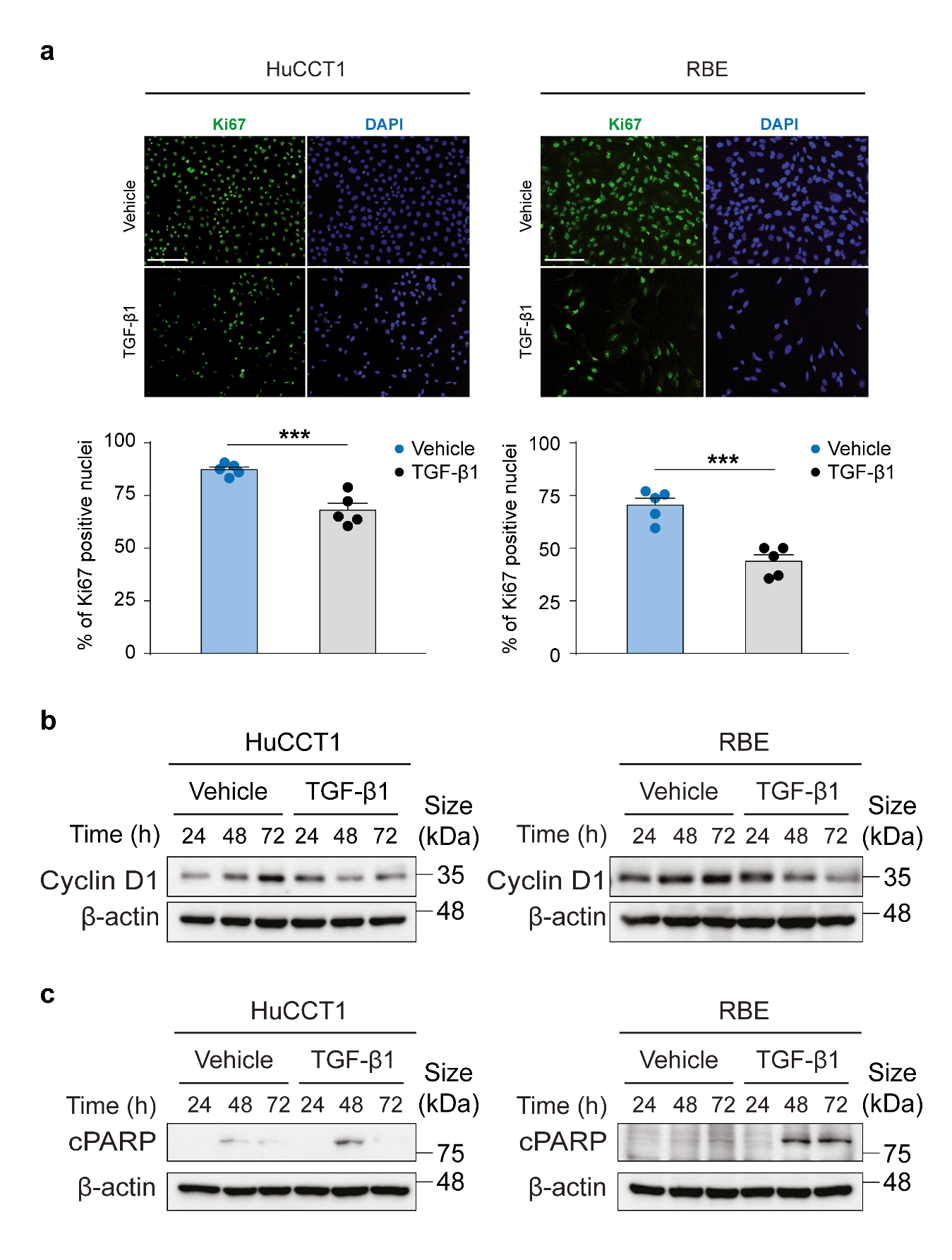


**Supplementary Figure 5. TGF-β suppressor effects involve suppression of proliferation and activation of apoptosis in intrahepatic cholangiocarcinoma (iCCA) cell lines. a.** Effect of TGF-β1 on the proliferation of HuCCT1 and RBE cells. Cell proliferation was ascertained by counting Ki67 positive nuclei (green) after incubation with TGF-β1 (2 ng/ml) for 72h. Scale: 100 µm.Values are expressed as means ± SEM from at least 3 cultures. **, p < 0.01; ***, p < 0.001; as compared with untreated cells. **b-c.** Representative images of Western blot analysis of Cyclin D1 (b) and cleaved PARP (c) in HuCCT1 and RBE iCCA cells after treatment with TGF-β1 during the indicated time periods.

**
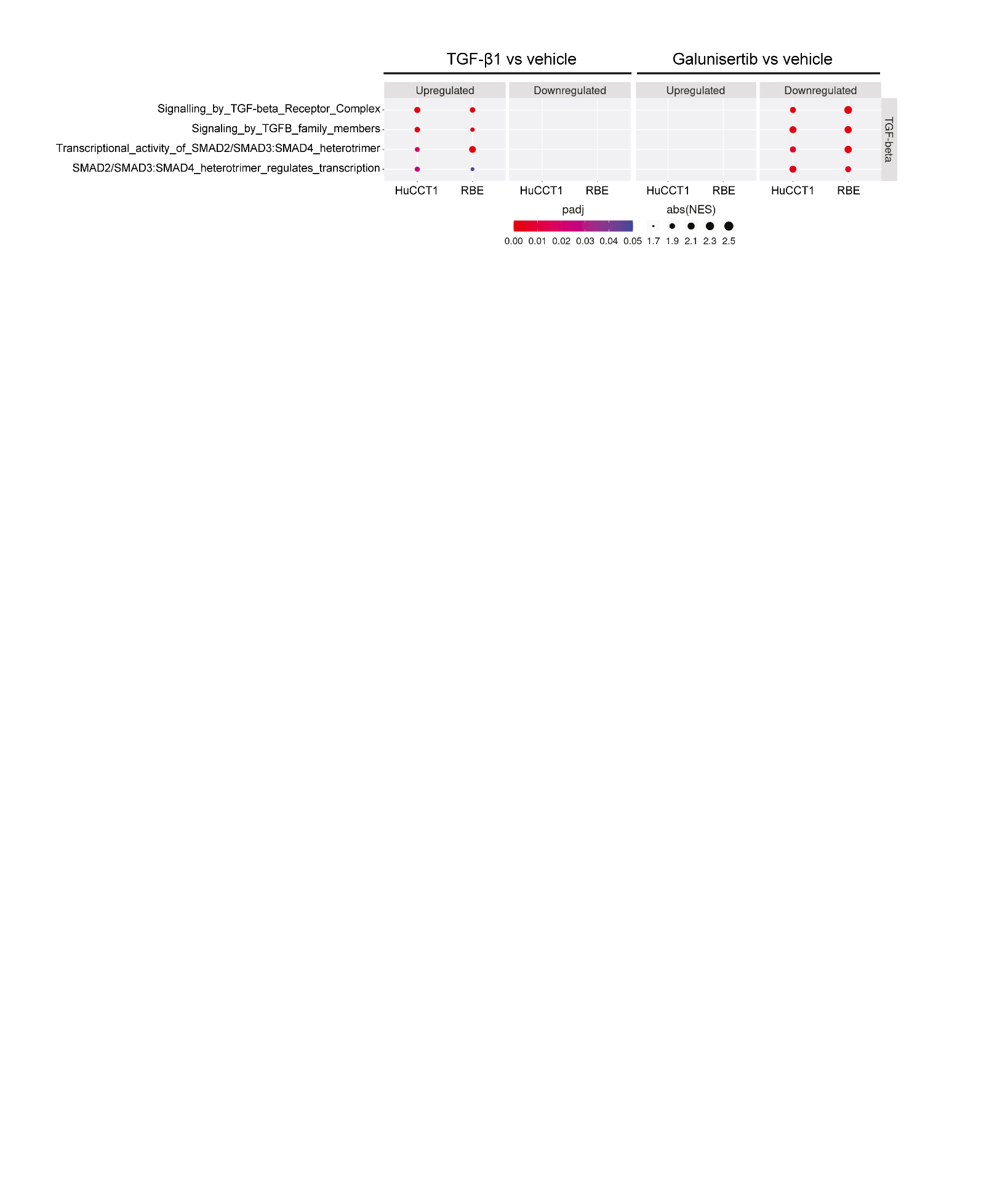
**

**Supplementary Figure 6. TGF-β and galunisertib regulate pathways in intrahepatic cholangiocarcinoma (iCCA) cell lines.** Dot plot showing differences in enrichment for TGF-β signalling pathways in iCCA cell lines treated with TGF-β1 or TGF-β receptor I inhibitor galunisertib. Data obtained from RNAseq analysis.


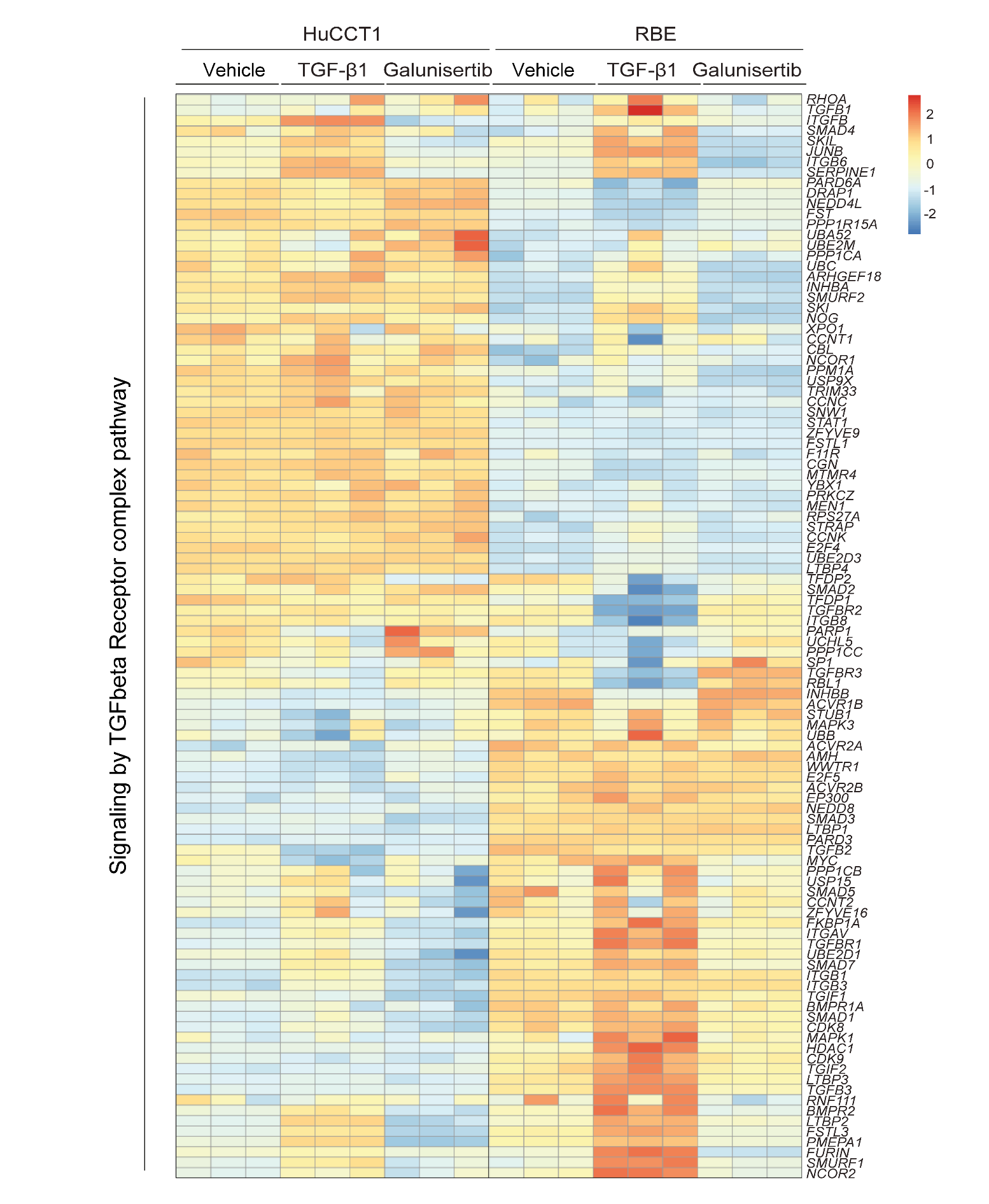


**Supplementary Figure 7. TGF-β and galunisertib regulate pathways in intrahepatic cholangiocarcinoma (iCCA) cell lines.** Heatmaps showing changes in the expression of genes from the “Signalling by TGFbeta Receptor complex” pathway. Data obtained from RNAseq analysis.


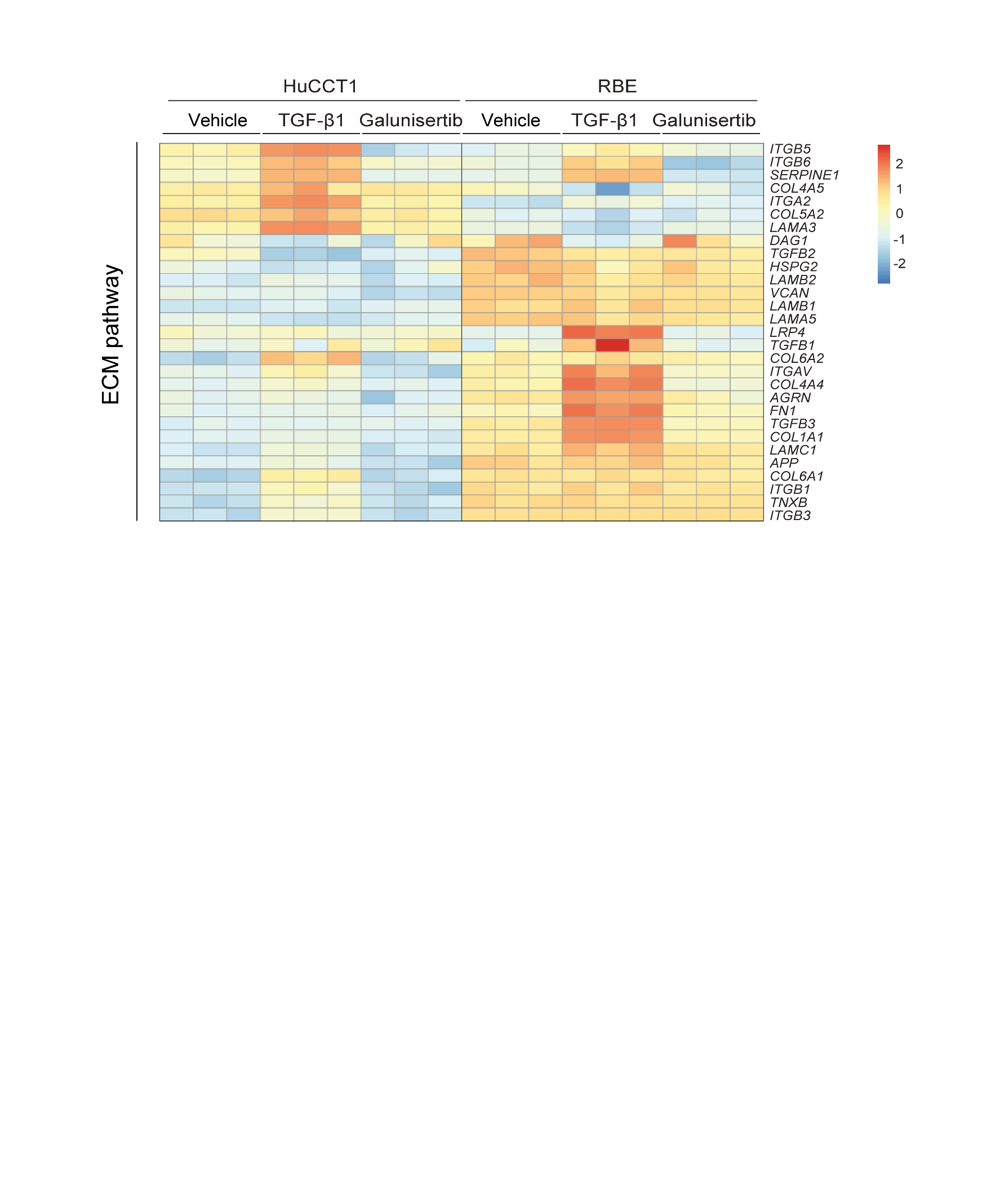


**Supplementary Figure 8. TGF-β and galunisertib regulate pathways in intrahepatic cholangiocarcinoma (iCCA) cell lines.** **a-c.** Heatmaps showing changes in the expression of genes from the extracellular matrix “ECM” pathway. Data obtained from RNAseq analysis.


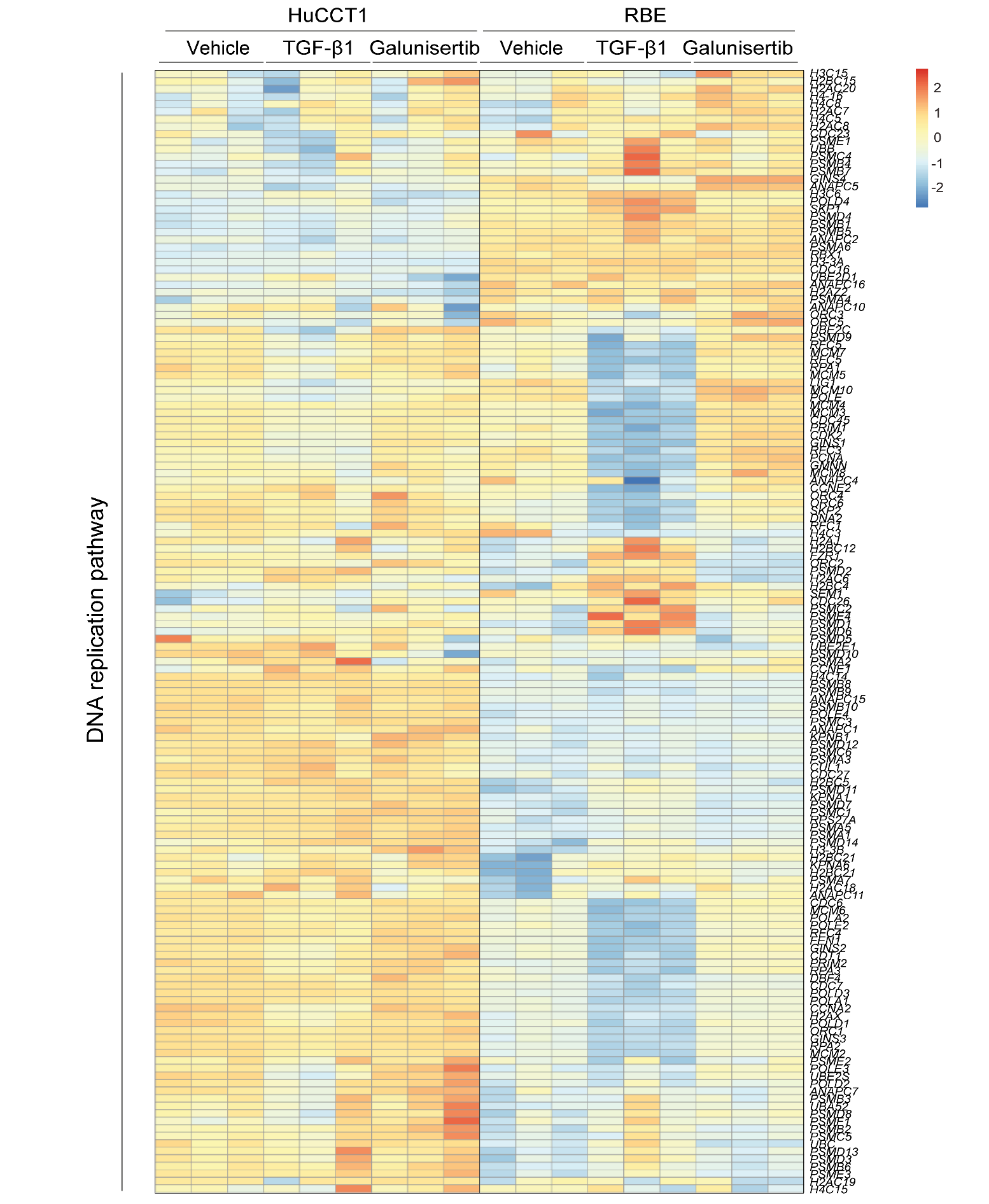


**Supplementary Figure 9. TGF-β and galunisertib regulate pathways in intrahepatic cholangiocarcinoma (iCCA) cell lines.** **a-c.** Heatmaps showing changes in the expression of genes from the “DNA replication” pathway. Data obtained from RNAseq analysis.


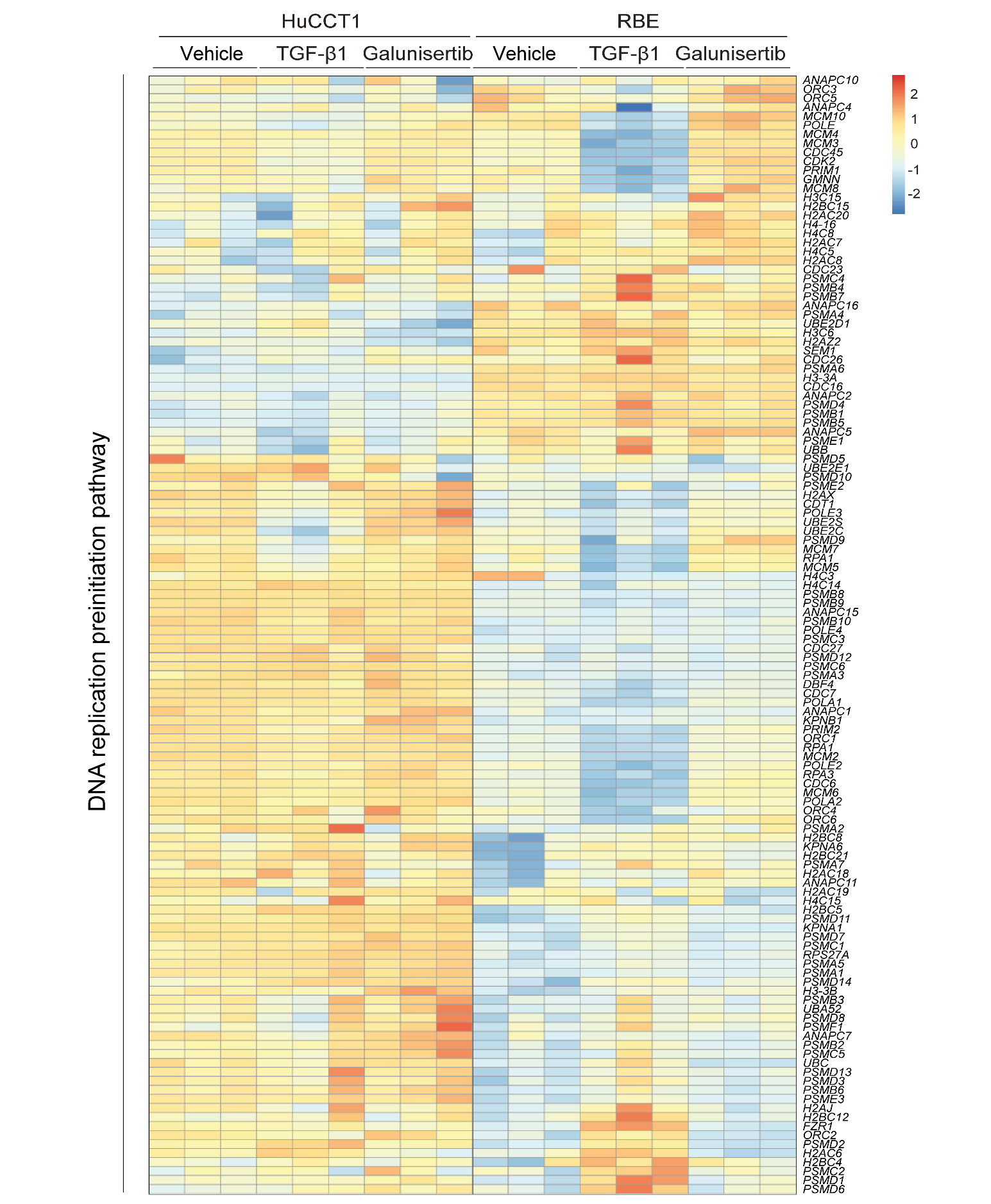


**Supplementary Figure 10. TGF-β and galunisertib regulate pathways in intrahepatic cholangiocarcinoma (iCCA) cell lines.** **a-c.** Heatmaps showing changes in the expression of genes from the “DNA replication preinitiation” pathway. Data obtained from RNAseq analysis.


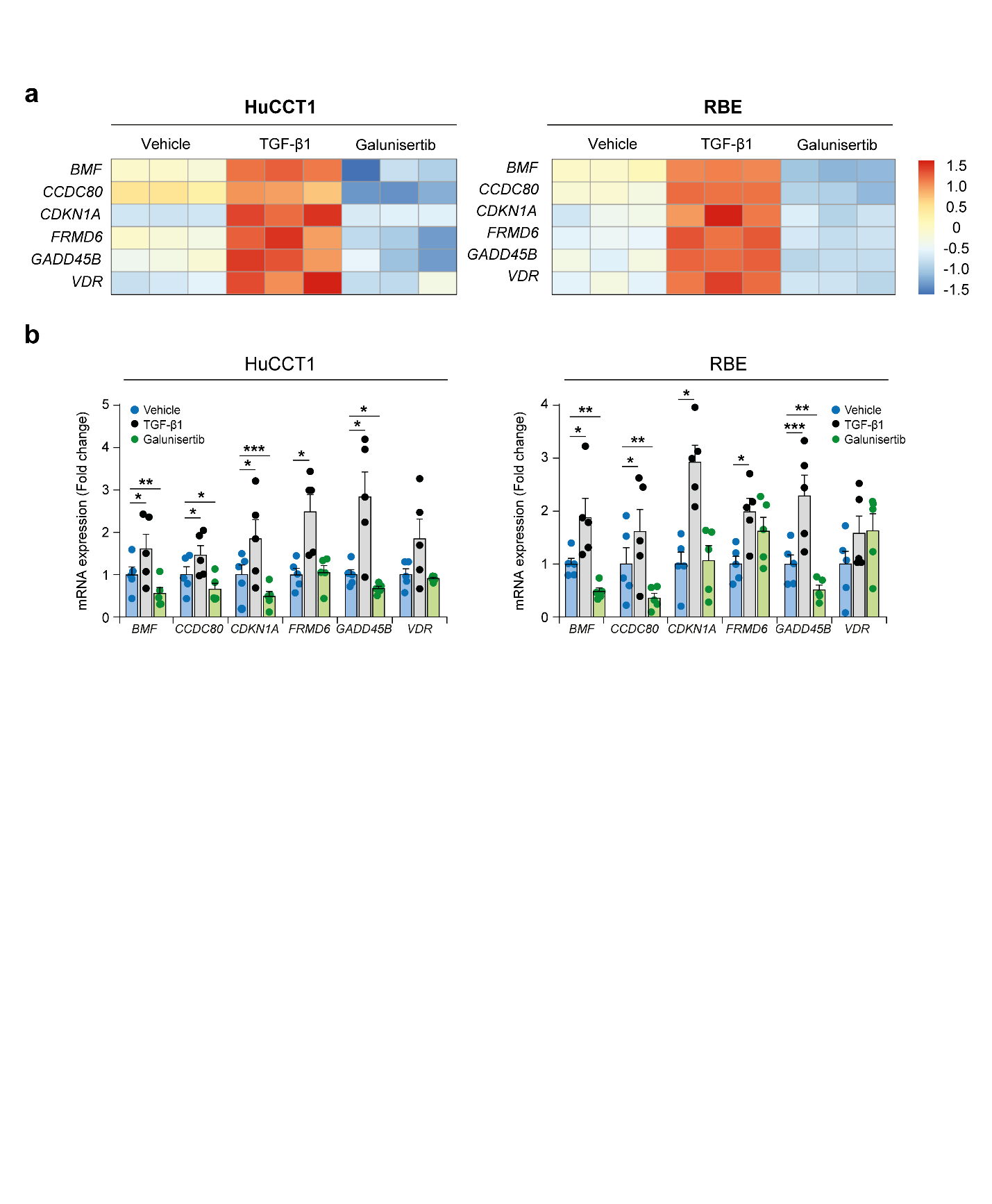


**Supplementary Figure 11. TGF-β and galunisertib regulate the expression of genes related with TGF-β suppressor effects in intrahepatic cholangiocarcinoma (iCCA) cell lines. a.** Heatmaps showing changes in *BMF*, *CCDC80*, *CDKN1A*, *FRMD6*, *GADD45B* and *VDR* expression in iCCA cell lines treated with TGF-β1 or TGF-β receptor I inhibitor galunisertib determined by RNAseq. **b.** *BMF*, *CCDC80*, *CDKN1A*, *FRMD6*, *GADD45B* and *VDR* mRNA expression in HuCCT1 and RBE 3D spheroids from Figure 3c, determined by RT-QPCR and represented as fold change *versus* vehicle. Values are expressed as means ± SEM from at least 3 cultures. *p <0.05; **p <0.01, ***p <0.001; as compared to the vehicle.

**
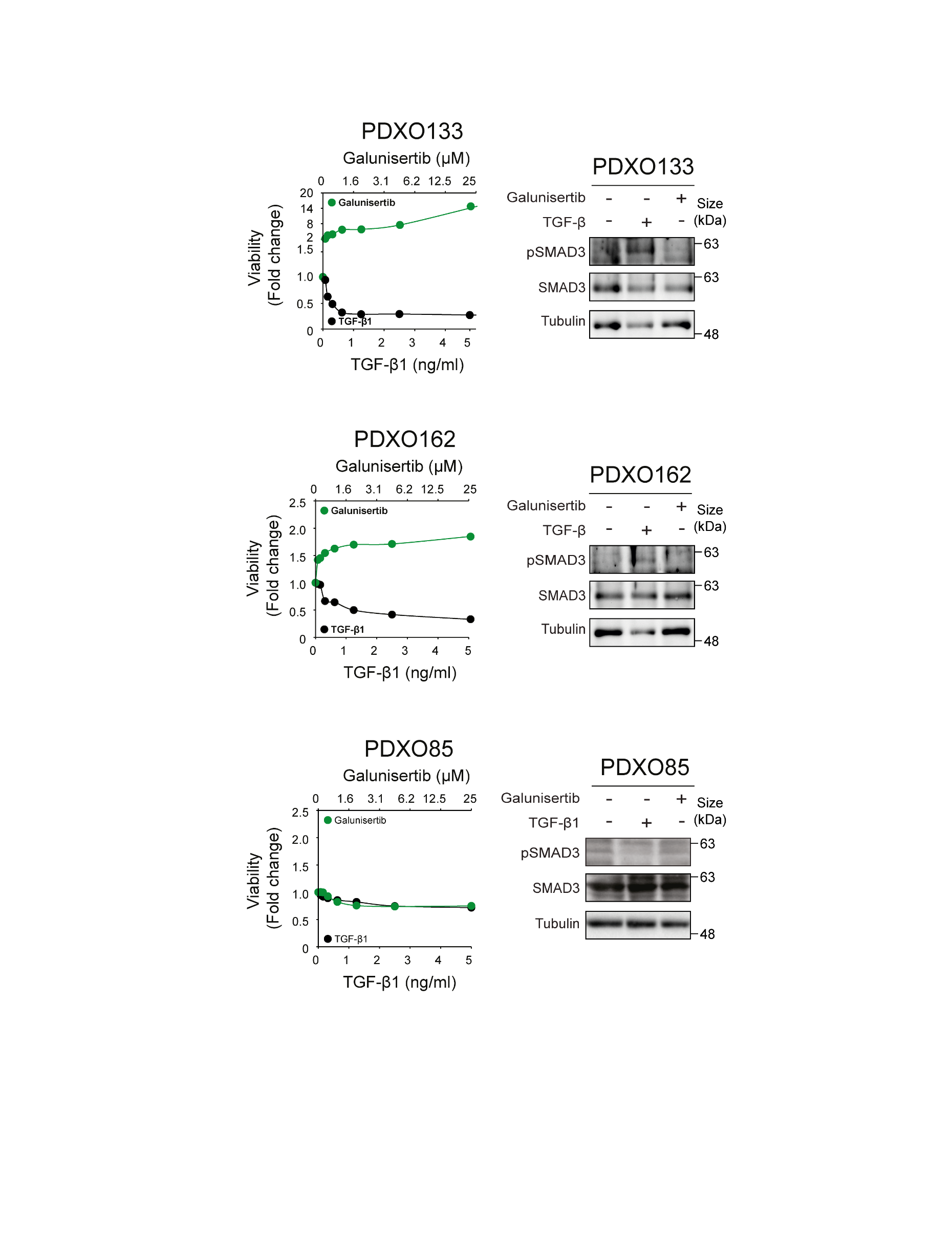
**

**Supplementary Figure 12. Galunisertib promote intrahepatic cholangiocarcinoma (iCCA) growth in patient derived organoids.** Dose-response curves of TGF-β1 and galunisertib for organoids (PDXO) from PDX133, PDX162 and PDXO85. Cell viability was determined using a Cell Titer-Glo assay 4 days after the treatment initiation. Representative images of Western blot analysis of phosphoSMAD3 and total SMAD3 in PDX133, PDX162 and PDXO85 after treating them with TGF-β1 (2ng/ml) and galunisertib (10 µM) for 4 days.


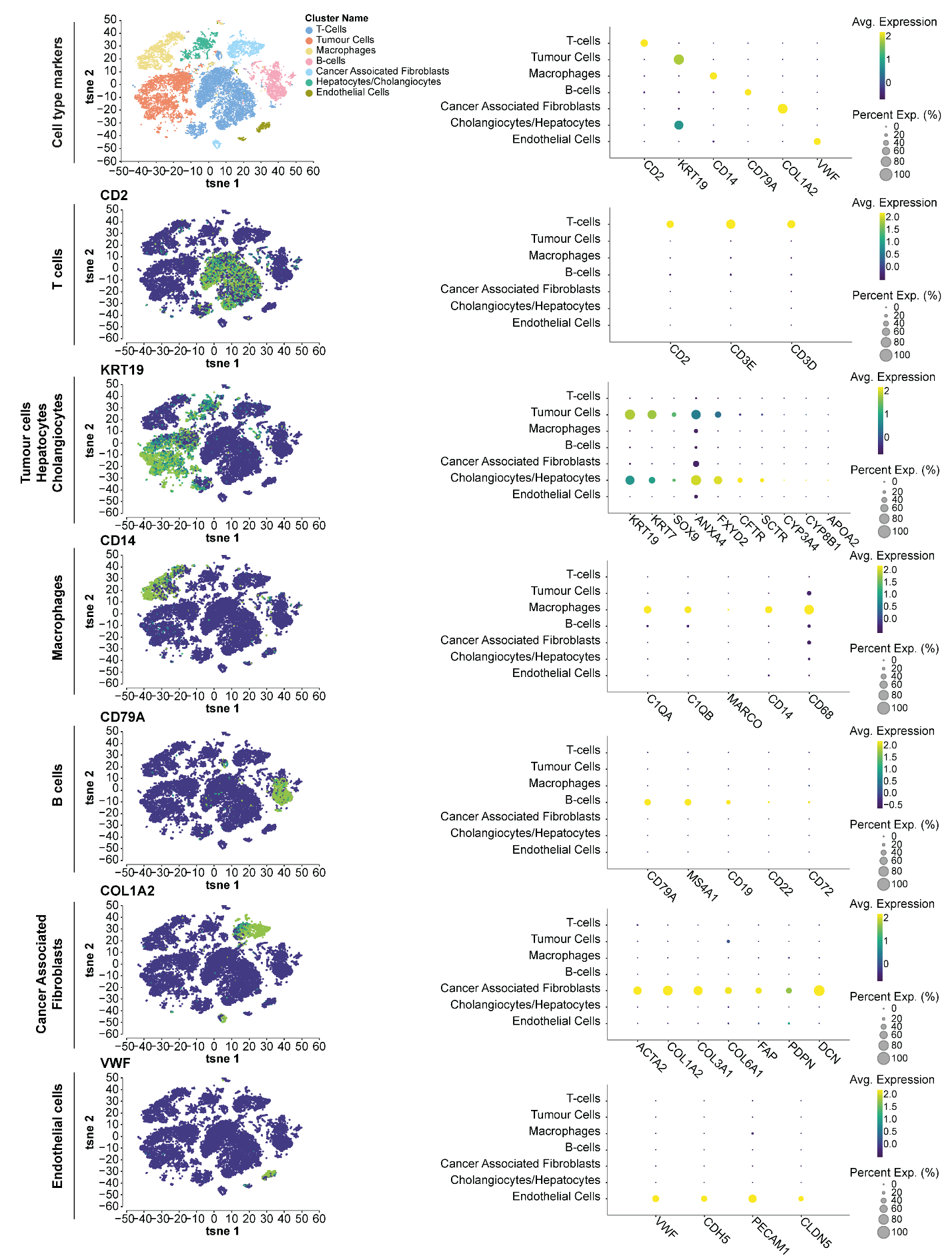


**Supplementary Figure 13.** T-SNE and dotplot showing the expression of specific markers of different cell types inside CCA tumours in scRNAseq data set GSE201425.


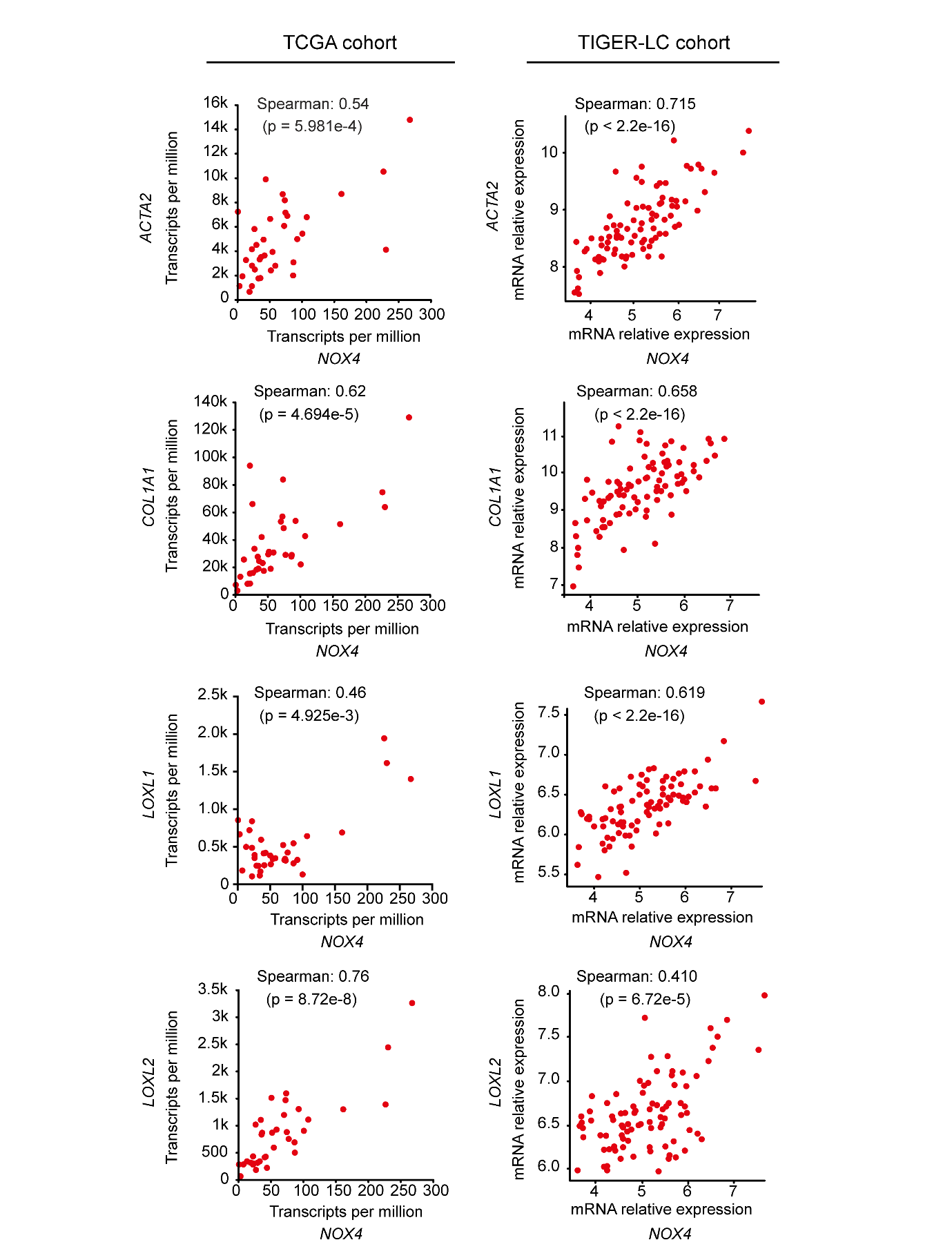


**Supplementary Figure 14. NOX4 expression correlates with the expression of cancer associated fibroblasts (CAF) markers in intrahepatic cholangiocarcinoma (iCCA) patients.** Correlation between the mRNA expression levels of *NOX4* and CAF markers *COL1A1*, *LOXL1* and *LOXL2* in the TCGA (n=36) (data from RNAseq) and TIGER-LC (n=91) (data from microarrays) CCA cohorts determined using Spearman’s correlation analysis. Data from these data bases were obtained by RNAseq and microarray analysis, respectively.

**
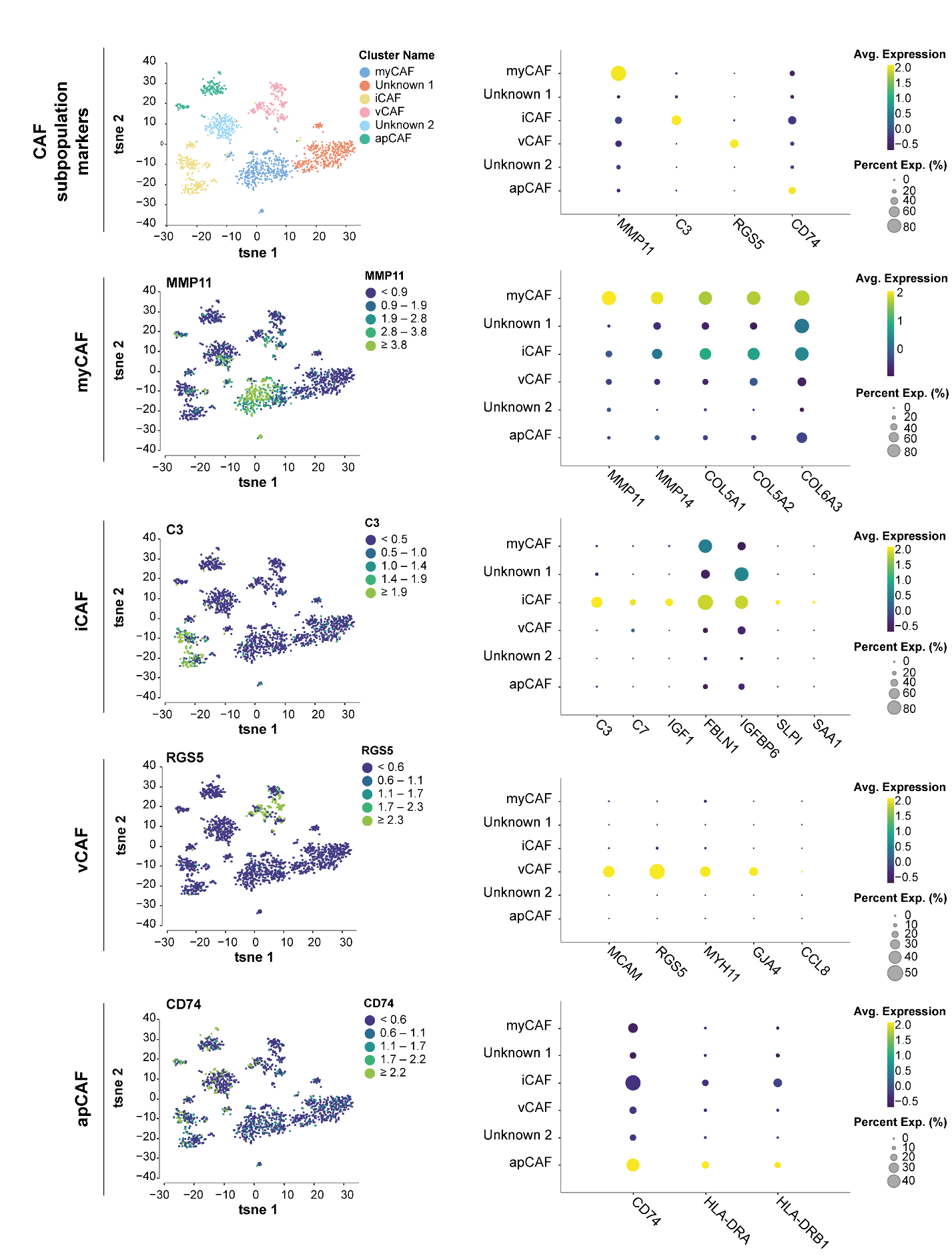
**

**Supplementary Figure 15.** T-SNE and dotplot showing the expression of specific markers of different CAF subpopulations inside CCA tumours in scRNAseq data set GSE201425.

**
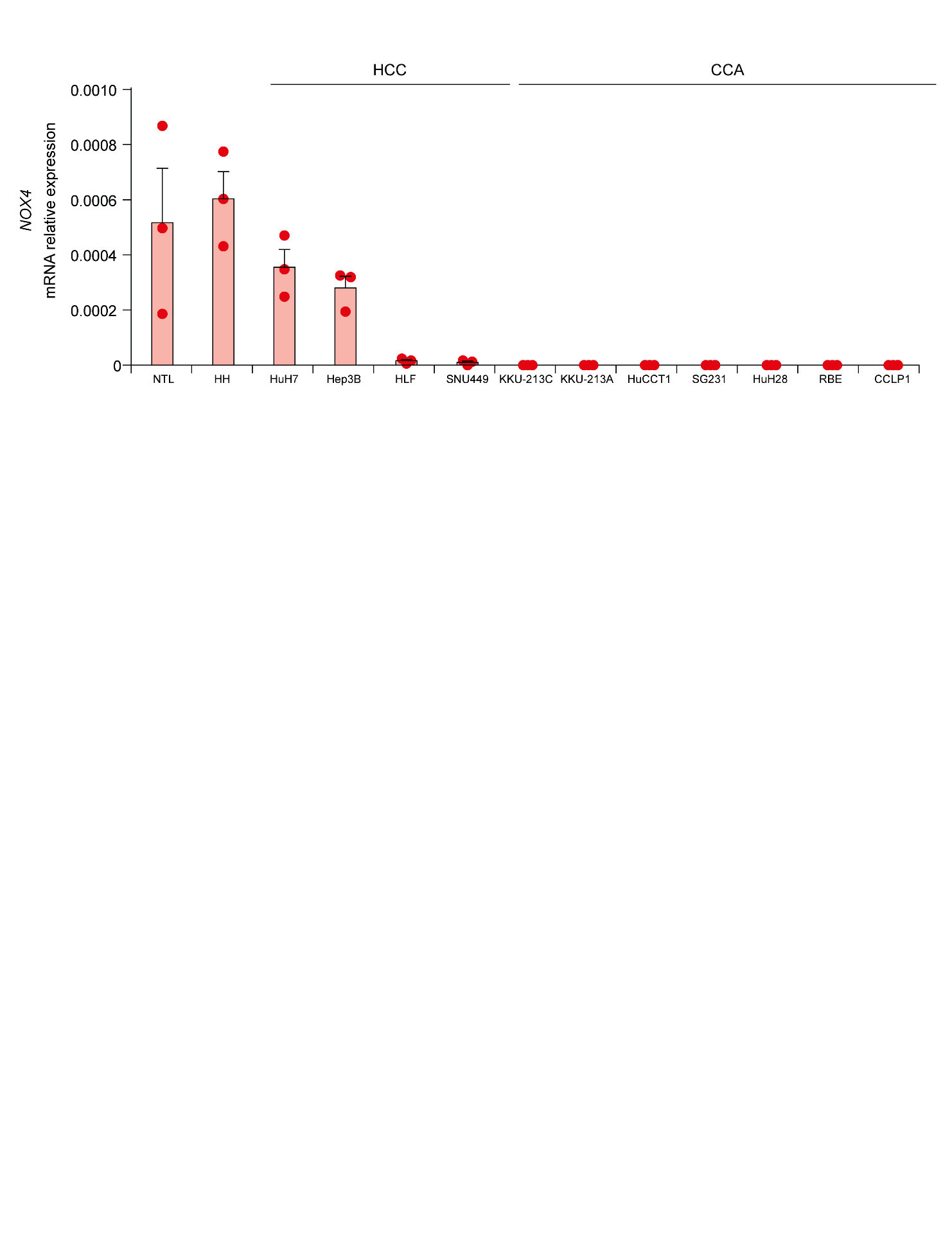
**

**Supplementary Figure 16. NOX4 is undetectable in human intrahepatic cholangiocarcinoma (iCCA) cell lines.** *NOX4* mRNA expression in non-tumoral liver (NTL), human hepatocytes (HH), four hepatocellular carcinoma (HCC) cell lines and seven iCCA cell lines. mRNA levels were determined by RT-QPCR. Results are expressed as means ± SEM from at least 3 independent cultures.


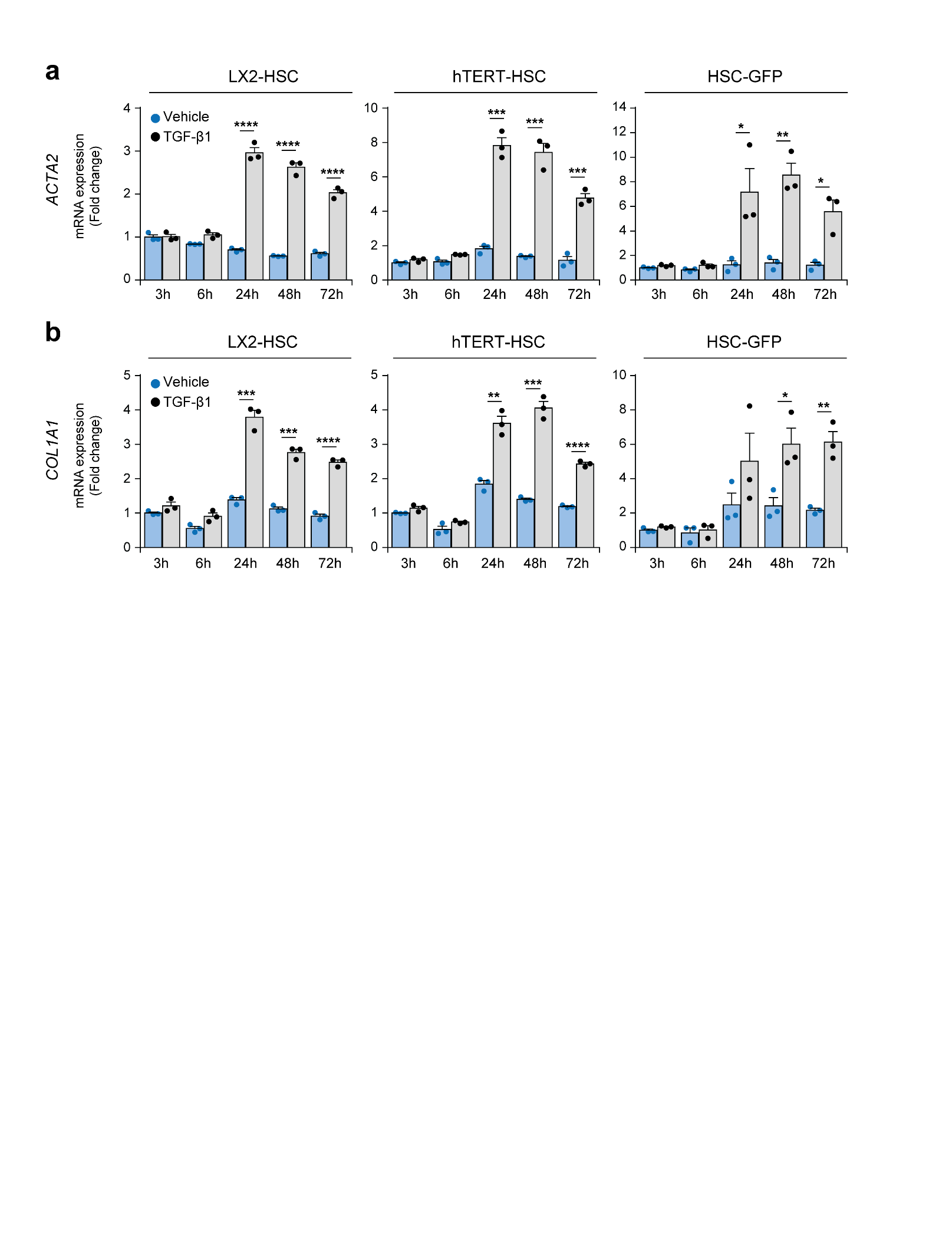


**Supplementary Figure 17. TGF-β increases de expression of *ACTA2* and *COL1A1* in hepatic stellate cells (HSC). a-b.** *ACTA2* (a) and *COL1A1* (b) mRNA expression in LX2-HSC, hTERT-HSC and HSC-GFP cells after exposure to TGF-β1 (2ng/ml) for the indicated times. mRNA levels were determined by RT-QPCR and represented as fold change *versus* the condition vehicle 3h. Values are expressed as means ± SEM from at least 3 cultures. *p <0.05, **p <0.01, ***p <0.001, ****p <0.0001; as compared to the vehicle.


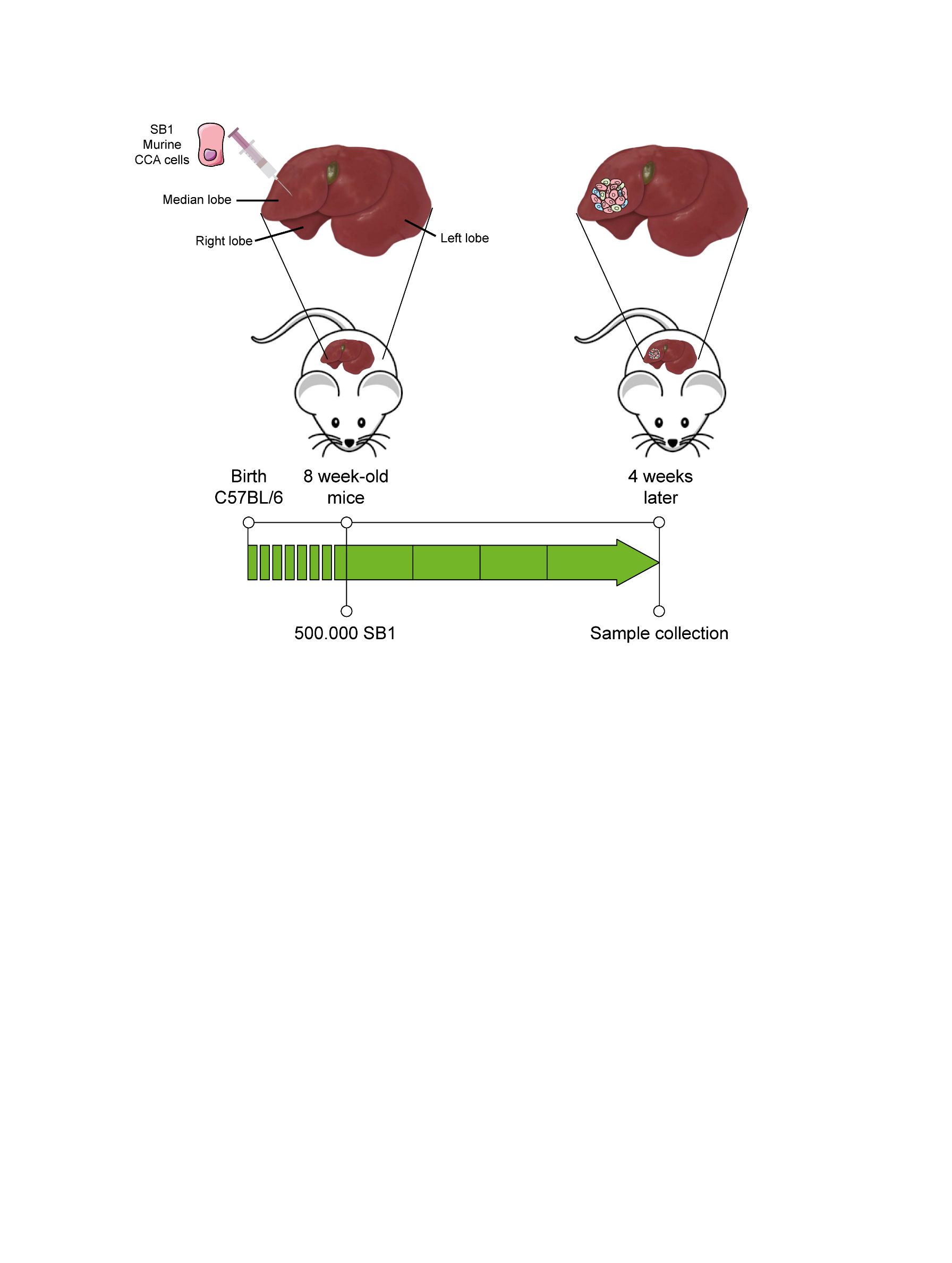


**Supplementary Figure 18.** Schematic representation of the experimental procedure to generate syngeneic orthotopic iCCA tumours in WT and NOX4^-/-^ mice.

**
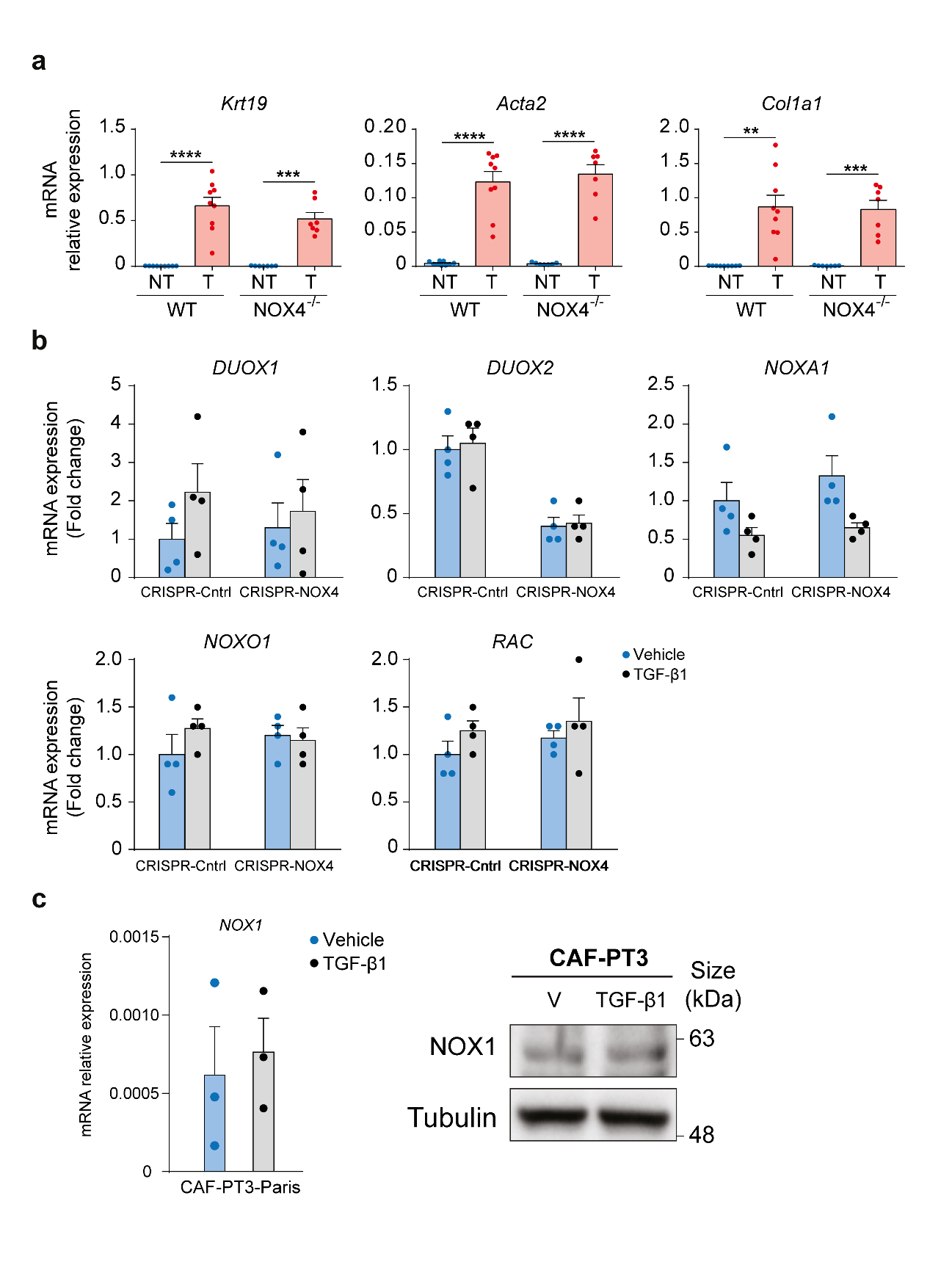
**

**Supplementary Figure 19. Expression analysis of genes in tumours derived from the SB1 syngeneic orthotopic model of intrahepatic cholangiocarcinoma (iCCA) and in hTERT-CRISPR cells. a.** *Krt19*, *Acta2* and *Col1a1* mRNA expression determined by RT-qPCR in iCCA tumours from the syngeneic orthotopic SB1 model developed in WT and NOX4^-/-^ mice comparing tumour (T) *versus* surrounding non-tumoral tissue (NT). Values are expressed as means ± SEM of at least 7 animals. Student’s t-test; **p<0.01; ***p<0.001; ****p<0.0001. **b.** Changes in mRNA expression of *DUOX1*, *DUOX2*, *NOXA1*, *NOXO1* and *RAC* after TGF-β1 (2 ng/ml) treatment for 48h in the CRISPR-NOX4 and control cells, determined by RT-QPCR and represented as fold change versus CRISPR-Cntrl. mRNA expression of *NOX2*, *NOX3* and *NOX5* were analysed but not detected. **c.** NOX1 mRNA and protein expression in CAF after exposure to TGF-β1 (2ng/ml) for 48 h. Values are expressed as means ± SEM from at least 3 cultures.


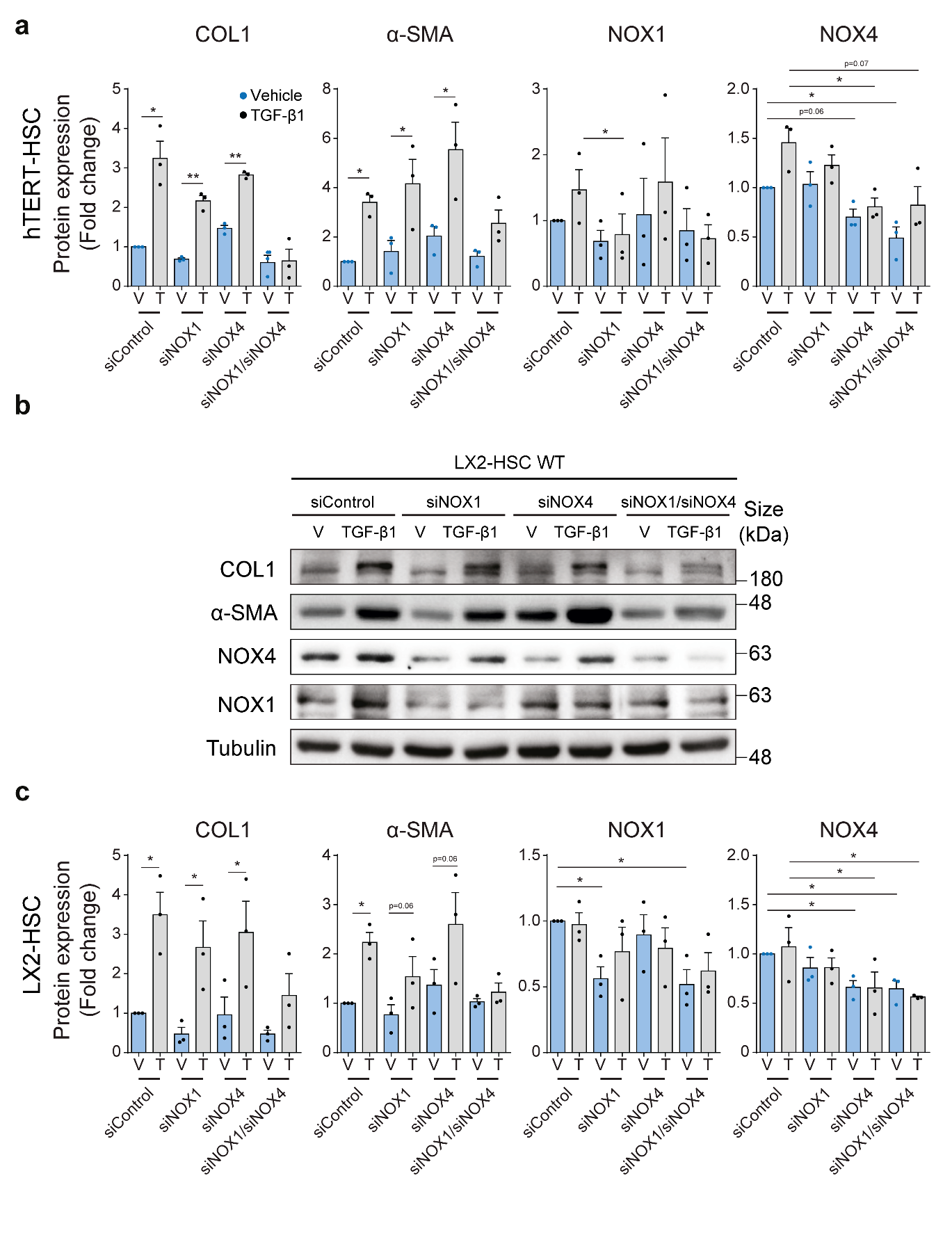


**Supplementary Figure 20. NOX4 and NOX1 are both necessary for fibroblast transdifferentiation induced by TGF-β. a.** Densitometry analysis of Western blot from hTERT-HSC cells from Figure 5i. Values are expressed as means±SEM. **b.** Changes in protein expression of α-SMA, COL1A1, NOX4 and NOX1 in LX2-HSC WT cells transfected with siRNA against NOX4, NOX1 or both, and treated with TGF-β1 (2 ng/ml) for 48 h. **c.** Densitometry analysis of western blot from LX2-HSC cells from panel b of this Supplementary Figure. Values are expressed as means±SEM of at least 3 cultures. *, p < 0.05; **, p < 0.01; compared with control or vehicle cells.

**
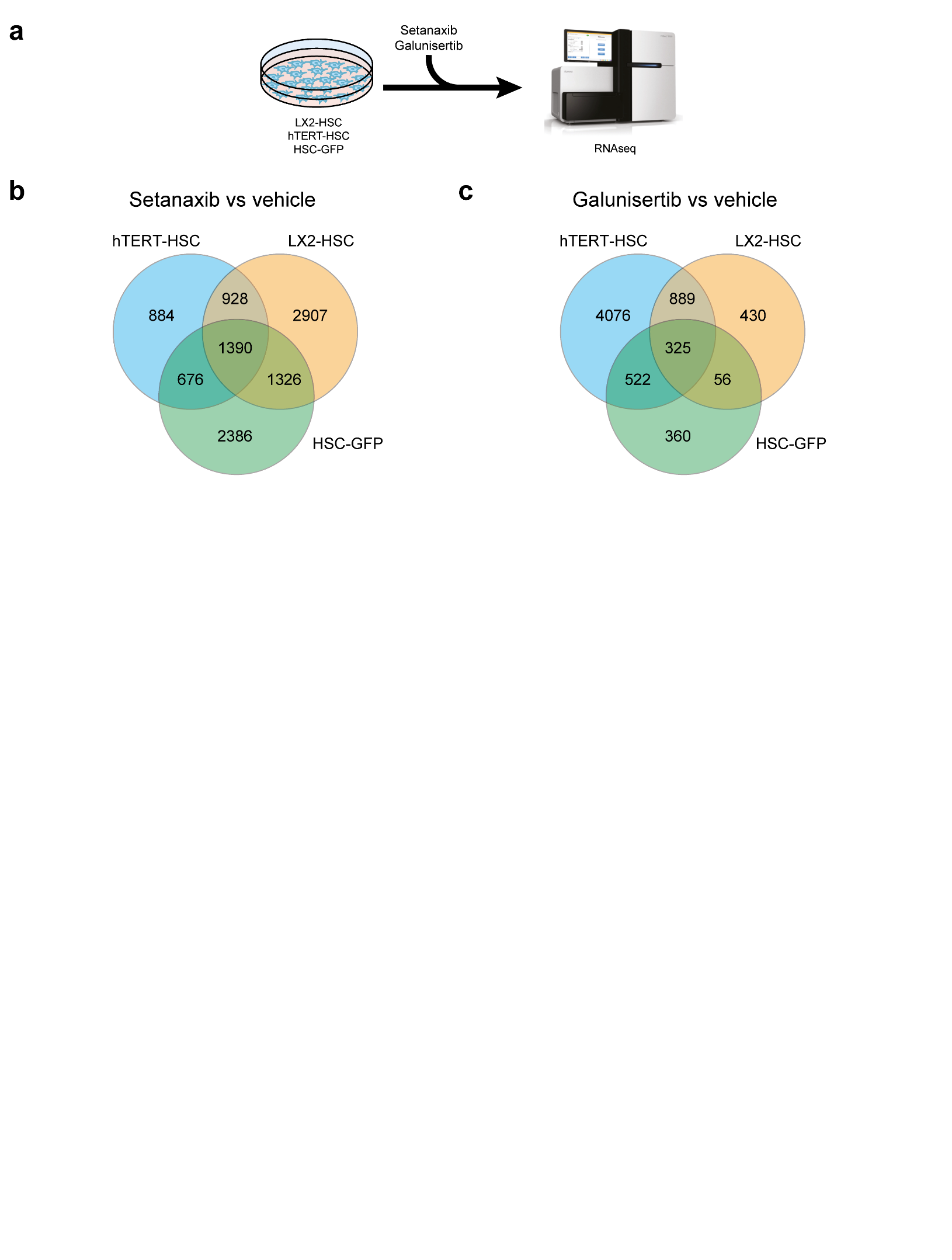
**

**Supplementary Figure 21. Transcriptomic effects of setanaxib and galunisertib in hepatic stellate cells (HSC) cell lines. a.** RNA-seq analyses were conducted in LX2-HSC, hTERT-HSC and HSC-GFP, displaying different degrees of basal activation to myofibroblasts. **b-c :** Venn diagrams showing differentially expressed genes in LX2-HSC, hTERT-HSC and HSC-GFP in response to setanaxib (40 µM) (b) or galunisertib (10µM) (d). Data obtained from RNAseq analysis.

**
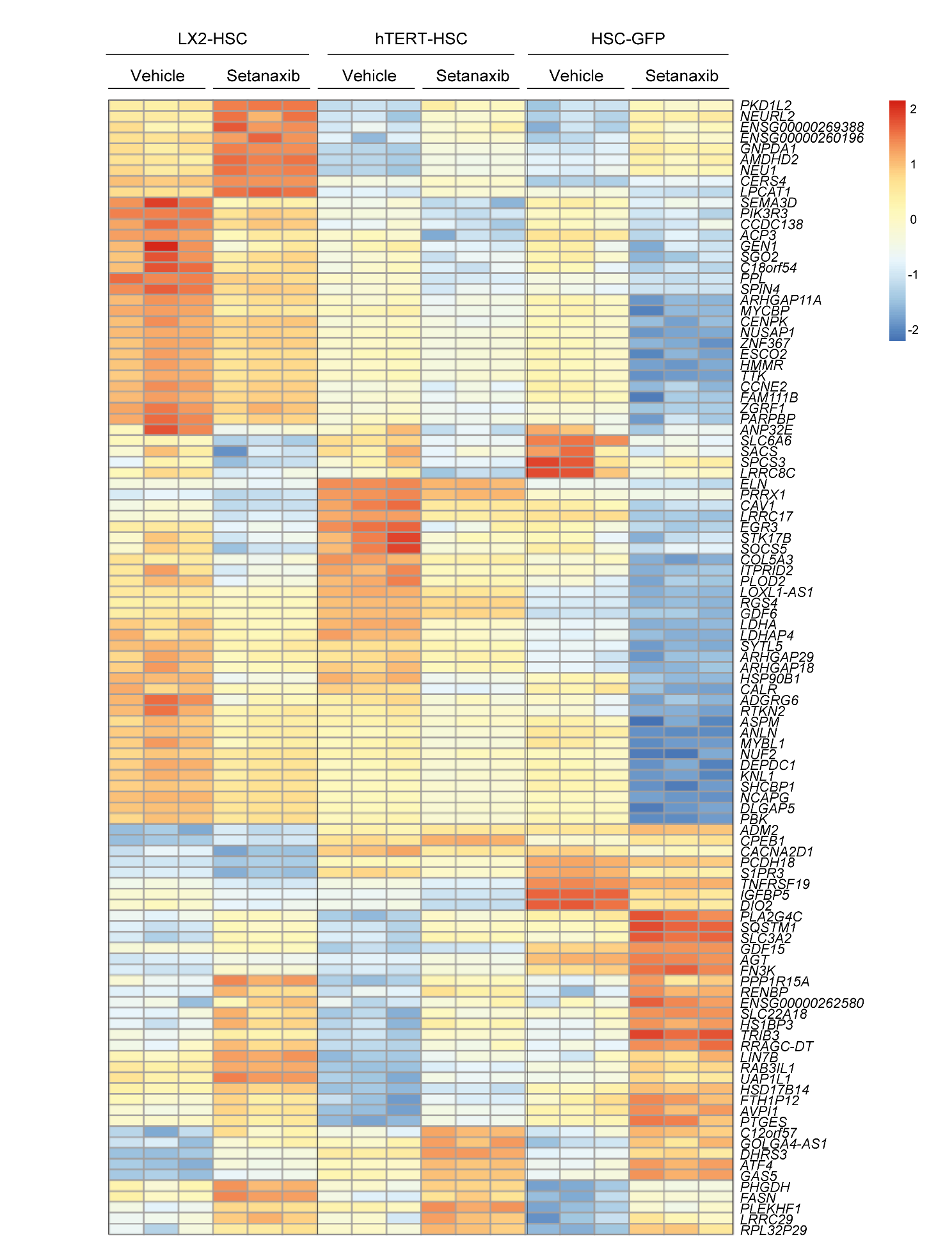
**

**Supplementary Figure 22. Transcriptomic effects of setanaxib in hepatic stellate cells (HSC) cell lines.** Heatmap showing changes in the expression of the Top 100 genes in LX2-HSC, hTERT-HSC and HSC-GFP in response to setanaxib. Data obtained from RNAseq analysis.

**
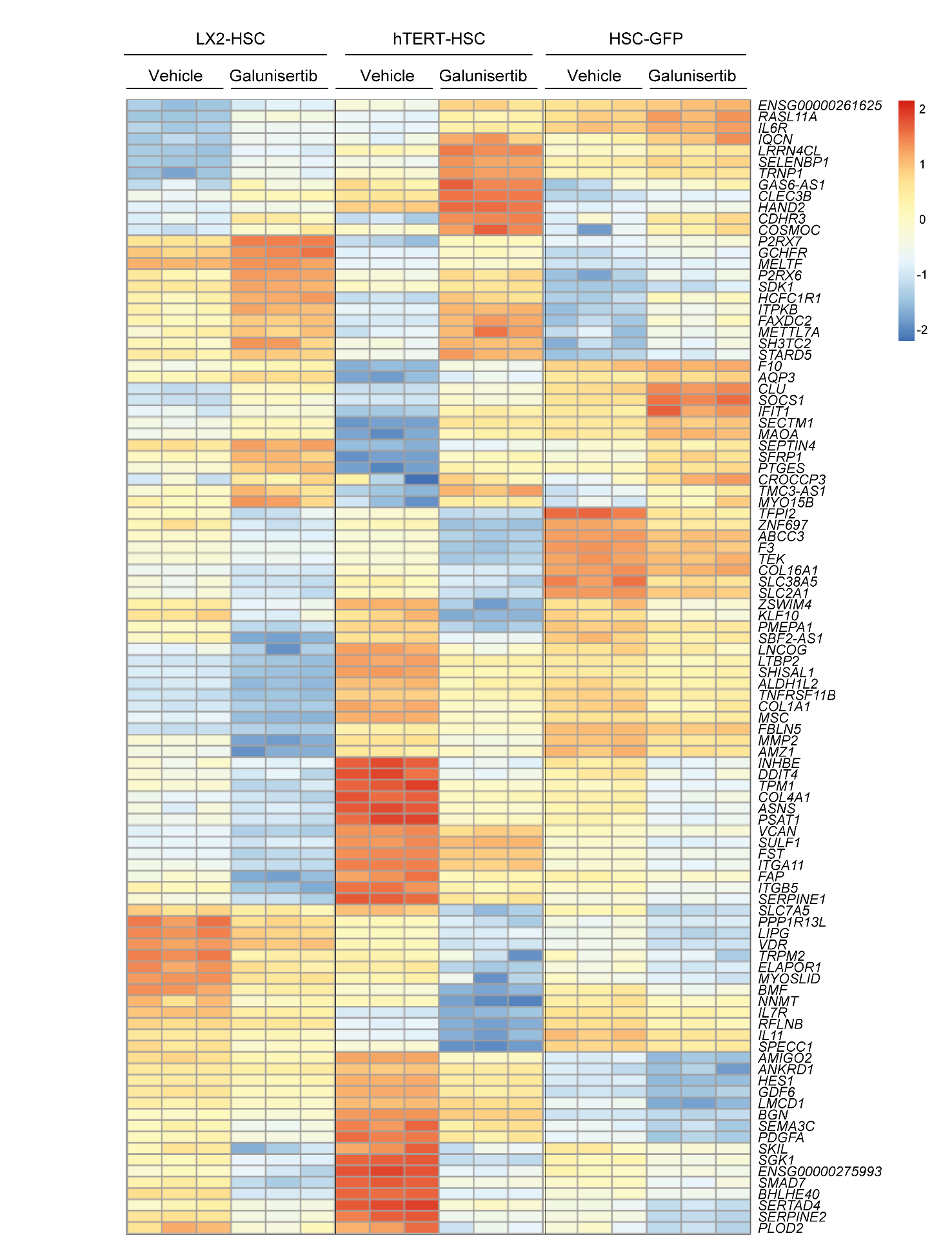
**

**Supplementary Figure 23. Transcriptomic effects of galunisertib in hepatic stellate cells (HSC) cell lines.** Heatmap showing changes in the expression of the Top 100 genes in LX2-HSC, hTERT-HSC and HSC-GFP in response to galunisertib. Data obtained from RNAseq analysis.

**
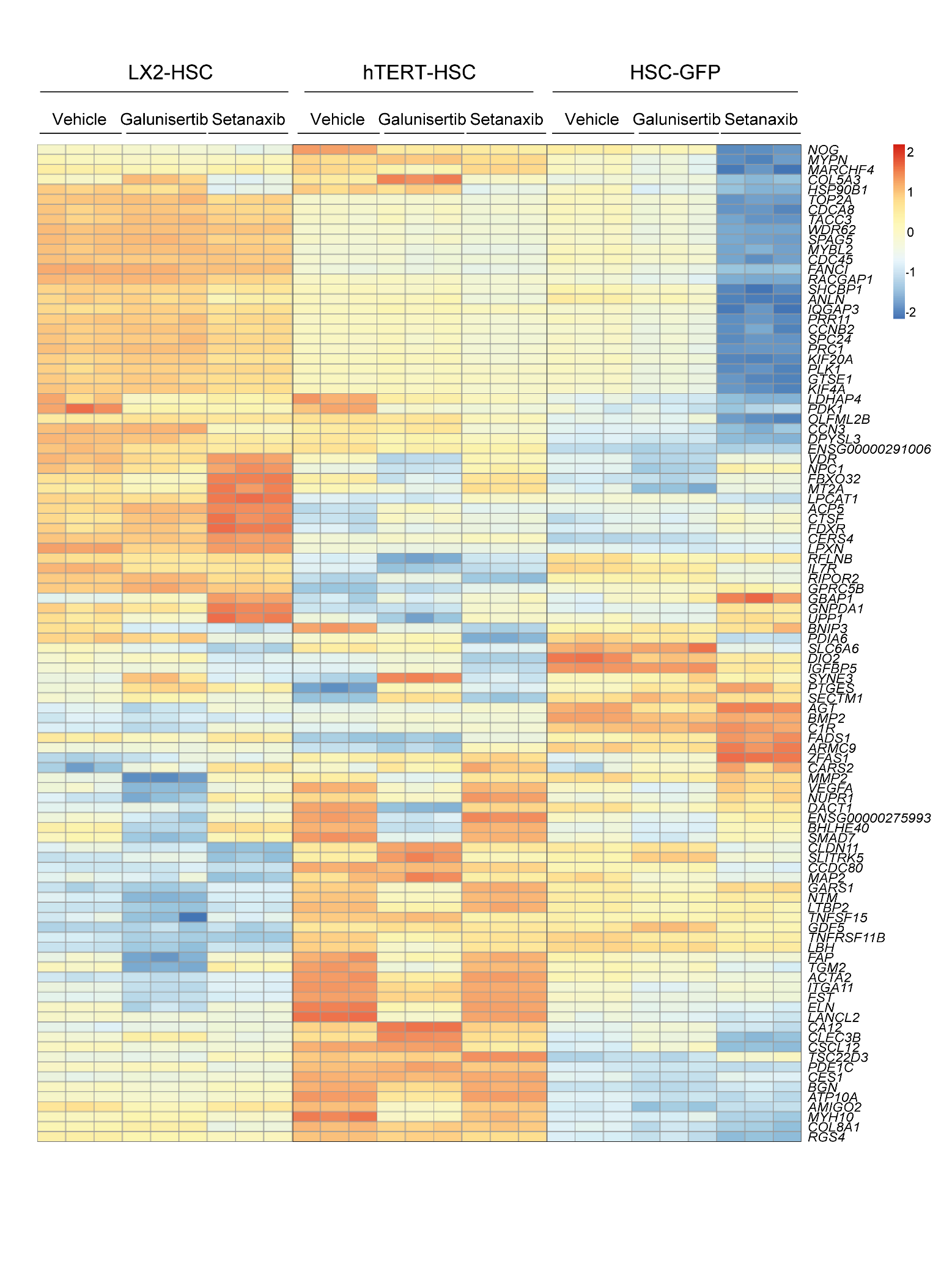
**

**Supplementary Figure 24. Setanaxib and galunisertib regulate pathways in hepatic stellate cell (HSC) lines.** Heatmaps showing changes in the expression of Top 100 genes. Data obtained from RNAseq analysis.

**
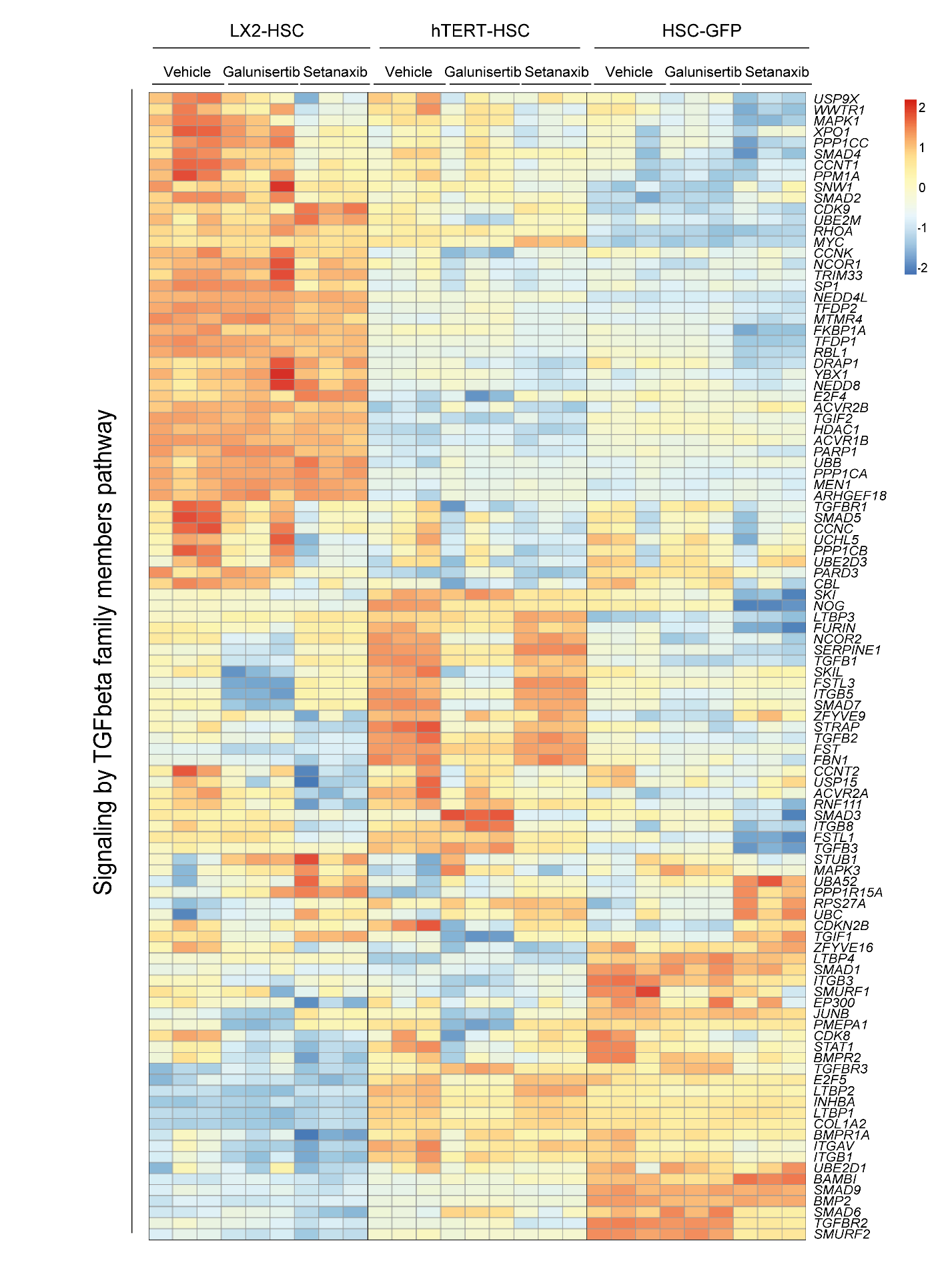
**

**Supplementary Figure 25. Setanaxib and galunisertib regulate pathways in hepatic stellate cell (HSC) lines.** Heatmaps showing changes in the expression of genes from the “Signalling by TGFbeta family members” pathway. Data obtained from RNAseq analysis.

**
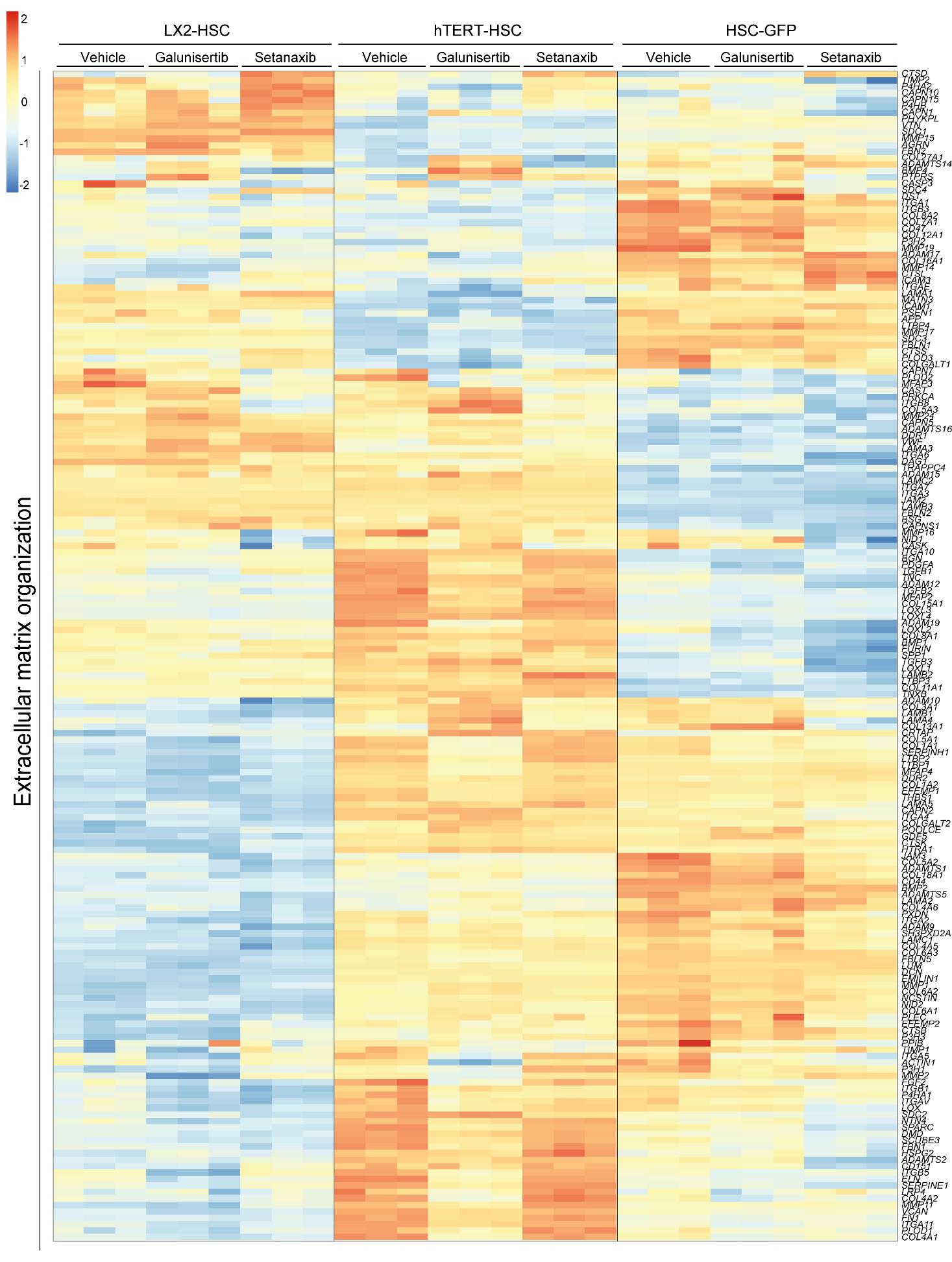
**

**Supplementary Figure 26. Setanaxib and galunisertib regulate pathways in hepatic stellate cell (HSC) lines.** Heatmaps showing changes in the expression of genes from the “Extracellular matrix” pathway. Data obtained from RNAseq analysis.

**
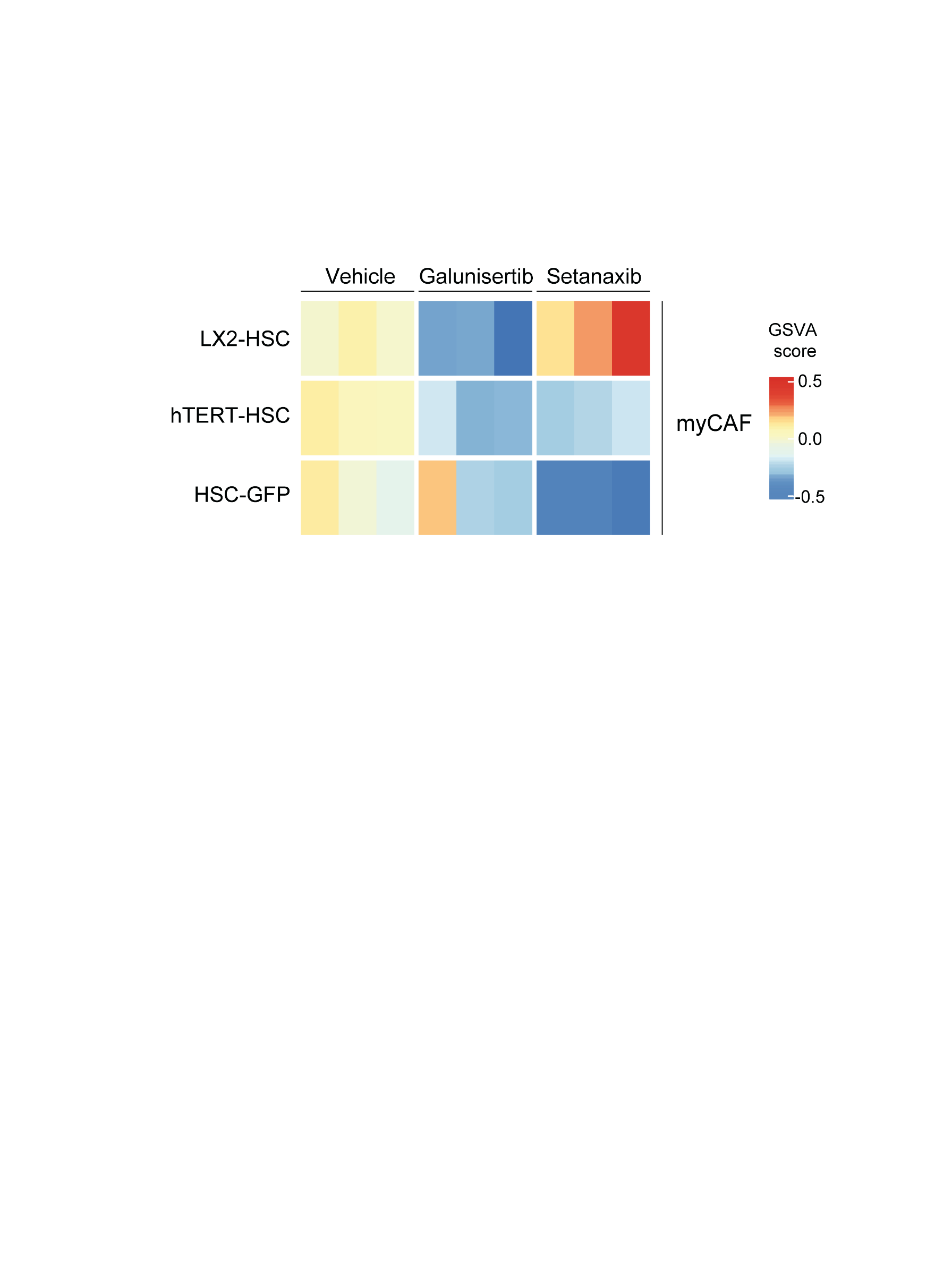
**

**Supplementary Figure 27. Setanaxib and galunisertib regulate pathways in hepatic stellate cell (HSC) lines.** Heatmap showing the enrichment in myCAF signatures of HSC cell lines treated with galunisertib and setanaxib, compared to the vehicle. Data obtained from RNAseq analysis.

**
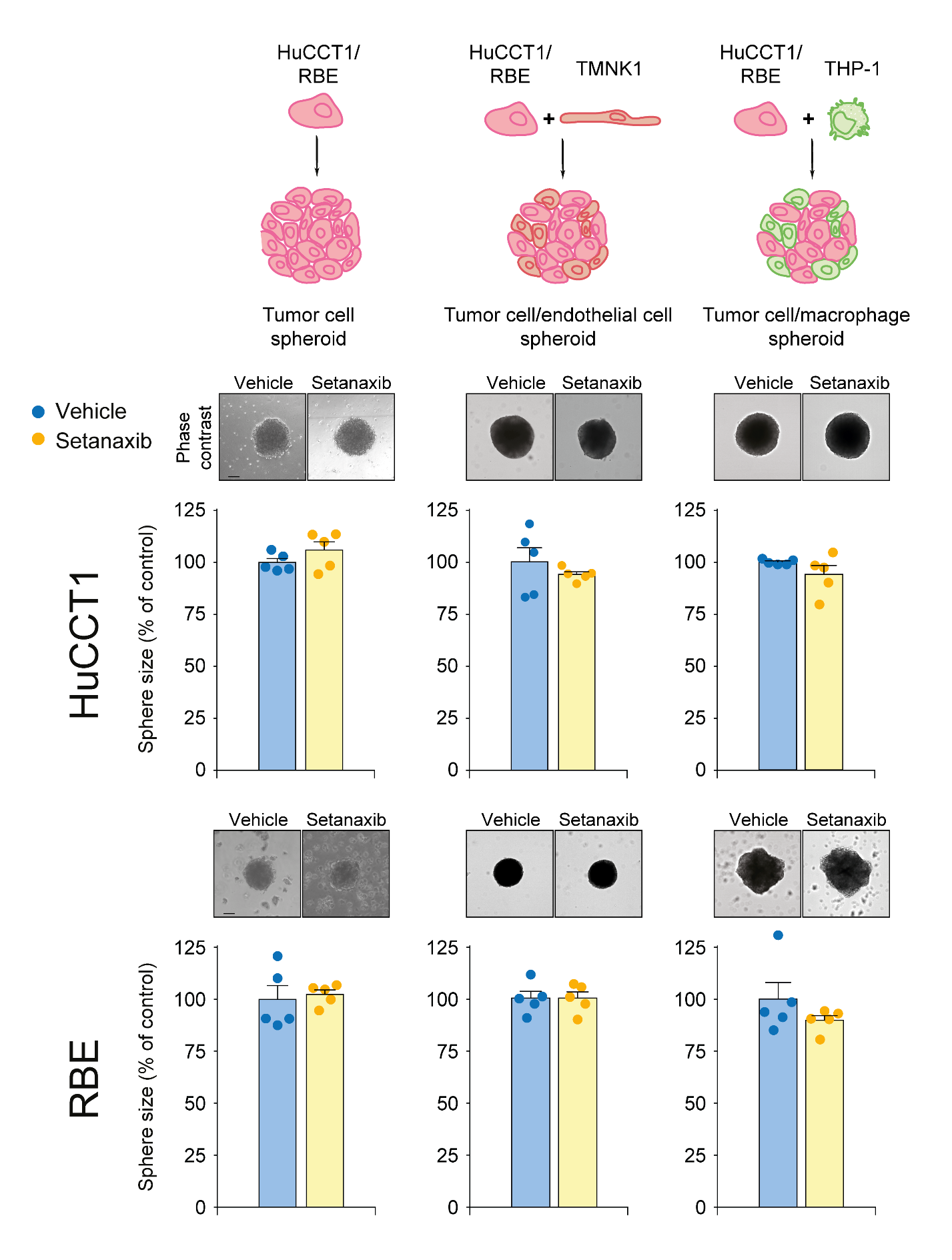
**

**Supplementary Figure 28. Dual NOX4/NOX1 inhibition does not impact cholangiocarcinoma tumour cell growth.** Size of spheres from HuCCT1 and RBE cells alone or in combination with TMNK1 or THP-1 cells after 4 days of treatment with setanaxib (40 µM). Representative phase contrast images of spheres at the time of size analysis are shown. Scale: 100 µm. Values are expressed as means ± SEM from 5 cultures.

**
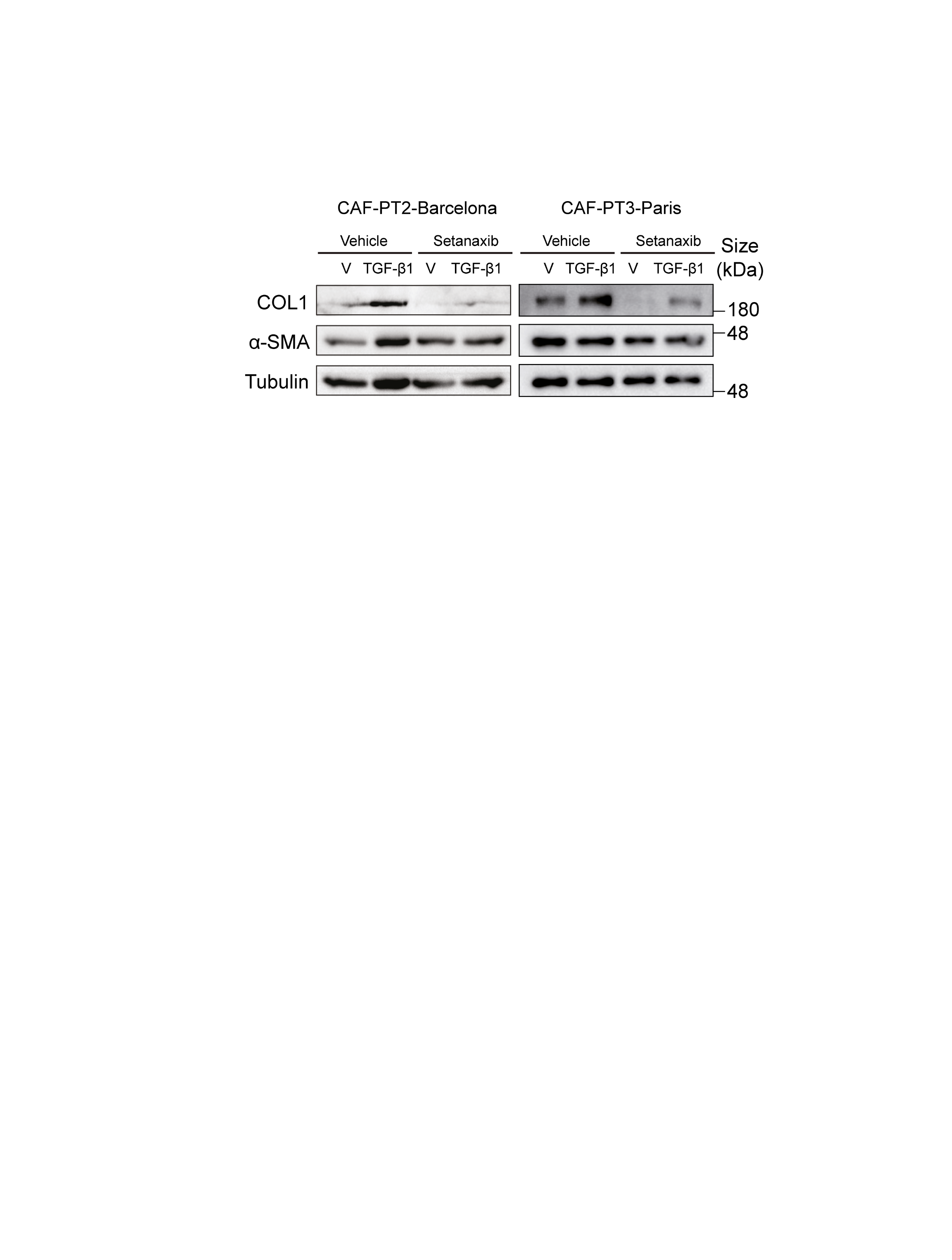
**

**Supplementary Figure 29. Dual NOX4/NOX1 inhibition impairs TGF-β induced transdifferentiation of cancer associated fibroblasts (CAF).** Representative images of Western blot analysis of collagen 1 (COL1) and α-SMA in CAF treated with TGF-β1 (2 ng/ml) or the vehicle in absence or presence of setanaxib (40 µM) for 48 hours.

**
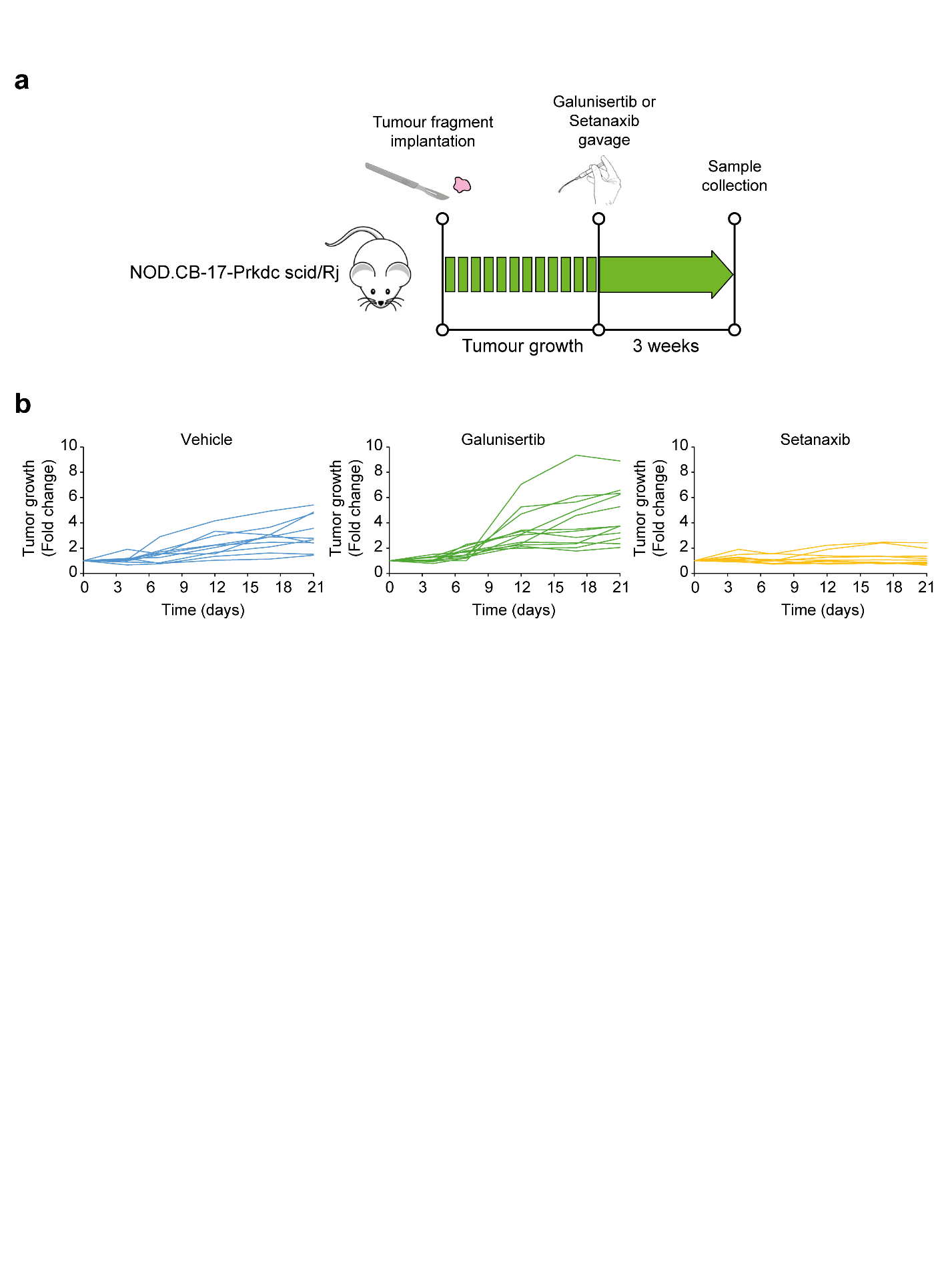
**

**Supplementary Figure 30. Impact of galunisertib and setanaxib treatment on tumour growth in an intrahepatic cholangiocarcinoma (iCCA) PDX model.** **a.** Tumour fragments from a previously characterized PDX (PDX153) were subcutaneously reimplanted into 6-week-old female NOD.CB-17-Prkdc scid/Rj. Once tumours reached an approximate volume of 150 mm3, mice started to be treated with galunisertib (150 mg/kg) or setanaxib (60 mg/kg). **b.** Tumour growth was evaluated over 21 days in mice receiving vehicle, setanaxib or galunisertib (n= 6-7 animals per group). Data from each individual tumour are shown in this figure.

**
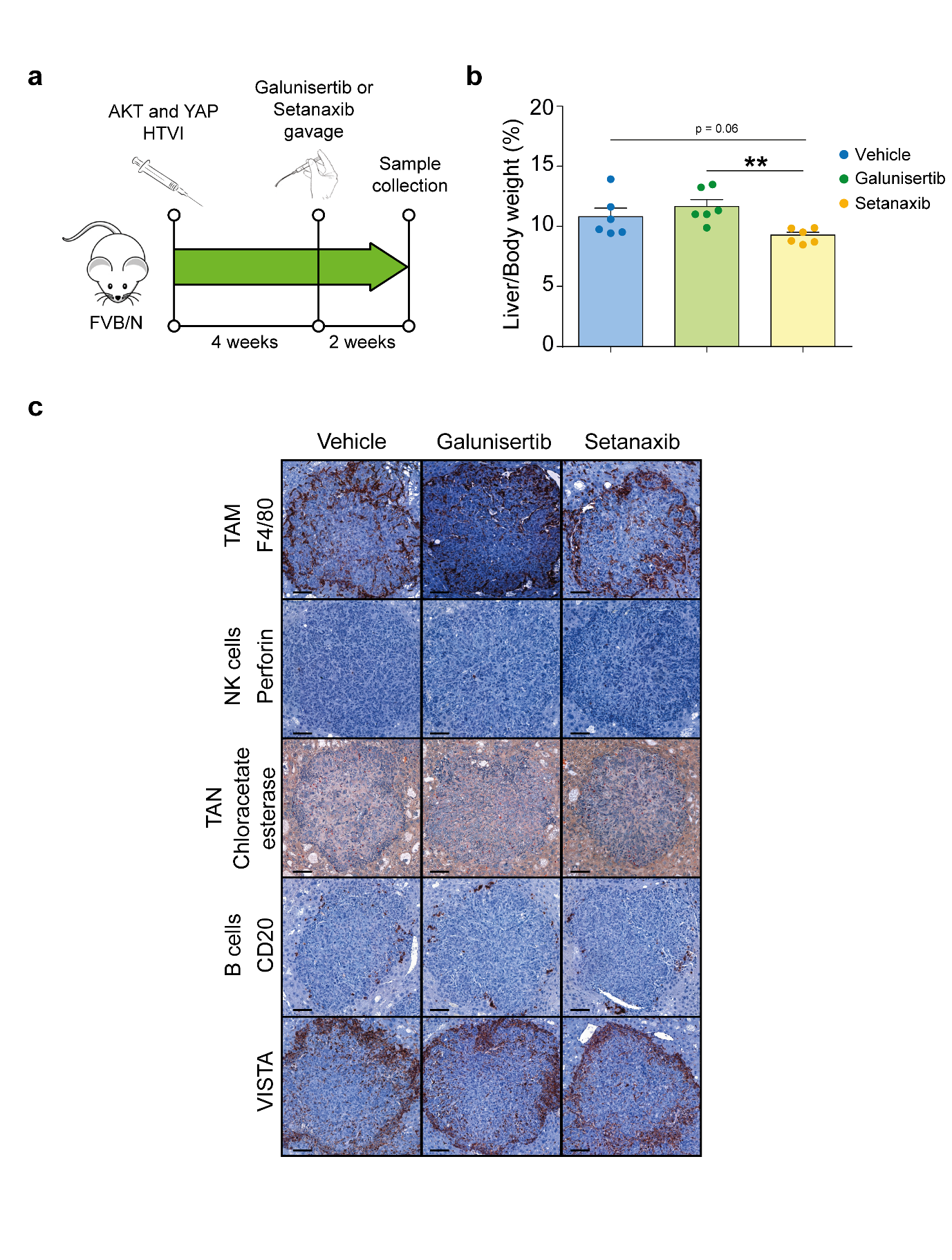
**

**Supplementary Figure 31. Impact of galunisertib and setanaxib treatments on the immune tumour microenvironment in the AKT-YAP intrahepatic cholangiocarcinoma (iCCA) model. a.** Schematic representation of the experimental model. Tumours were generated by hydrodynamic tail-vein injection (HTVI) of AKT and YAP vectors. After 4 weeks mice were treated with galunisertib (150 mg/kg), setanaxib (60 mg/kg) or vehicle *per os* for 2 weeks (n=7 mice per group). **b.** Liver to body weight ratio (%) of animals from panel a at term. **c.** Representative images of IHCs for F4/80, Perforin, Chloroacetate esterase, Cd20 and Vista, specific markers of different populations in the immune tumour compartment. TAM, Tumour associated macrophages; NK, Natural Killer; TAN, Tumour associated Neutrophils. Scale: 100 µm.
